# Experimental and Computational Study to Reveal the Potential of Non-Polar Constituents from *Hizikia fusiformis* as Dual Protein Tyrosine Phosphatase 1B and α-Glucosidase Inhibitors

**DOI:** 10.3390/md17050302

**Published:** 2019-05-22

**Authors:** Su Hui Seong, Duc Hung Nguyen, Aditi Wagle, Mi Hee Woo, Hyun Ah Jung, Jae Sue Choi

**Affiliations:** 1 Department of Food and Life Science, Pukyong National University, Busan 48513, Korea; seongsuhui@naver.com (S.H.S.); aditiwagle05@gmail.com (A.W.); 2 College of Pharmacy, Drug Research and Development Center, Catholic University of Daegu, Gyeongsan 38430, Korea; duchung1982fushico@gmail.com; 3 Department of Food Science and Human Nutrition, Chonbuk National University, Jeonju 54896, Korea

**Keywords:** *Hizikia fusiformis*, glycyrrhetinic acid, fucosterol epoxide, PTP1B, α-glucosidase

## Abstract

*Hizikia fusiformis* (Harvey) Okamura is an edible marine alga that has been widely used in Korea, China, and Japan as a rich source of dietary fiber and essential minerals. In our previous study, we observed that the methanol extract of *H. fusiformis* and its non-polar fractions showed potent protein tyrosine phosphatase 1B (PTP1B) and α-glucosidase inhibition. Therefore, the aim of the present study was to identify the active ingredient in the methanol extract of *H. fusiformis*. We isolated a new glycerol fatty acid (**13**) and 20 known compounds including 9 fatty acids (**1**–**3**, **7**–**12**), mixture of 24*R* and 24*S*-saringosterol (**4**), fucosterol (**5**), mixture of 24*R*,28*R* and 24*S*,28*R*-epoxy-24-ethylcholesterol (**6**), cedrusin (**14**), 1-(4-hydroxy-3-methoxyphenyl)-2-[2-hydroxy -4-(3-hydroxypropyl)phenoxy]-1,3-propanediol (**15**), benzyl alcohol alloside (**16**), madhusic acid A (**17**), glycyrrhizin (**18**), glycyrrhizin-6’-methyl ester (**19**), apo-9′-fucoxanthinone (**20**) and tyramine (**21**) from the non-polar fraction of *H. fusiformis*. New glycerol fatty acid **13** was identified as 2-(7′- (2″-hydroxy-3″-((5*Z*,8*Z*,11*Z*)-icosatrienoyloxy)propoxy)-7′-oxoheptanoyl)oxymethylpropenoic acid by spectroscopic analysis using NMR, IR, and HR-ESI-MS. We investigated the effect of the 21 isolated compounds and metabolites (**22** and **23**) of **18** against the inhibition of PTP1B and α-glucosidase enzymes. All fatty acids showed potent PTP1B inhibition at low concentrations. In particular, new compound **13** and fucosterol epoxide (**6**) showed noncompetitive inhibitory activity against PTP1B. Metabolites of glycyrrhizin, **22** and **23**, exhibited competitive inhibition against PTP1B. These findings suggest that *H. fusiformis*, a widely consumed seafood, may be effective as a dietary supplement for the management of diabetes through the inhibition of PTP1B.

## 1. Introduction

Diabetes mellitus (DM) is a serious chronic disease and an important public health problem. DM occurs when the pancreas does not produce enough insulin or when the body cannot effectively use insulin. In 2014, 422 million adults worldwide had DM and the prevalence of DM has been rising steadily for the past three decades [1]. Several underlying mechanisms contribute to the pathogenesis of Type 2 DM (T2DM), which include hereditary disease, gene mutation, and obesity, among others [2]. One of the therapeutic remedies to decrease post-prandial hyperglycemia in T2DM is by preventing the absorption of carbohydrates from food during consumption [3]. This can be achieved by inhibiting carbohydrate hydrolyzing-enzymes such as α-glucosidase and α-amylase in the digestive tract [4]. Inhibition of these enzymes causes delay in digestion of dietary polysaccharides, prolonging the overall carbohydrate digestion time, which ultimately reduces the rate of glucose absorption [5,6]. Other attractive targets in treating T2D are protein tyrosine phosphatases (PTPs), and intracellular PTP1B may be a target for drugs in T2D. PTP1B is mainly expressed in insulin-sensitive tissues and negatively regulates insulin signaling by acting on insulin receptors [7]. Insulin is the key regulator of glucose homeostasis, and insulin receptors are activated by auto-phosphorylation of the tyrosine residues in the insulin receptor activation loop, which causes signaling via insulin receptor substrate proteins, followed by downregulation of the insulin signaling pathway [8]. Interestingly, bioactive compounds that simultaneously block the activity of α-glucosidase and PTP1B exhibit synergistic effects to prevent hyperglycemia and hence effectively improve insulin sensitization [9]. Therefore, active compounds with this dual enzyme inhibition profile, such as geranylated flavonoids [9], Diels-Alder type adducts [10], and plastoquinones [11], may be promising scaffolds that could effectually contribute to the cure of T2D and suppress accompanied risks. In this era of lead drug development, the unique biochemical components of marine sources have gained much attention due to their diverse range of biological activities. Recently, marine-derived active compounds including bromophenols, phlorotannins, terpenes, and sterols were reported as potent PTP1B or α-glucosidase inhibitors [12].

*Hizikia fusiformis* (Harvey) Okamura is an edible brown seaweed in the Sargassaceae family that is widely distributed in the northwest coasts of the Pacific Ocean [13]. *H. fusiformis* has been reported to exhibit antioxidant [14], anti-inflammatory [15], and anti-Alzheimer’s disease activities [16] along with gastrointestinal protective effects [17]. In addition, *H. fusiformis* extract increased glucose uptake and activated insulin signaling pathway in muscle cells [18]. Several compounds from *H. fusiformis* have been isolated and shown to exhibit different bioactivities. Polysaccharide and glycoprotein from *H. fusiformis* showed protective effects against ethanol-induced gastric injury and acetaminophen-induced liver injury, respectively [17,19], and 4-hydroxyphenethyl alcohol from boiled *H. fusiformis* possessed whitening effects [20]. In our previous study to find anti-T2D materials from marine sources, we found that the crude methanol extract of *H. fusiformis* and its non-polar fractions showed potent PTP1B and α-glucosidase inhibition [15]. However, the active ingredient in the *H. fusiformis* extract has been unknown.

In this study, we isolated 21 compounds including fatty acids (FAs), sterols, phenolic compounds, homomonoterpene, and triterpenoid glycosides from the non-polar fraction of *H. fusiformis* and evaluated the PTP1B and α-glucosidase inhibitory activity of the isolated compounds. We also assessed the enzyme inhibitory activity of aglycone isomers of triterpenoid glycosides based on many references that describe triterpenoid as a representative scaffold for PTP1B inhibition [21]. To characterize the roles of the active compounds as a source of PTP1B and α-glucosidase inhibitors, detailed enzyme kinetic analysis and automated docking simulation were conducted.

## 2. Results

### 2.1. Structure Elucidation of Isolated Compounds

**Figure 1:**
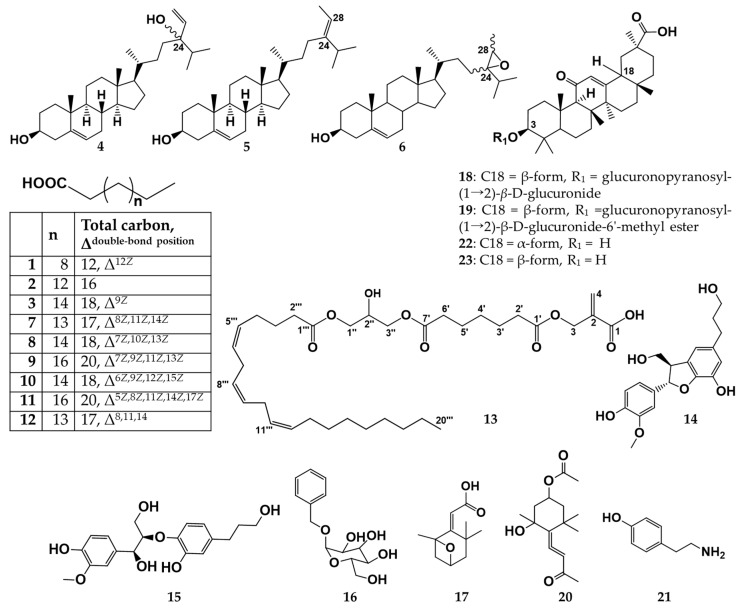
Structures of compounds isolated from *Hizikia fusiformis* and 18α and 18β-glycyrrhetinic acids.

Here we sought to identify the active ingredient in the *H. fusiformis* methanol extract responsible for the potent PTP1B and α-glucosidase inhibitory activity [15]. We isolated 21 compounds from the non-polar fraction, including a new glycerol FA 2-(7′-(2″-hydroxy-3″-((5*Z*,8*Z*,11*Z*)-icosatrienoyloxy)propoxy)-7′-oxoheptanoyl)oxymethylpropenoic acid (**13**) and 20 known compounds (Figure 1).

Compound **13** was obtained as a yellow syrup, and the HR-ESI-MS showed a *pseudo* molecular ion peak at *m/z* 607.3820 [M + H]^+^ (calculated for C_34_H_55_O_9_, 607.3846), confirming a molecular formula of C_34_H_54_O_9_. The ^1^H- and ^13^C-NMR spectra for **13** indicated the presence of diacylglycerol, aliphatic chain with three double bonds, alkane dicarboxylic acid, and 2-methylpropenoic acid, strongly suggesting a glycerol FA derivative.

**Figure 2:**
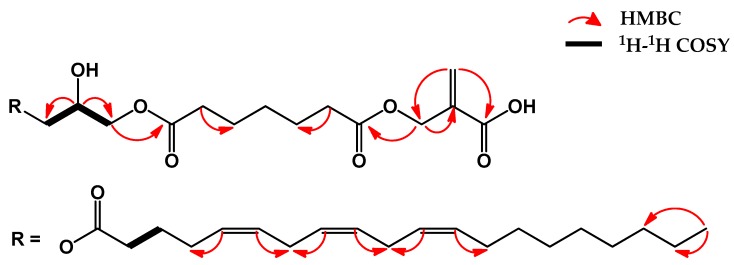
The key 2D NMR correlations for compound **13**.

The detailed ^1^H- and ^13^C-NMR spectra for **13** showed signals characteristic of an unsymmetrical diacylglycerol [unit: *δ*_H_ 4.16 (m, H3″), 3.64 (d, *J* = 5.38, H3″), 5.24 (m, H2″), 4.36 (dd, *J* = 3.7 and 12 Hz, H1″), 4.14 (m, H1″); *δ*_C_ 69.3 (C3″), 70.1 (C2″), 62.7 (C1″)]. As shown in Figure 2, the H-2″ showed correlation to H-3″ in the COSY spectrum, which was further connected to H-1″. The HMBC correlations of diacylglycerol were also observed from H-2″ to C-1″ and C-3″. The H-1″ and H-3″ of diacylglycerol were correlated with carbonyl carbon (*δ*_C_ 173.2, C-1‴) of eicosatrienoic acid and carbonyl carbon (*δ*_C_ 173.6, C-7′) of heptane-1,7-dioic acid by HMBC spectrum, respectively. 

Similarly, typical absorptions for acylglycerol and FA with aliphatic chains were detected in the FT-IR data: 3705.55-3680.48-3651.07 (O-H stretching), 3005.52-3022.39 (C-H olefins), 2957.79-2923.07-2892.7-2852.69 (aliphatic C-H stretching), 1737.07 (C=O stretching), and 1055.35-1033.18-1011.96 (C-O stretching) cm^−1^. 

One methyl (δ_c_ 14.2, C-20‴), 12 methylenes (δ_c_ 22.8, 25.1, 25.8 × 2, 27.3, 27.4, 29.3, 29.6, 29.8 × 2, 32.1, 34.4), six olefinic carbons (δ_C_ 128.2, 128.3, 129.8, 130.1 × 2, 130.4), and one carbonyl carbon at δ_C_ 173.2 in the ^13^C-NMR spectra and a methyl signal at *δ*_H_ 0.88, overlapping methylene protons between *δ*_H_ 1.25 and 2.31, and six olefinic protons (*δ*_H_ 5.36) in the ^1^H-NMR spectra explain the presence of eicosatrienoic acid. The ^1^H-NMR spectrum showed two methylene groups lying between three double bonds of eicosatrienoic acid at *δ*_H_ 2.79 (2H), which could be assigned to H-7‴ and H-10‴. The HMBC correlations of eicosatrienoic acid were also observed from H-5‴ to C4‴, from H6‴ to C-7‴, H-8‴ to C-7‴, H-9‴ to C-10‴, H-11‴ to C-10‴, H-12‴ to C-13‴, and from H-20‴ to C-18‴ and C-19‴. The geometry of the three double bonds in this FA moiety was presumed to be *cis*-form based on the ^13^C-NMR spectrum (δ_C_ 25.8, 27.3, 27.4). The signals of carbons next to a double bond usually appear at δ_C_ 27 to 28 in a *cis*-configuration, whereas those of a *trans*-configuration appear at δ_C_ 32 to 33 [22,23].

Five methylene characteristic signals including two low field values at *δ*_H_ 34.3 × 2, 25.0 × 2, and 29.2 and two carboxyl carbons at δ_C_ 173.6 × 2 indicated the presence of heptane-1,7-dioic acid [24]. The HMBC correlations were observed from C-3 (*δ*_C_ 69.3) of 2-methylpropenoic acid to carbonyl carbon (*δ*_C_ 173.6, C-1′) of heptane-1,7-dioic acid. The HMBC correlations of 2-methylpropenoic acid were detected from two olefin protons (δ_H_ 5.96, 6.42, H-4a and 4b) to C-1 (δ_C_ 170.2) and C-3 (δ_C_ 69.3) and from H-3 (δ_H_ 4.20) to C-2 (δ_C_ 136.3). 

Therefore, the chemical structure of compound **13** was identified as 2-(7′-(2″-hydroxy-3″- ((5*Z*,8*Z*,11*Z*)-icosatrienoyloxy)propoxy)-7′-oxoheptanoyl)oxymethylpropenoic acid. The chemical structure of compound **13** is described in Figure 1; Figure 2.

On the other hand, the ^1^H- and ^13^C-NMR spectra for compounds **1**, **3**, **7**, **8**, **9**, **10**, **11**, and **12** indicated the presence of aliphatic chains with more than one double bond, carboxylic acid, and methyl group, signifying unsaturated FAs (Figures S7, S11, S16, S18, S20, S22, S24, and S26). The molecular weight of these compounds was confirmed by EI-MS analysis. The geometry of the double bonds in these FAs was presumed to be *cis*-form based on the ^13^C-NMR spectrum, as described above [22]. Precise chemical structures of these FAs were identified as (*Z*)-hexadec-12-enoic acid (**1**), (*Z*)-octaec-9-enoic acid (**3**), (8*Z*,11*Z*,14*Z*)-heptadeca-8,11,14-trienoic acid (**7**), (7*Z*,10Z,13*Z*)-octadeca-7,10,13-trienoic acid (**8**), (7Z,9*Z*,11*Z*13*Z*)-eicosa-7,9,11,13-tetraenoic acid (**9**), (6*Z*,9*Z*,12*Z*,15*Z*)-octadeca-6,9,12,15-tetraenoic acid (**10**), (5*Z*,8*Z*,11*Z*,14*Z*,17*Z*)-eicosa- 5,8,11,14,17- pentaenoic acid (**11)**, and (8*Z*,11*Z*,14*Z*)-heptadeca-8,11,14-trienoic acid (**12**), respectively, by comparison with previously published data [23]. 

The ^1^H-NMR spectra of compounds **4**–**6** exhibited olefin methine, one oxygenated methine, five methyl signals, indicating a steroidal structure (Figures S13–S15). The ^13^C-NMR spectrum of **4**–**6** showed 29 carbon signals including olefin methine carbon (C-6), one oxygenated methine carbon (C-3), two quaternary carbons (C-10 and 13), seven methine carbons (C-8, 9, 13, 14, 17, 20, and 25), 10 methylene carbons (C-1, 2, 4, 7, 11, 12, 15, 16, 22, and 23), and five methyl carbons (C-18, 19, 21, 26, and 27). By comparison with the literature [25,26], structure of **5** was identified as fucosterol, very common sterol in algae. The additional olefin methine and exo-methylene carbon signals between C-24 and C-28 were observed in ^13^C-NMR spectra of sterol **4**. In case of sterol **6**, epoxy signals were observed at δ_c_ 66.48 and 66.38 (C-24) and δ_c_ 56.88 and 56.92 (C-28). The duplicate signals (C-17: δ_C_ 56.07/55.87 ppm, C-24: 89.23/89.18, C-28: 137.38/137.27, and C-29: 116.44/116.38) in the ^13^C-NMR spectrum of sterol **4** were in accordance with the occurrence of the two C-24 epimers (Figure S13). Similarly, the duplicate signals (C-17: δ_C_ 56.88/56.66 ppm, C-24: 66.48/66.38, C-25: 32.06/31.81, C-28: 57.08/56.92) in the ^13^C-NMR spectrum of sterol **6** were in accordance with the occurrence of the two C-24/C-28 epimers (Figure S15). The configuration at C-24/C-28 of compound **6** was determined by comparison with published data [26]. Finally, the chemical structures of sterols **4** and **6** were identified as mixture of 24*R* and 24*S*-saringosterol (**4**) and mixture of 24*R*,28*R* and 24*S*,28*R*-epoxy-24-ethylcholesterol (**6**), respectively, by interpretation of spectroscopic data and comparison with literature [25,26]. 

The ^13^C-NMR spectra of compounds **14** and **15** exhibited benzylic methylene carbon of *n*-propanol chain. In the ^1^H- and ^13^C-NMR spectra of **14** (Figure S28), one aryl-substituted benzofuran methine carbon (δ_C_ 88.68), one oxymethylene carbon (δ_C_ 65.12), and one methoxyl carbon (δ_C_ 56.33) were observed. In the ^1^H- and ^13^C-NMR spectra of **15** (Figure S29), one phenoxy methine proton (δ_H_ 4.00), one benzyl hydroxymethine proton (δ_c_ 4.87), one aromatic methoxyl carbon (δ_C_ 56.43), one oxymethylene carbon and two protons (δ_C_ 62.2 and δ_H_ 3.67 and 3.46) were observed. These spectral data and published data [27,28] establish the structures of **14** and **15** as cedrusin (**14**) and 1-(4-hydroxy-3-methoxyphenyl)-2-[2-hydroxy-4- (3-hydroxypropyl)phenoxy]-1,3- propanediol (**15**), respectively. 

In addition, the ^13^C-and ^1^H-NMR spectra of compounds **16**–**21** and previously published data [29,30,31,32,33,34] establish the structures of **16**–**21** as benzyl alcohol alloside (**16**), madhusic acid A (**17**), glycyrrhizin (**18**), 18β-glycyrrhetinic acid-3-*O*-*β*-d-glucuronopyranosyl-1(→2)-*β*-d-glucuronide -6’-methyl ester (**19**), apo-9′-fucoxanthinone (**20**), and tyramine (**21**), respectively. 

Notably, compounds **7**–**12**, **14**–**17**, **19**, and **20** were isolated from *H. fusiformis* for the first time.

### 2.2. PTP1B and α-Glucosidase Inhibitory Activity of the Isolated Compounds from H. fusiformis

As a result, all FAs showed potent PTP1B inhibition with IC_50_ values in the range of 4.86–49.39 μM. Among the FAs, compound **7** showed the highest inhibitory activity followed by compound **13** and **1** with IC_50_ values of 4.86 ± 1.36, 4.92 ± 0.01, and 6.59 ± 0.09 μM, respectively. Among the sterols, compound **6**, which is an epoxide of fucosterol (**5**), exhibited 3 times stronger PTP1B inhibitory activity than **5** (IC_50_ = 16.70 ± 0.36 and 50.58 ± 1.86 μM for sterols **6** and **5**, respectively). However, sterol **4** showed no activity under the tested concentration. Among the triterpenoid derivatives, compound **19**, which is a 6′-methyl ester of compound **18**, showed 2.2 times stronger PTP1B inhibition than compound **18** (IC_50_ = 110.33 ± 0.39 and 49.39 ± 1.39 μM for compounds **18** and **19**, respectively). Due to the moderate effect of triterpenoid glycoside **18**, we further evaluated the activity of the metabolites of **18** including 18α-glycyrrhetinic acid (**22**) and 18β-glycyrrhetinic acid (**23**). As shown in Table 1, **22** showed potent inhibitory activity against PTP1B having an IC_50_ value of 10.40 ± 0.75 µM followed by **23** with an IC_50_ of 26.07 ± 0.59 µM with ursolic acid as a positive control (IC_50_ = 7.31 ± 0.16 µM). In contrast, other compounds (**15**–**17**, **20**, and **21**) exhibited weak or no inhibitory activity against PTP1B.

In the case of α-glucosidase, compounds **22** and **23** exhibited effective inhibitory activity with IC_50_ values of 113.30 ± 0.70 and 128.72 ± 3.88 µM, respectively, which are slightly less than the positive control acarbose (IC_50_ = 158.41 ± 1.05 µM). However, compounds **18** and **19** showed no activity under the tested concentration. Interestingly, unsaturated FAs C20:4 (Δ^7,9,11,13^) (**9**) and C17:3 (Δ^8,11,14^) (**12**) showed potent inhibition against α-glucosidase with IC_50_ values of 34.85 ± 2.39 and 43.90 ± 0.77 µM, respectively. In addition, neolignan **14** and trace amine **21** also showed moderate inhibition with IC_50_ values of 133.84 ± 3.86 and 273.23 ± 5.65 µM, respectively. In contrast, other compounds exhibited weak or no activity against α-glucosidase inhibition.

### 2.3. Enzyme Kinetic Analysis of Active Compounds with PTP1B

**Figure 3:**
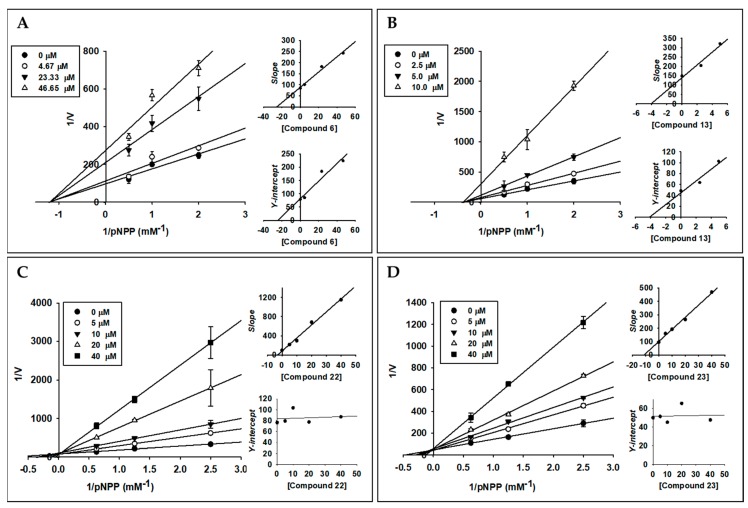
Enzyme kinetic analysis of compounds **6** (**A**), **13** (**B**), **22** (**C**), and **23** (**D**) using Lineweaver-Burk plots and its secondary plots (1/*V_max,app_* (*Y*-*intercept*) and *K_m,app_*/*V_max,app_* (*slope*) of the respective linear regression of Lineweaver-Burk plot).

Compounds **6**, **13**, **22** and **23** were subjected to enzyme kinetic study, since these compounds demonstrated potent activity against PTP1B. According to the Lineweaver-Burk plot and secondary plot of *y-*intercept (Table 1 and Figure 3), compounds **22** and **23** showed general competitive type inhibition against PTP1B, whereas compounds **6** and **13** showed inhibition in a non-competitive manner. The binding constant of inhibitor with enzyme-substrate complex (*K_iu_*) and free enzyme (*K_ic_*) was determined using the secondary plot of 1/*V_max,app_* (*Y*-*intercept*) and *K_m,app_*/*V_max,app_* (*slope*) of the respective linear regression of Lineweaver-Burk plot, respectively. As shown in Figure 3, *K_ic_* values for the inhibition of PTP1B were 3.17 and 10.17 µM for **22** and **23**, respectively, and *K_iu_* values for inhibition of PTP1B by **6** and **13** were 24.43 and 4.13 µM, respectively.

### 2.4. Molecular Docking Simulation in PTP1B Inhibition

**Figure 4:**
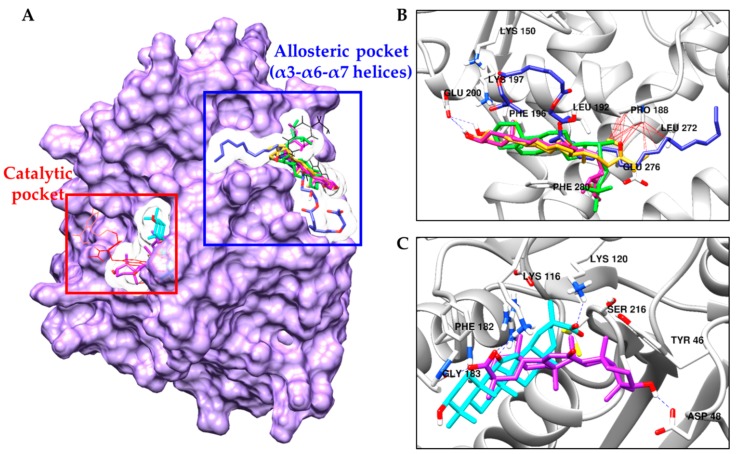
Best docked models of compounds from *H. fusiformis* in the catalytic (**A** and **C**) and allosteric (**A** and **B**) pocket of PTP1B (1T49) along with positive ligands, compounds A (red line) and B (black line). Fucosterol (**5**), 24*R*,28*R*-epoxy-24-ethylcholesterol (**6a**), 24*S*,28*R*-epoxy-24-ethylcholesterol (**6b**), compound **13**, and 18α and 18β-glycyrrhetinic acids (**22** and **23**) are shown as pink, yellow, green, blue, cyan, and purple stick, respectively. The residues forming inter H-bond with the ligands are shown as blue dotted lines. Hydrophobic interactions between Pro188 residue and compounds are shown as black lines.

Due to the potent inhibitory activity of **5**, **6**, **13**, **22**, and **23** against PTP1B, we conducted computational docking analysis using these compounds to evaluate binding affinities and aspects. Sterols **5** and **6** and compound **13** are well docked into the allosteric pocket of PTP1B (α3, α6, and α7 helices), whereas triterpenoids **22** and **23** are docked into the catalytic site (Figure 4). Because **6** is mixture of 24*R*,28*R* and 24*S*,28*R*-epoxy-24-ethylcholesterol (**6a** and **6b**), we also compared the binding aspect between the two isomers. Compound A (catalytic inhibitor) and compound B (allosteric inhibitor) were used as positive controls to verify the docking protocol.

**Figure 5:**
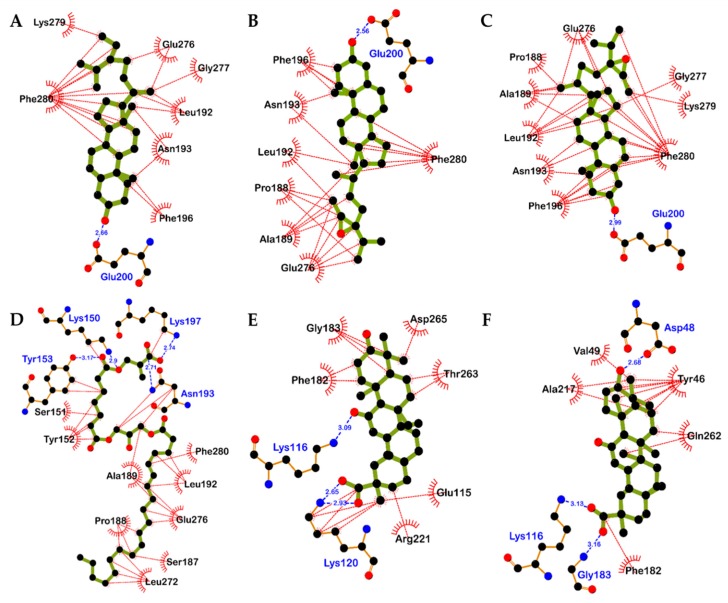
Detailed binding interactions visualized by docking simulation for the compounds **5** (**A**), **6a** (**B**), **6b** (**C**), **13** (**D**), **22** (**E**), and **23** (**F**).

As shown in Figure 4; Figure 5, best fitted models of **5**, **6a**, and **6b** interacted with Glu200 in the α3 helix via H-bond and surrounded by hydrophobic residues in α3 (Phe196, Asn193, and Leu192) and α6 (Glu276 and Phe280) helices of enzyme with negative B-scores of −8.10, −7.90, and −8.66 kcal/mol, respectively. Interestingly, one difference was observed between the **5**-PTP1B complex and the **6a**/**6b**-PTP1B complex. Both **6a** and **6b** interacted with Pro188 residue via a hydrophobic bond (Figure 5B,C), but the aliphatic side chain of **5** did not reach near Pro188 (Figure 5A). Docking examination showed that **13** interacted with the allosteric site of the enzyme by positioning the long aliphatic chain toward the center of α3 and α6 helices of the enzyme, whereas the methacrylic acid moiety of **13** was located at the edge of the α3 helix and interacted with Asn193 and Lys197 via H-bond interactions (Figure 5D). Although **13** showed strong potency against PTP1B inhibition in vitro, its binding affinity was poor due to the long aliphatic chain. However, four tight H-bond interactions between compound **13** and PTP1B residues including Tyr153, Lys150, Lys197, and Asn193 may play key roles in PTP1B inactivation.

In contrast to sterols and compound **13**, the best docked models of compounds **22** and **23** were placed into the catalytic site of PTP1B. As shown in Figure 4C, binding orientations of **22** and **23** were slightly different. The PTP1B-**22** complex had a negative B-score (Table 2) of −9.09 kcal/mol with two H-bonds with Lys116 and Lys 120 as well as a salt-bridge interaction with Lys120 residue. Hydrophobic interactions between **22** and Phe182, Gly183, Arg221, Glu115, Thr263, Asp265, and Lys120 residues were also observed (Figure 5E). However, the PTP1B-**23** complex had a B-score of –8.90 kcal/mol with two H-bonds with Gly183 and Asp48 residues and a salt-bridge interaction between carboxyl moiety of **23** and Lys116. As shown in Figure 5F, **23** was surrounded by Tyr46, Val49, Ala217, Phe182, and Gln262 residues via hydrophobic interaction.

## 3. Discussion

Growing evidence has linked PTP1B with insulin resistance, T2DM, and obesity. Numerous studies have revealed that PTP1B negatively controls leptin and insulin signaling pathways [12]. Therefore, a considerable effort has been expended on generating small molecule inhibitors of PTP1B to promote the insulin signaling pathway in insulin resistant states. By following the conventional method of producing inhibitors that target the catalytic site of an enzyme, many selective and reversible PTP1B inhibitors were discovered [35]. However, these small molecule inhibitors, which often possessed phospho-Tyr mimetic moieties, were highly charged and lacked oral bioavailability, showing limitations in their potential for drug development. Therefore, the development of an allosteric inhibitor is urgently needed to develop orally bioavailable inhibitors of PTP1B [36]. We previously demonstrated that non-polar fractions of *H. fusiformis* methanol extract showed potent PTP1B and α-glucosidase inhibition [15]. Various non-polar components such as 24-ketocholesterol, fucosterol, 24,28-epoxyfucosterol, fucoxanthin, and saringosterol have been isolated from this seaweed [14,37]. However, the systematic extraction and isolation of compounds from *H. fusiformis* as well as the mechanisms of PTP1B and α-glucosidase inhibition through detailed enzyme kinetics and molecular docking simulation have not been reported. In this study, we isolated one new and 20 known compounds from the non-polar fraction of *H. fusiformis* methanol extract and evaluated the PTP1B and α-glucosidase inhibitory activity of the isolated compounds. Enzyme assay results revealed that unsaturated and saturated FAs, sterols, and triterpenoid glycosides showed good inhibitory activity against PTP1B. Shibata et al. reported that unsaturated FAs at 10 μM drastically inhibited PTP1B, whereas saturated FAs showed moderate inhibition [38]. Interestingly, in rat adipocytes, long-time treatment of saturated free FAs inhibited insulin-stimulated glucose uptake, but short-time treatment enhanced glucose transport [39]. Similarly, in our results, unsaturated FAs showed significantly strong PTP1B inhibitory activity with IC_50_ values in the range of 4.86–16.43 μM. In contrast, among saturated FAs, palmitic acid (**2**) showed moderate activity with an IC_50_ value of 49.39 ± 1.39 μM. In addition, C17:3 (Δ^8,11,14^) (**7**) and the new compound **13** showed notable inhibition among the isolated 22 compounds. Together, our results and the previously reported data suggest that FAs could be an important factor responsible for T2DM. 

A previous study showed that fucosterol (**5**) from *Pelvetia siliquosa* possessed anti-diabetic activity in streptozotocin-induced Sprague-Dawley rats [40]. Another report demonstrated that **5** is a non-competitive PTP1B inhibitor in vitro and improved insulin resistance by inhibition of PTP1B and stimulation of insulin signaling pathway in insulin-resistant HepG2 cells [41]. However, information on the biological activity of fucosterol epoxide (**6**) is limited. As shown in Table 1, **5** and its epoxide (**6**) showed PTP1B inhibitory activities. Interestingly, **6** showed 3 times stronger activity than **5**. Enzyme kinetic analysis using Lineweaver-Burk plot and its secondary plot and computational docking analysis demonstrated that **5**, **6**, and **13** are non-competitive inhibitors and well docked into the allosteric pocket placed ~20 Å away from the catalytic site of PTP1B [42]. Best fitted models of **5** and **6** interacted with Glu200 in the α3 helix via H-bond and surrounded by hydrophobic residues in α3 and α6 helices of enzymes such as Phe280, Phe196, Leu192, and Ala189. However, the lack of interaction between compound **5** and Pro188 explains its lower PTP1B inhibitory potency compared to **6**. 

PTP1B enzyme exists in two conformations: open and closed forms. In the open form, the WPD loop, which contains Trp179‒Asp181 residues, is beside the catalytic site to form an open-binding pocket, which is accessible for the substrate. In the closed-form, the WPD loop covers the substrate-binding site of the enzyme, forming a catalytically competent state. For the WPD loop to close, Pro188-Phe191-Leu192 residues must move to accommodate Trp179 [42]. However, this movement is blocked by compound **6** directly via hydrophobic interaction. Thus, the allosteric inhibitor **6** could prevent the movement of the WPD loop and maintain the loop in an open (inactive) form. In the case of **13**, this compound also hydrophobically interacted with Pro188 residue with four H-bond interactions with Tyr153, Lys150, Lys197 and the key allosteric site residue Asn193. These interactions may play critical roles in PTP1B inactivation in the PTP1B-**13** complex.

We also found that triterpenoid glycosides **19** and **18** are effective and moderate PTP1B inhibitors, respectively. Compound **19**, which is a 6′-methyl ester of **18**, showed 2.2 times stronger PTP1B inhibition than compound **18**. In addition, 18α and 18β-glycyrrhetinic acids (**22** and **23**), metabolites of **18** and **19**, are stronger PTP1B inhibitors compared with **18** and **19**. Although the PTP1B inhibitory activities of **22** and **23** were previously described by Na et al. [43], the inhibitory mechanisms and structure-activity relationships have not been reported. In our enzyme kinetic and computational study, triterpenoids **22** and **23** showed competitive inhibition activity against the PTP1B enzyme and were strongly fitted into the catalytic site of the enzyme. Due to the different configuration (α and β) of the hydrogen atom at C-18 position, binding aspect was slightly changed. The carboxyl moiety of **22** and Lys120, Lys116, Tyr46 and Ser216 residues interacted via hydrogen bonds including salt bridge and conventional H-bonds, respectively. These interactions may contribute to the strong PTP1B inhibitory activity of **22**. 

Regarding α-glucosidase inhibitory activity, **9** showed notable inhibitory activity among the FAs. However, we could not define the correlation among α-glucosidase inhibitory activity, unsaturation, and number of carbon atoms. In addition, sterols and triterpenoid glycosides did not show any inhibition against α-glucosidase under the tested concentrations, but triterpenoids **22** and **23** exhibited similar effect with the positive control, acarbose.

This study has four important findings: (i) the isolation and structure identification of compounds from *H. fusiformis*, (ii) the identification of FAs as PTP1B and α-glucosidase inhibitors, (iii) the demonstration that sterols derived from *H. fusiformis* function as PTP1B inhibitors, and (iv) the demonstration that glycyrrhizin and its metabolites function as PTP1B and α-glucosidase inhibitors. Notably, glycyrrhizin (**18**) is metabolized by β-d-glucuronidase or intestinal flora to glycyrrhetinic acid [44,45]. Therefore, the in vivo anti-diabetic activity of **18** may be attributed to the PTP1B and α-glucosidase inhibitory activity of its metabolite, glycyrrhetinic acid.

In conclusion, the in vitro experimental and in silico computational results from this study confirmed that compounds isolated from *H. fusiformis* exhibit potent PTP1B and α-glucosidase inhibitory activity. Among the isolated compounds, FAs and triterpenoid derivatives showed potent inhibitory activity against both enzymes. However, sterols did not show any inhibition activity against α-glucosidase. Taken together, these results suggest that constituents of *H. fusiformis* could be used as promising anti-diabetic materials to delay the absorption of glucose via inhibition of α-glucosidase enzyme in the digestive organs and to enhance the insulin signaling pathway via inhibition of the PTP1B enzyme in insulin-sensitive organs.

## 4. Materials and Methods 

### 4.1. General Experimental Procedures

The specific rotations were operated on a JASCO DIP-370 digital polarimeter. The ^1^H- and ^13^C-NMR spectra were recorded in methanol-*d_4_* and chloroform-*d* on a JEOL JNM ECP-400 spectrometer (Tokyo, Japan) at 400 MHz and 100 MHz, respectively. The infrared (IR) spectra were measured on a Mattson Polaris FT/IR-300E spectrophotometer. Mass spectra were recorded using a Quattro II mass spectrometer. Column chromatography was conducted using Diaion HP-20, Sephadex LH-20 (20–100 µM, Sigma, St. Louis, MO, USA), silica (Si) gel 60 (70–230 mesh, Merck, Darmstadt, Germany), and LiChroprep RP-18 (40–63 µM, Merck). All TLC was performed on precoated Merck Kiesel gel 60 F_254_ plates (20 × 20 cm, 0.25 mm) and RP-18 F_254S_ plates (5 × 10 cm, Merck). The spray reagent was 25% H_2_SO_4_.

### 4.2. Chemicals and Reagents

Yeast α-glucosidase, *p*-nitrophenyl α-d-glucopyranoside (*p*NPG), acarbose, *p*-nitrophenyl phosphate (*p*NPP), ursolic acid, ethylenediaminetetraacetic acid (EDTA), 18α-glycyrrhetinic acid, and 18β-glycyrrhetinic acid were purchased from Sigma Aldrich. A truncated form of human recombinant PTP1B (amino acid 1-322) was purchased from Enzo Life Sciences (Farmingdale, NY, USA) and dithiothreitol (DTT) was purchased from Bio-Rad Laboratories (Hercules, CA, USA). All other chemicals and solvents were purchased from E. Merck, Fluka, and Sigma-Aldrich, unless otherwise stated.

### 4.3. Plant Material

Seaweed *H. fusiformis* was purchased from Wando, Republic of Korea. A whole plant voucher specimen was registered and deposited at the Department of Food and Life Science, Pukyong National University, Busan, South Korea (Professor Jae Sue Choi).

### 4.4. Extraction, Fractionation, and Isolation

The *H. Fusiformis* plant (25 kg) was extracted with 95% MeOH (10 L × 3 times) for 3 h at 70 °C. Then, the total filtrate was concentrated to dryness *in vacuo* at 70 °C to give a MeOH extract. The MeOH extract (4.8 kg) was suspended in water (5 L) and subjected to Diaion HP-20 column chromatography (CC) eluted with solvent systems of MeOH:H_2_O (0:1, 1:3, 1:1, 3:1, 1:0) and acetone (100%) to give seven fractions (HF-A–F). Fraction HF-F (40 g) was subjected to SiO_2_ CC eluted with the solvent system of *n*-hexane-acetone gradient (1:0 to 0:1) to afford 16 sub-fractions (HF-1–16). Sub-fraction HF-1 (3.6 g) was chromatographed on SiO_2_ CC with a mobile phase gradient of *n*-hexane:CH_2_Cl_2_:EtOAc (H:C:E, 6:2:1–5:5:5) to give (*Z*)-hexadec-12-enoic acid (**1**) (6.4 mg) and palmitic acid (2) (31 mg) [46]. The last fraction of HF-1 (980 mg) was chromatographed over a RP C18 column eluted with MeOH:H_2_O (8:1) to give compound **2** (61 mg) and (*Z*)-octaec-9-enoic acid (**3**) (20 mg) [23]. Fraction HF-2 (3.1 g) was subjected to SiO_2_ open CC eluted with H:C:E gradient (10:1:1 to 1:1:1) to give six sub-fractions (HF-2A–2F). Sub-fractions HF-2B (420 mg) and HF-2C (735 mg) were chromatographed over open column SiO_2_ with a solvent system of H:C:E (4:4:1) to yield a mixture of 24*R* and 24*S*-saringosterol (**4**) (70 mg) and fucosterol (**5**) (200 mg), respectively [25,26]. Sub-fraction HF-2E (345 mg) was chromatographed over a RP C18 open column eluted with MeOH:H_2_O (6:1) to yield mixture of 24*R*,28*R* and 24*S*,28*R*-epoxy-24-ethylcholesterol (**6**) (6.2 mg) and (8*Z*,11*Z*,14*Z*)-heptadeca-8,11,14-trienoic acid (**7**) (90 mg) [23]. Sub-fraction HF-2F (980 mg) was subjected to RP C18 open CC to give (7*Z*,10*Z*,13*Z*)-octadeca-7,10,13-trienoic acid (**8**) (19 mg) and (7*Z*,9*Z*,11*Z*,13*Z*)-icosa-7,9,11,13-tetraenoic acid (**9**) (61 mg). Fraction HF-3 (1.2 g) was chromatographed to open column SiO_2_ using a solvent system of H:C:E (1:4:0.5–1:4:5) to afford six fractions (HF-3A–3F). Sub-fraction HF-3C (512 mg) was further chromatographed over a RP C18 open column eluted with a solvent system acetonitrile:MeOH:H_2_O (A:M:H, 4:4:1) to yield compounds (6*Z*,9*Z*,12*Z*,15*Z*)-octadeca-6,9,12,15-tetraenoic acid (**10**) (14 mg) and (5*Z*,8*Z*,11*Z*,14*Z*,17*Z*)-eicosa-5,8,11,14,17-pentaenoic acid (**11**) (50 mg) [23]. Sub-fraction HF-8 (307 mg) was subjected to SiO_2_ CC eluted with solvent systems of H:C:E (4:4:0.5 to 4:4:4) to give four sub-fractions (HF-8A–8D). Sub-fraction HF-8C (41 mg) was purified by RP C18 open column using a solvent system A:M:H (5:4:1) to afford (8*Z*,11*Z*,14*Z*)-heptadeca-8,11,14-trienoic acid (**12**) (10 mg). Sub-fraction HF-8B (89 mg) was chromatographed over RP C18 open column eluted with A:M:H (4:4:2) to yield 2-(7′-(2″-hydroxy-3″-((5*Z*,8*Z*,11*Z*)-icosatrienoyloxy)propoxy)-7′-oxoheptanoyl) oxymethylpropenoic acid (**13**) (25 mg). Fraction HF-15 (1.32 g) was subjected to RP C18 open CC eluted with a solvent system of acetone:H_2_O (1:2) to give four fractions (HF-15A–15D). Sub-fractions HF-15A (48 mg) and HF-15B (56 mg) were purified by a RP C18 open column using mobile phase acetone:H_2_O (1:3) to yield cedrusin (**14**) (3.0 mg) and 1-(4-hydroxy-3-methoxyphenyl)-2 -[2-hydroxy-4-(3-hydroxypropyl)phenoxy]-1,3-propanediol (**15**) (5.5 mg), respectively [27,28]. Sub-fraction HF-15C (206 mg) was chromatographed over a RP C18 open CC eluted with acetone:H_2_O (1:3) to give benzyl alcohol alloside (**16**) (7.8 mg) and madhusic acid A (**17**) (5.0 mg) [29,30]. Fraction HF-16 (623 mg) was chromatographed over a RP C18 open column using solvent systems of MeOH:H_2_O (1:5–1:1) to give four fractions (HF-16A–16D). Sub-fraction HF-16A (41 mg) was purified over a RP C18 open column using a solvent system MeOH:H_2_O (1:6) to yield 18β-glycyrrhetinic acid-3-*O*-*β*-d-glucuronopyranosyl-1(→2)-*β*-d-glucuronide (**18**, glycyrrhizin) (5.1 mg) [31]. Sub-fractions HF-16B (73 mg) and HF-16C (38 mg) were chromatographed by RP C18 open CC using mobile phase A:M:H (1:1:10) to give 18β-glycyrrhetinic acid-3-*O*-*β*-d-glucuronopyranosyl- 1(→2)-*β*-d-glucuronide-6’-methyl ester (**19**) (2.5 mg) and (3*R*)-4-[(2*R*,4*S*)-4-acetoxy-2-hydroxy- 2,6,6-trimethylcyclohexylidene]but-3-en -2-one (**20**, apo-9′-fucoxanthinone) (1.6 mg), respectively [32,33]. Sub-fraction HF-16D (463 mg) was chromatographed by RP C18 open CC to give tyramine (**21**) (2.9 mg) [34]. By comparison with previously published data, the isolated compounds **1**–**21** were identified by GC-MS and ^1^H- and ^13^C-NMR analysis. The chemical structures of the isolated compounds are shown in Figure 1. In the Table S1, molecular weight and molecular formulas of all the isolated compounds were mentioned.

Compound **13**: Yellow syrup; ${\left[\alpha \right]}_{D}^{23}$ −50.55° (*c* 0.1, MeOH); IR (KBr, ν_max_, cm^−1^): 3705.55, 3680.48, 3651.07 (O-H stretching), 3005.52-3022.39 (C-H olefins), 2957.79-2923.07-2892.7-2852.69 (aliphatic C-H stretching), 1737.07 (C=O stretching), 1055.35-1033.18-1011.96 (C-O stretching); HR-ESI-MS: *m/z* 607.3820 [M + H]^+^ (calcd. for C_34_H_55_O_9_, 607.3846). ^1^H-NMR (400 MHz in CDCl_3_): 6.42 (1H, s, H4a), 5.96 (1H, s, H4b), 5.36 (6H, overlapped, H5‴, H6‴, H8‴, H9‴, H11‴, H12‴), 5.24 (1H, m, H2″), 4.36 (1H, dd, *J* = 3.7 and 12 Hz, H1″), 4.20 (2H, overlapped, H3), 4.16 (1H, overlapped, 3″), 4.14 (1H, overlapped, H1″), 3.64 (1H, d, *J* = 5.38 Hz, H3″), 2.79 (4H, overlapped, H7‴, H10‴), 2.31 (6H, t, *J* = 7.84 Hz, H2′, H6′, H2‴), 2.06 (4H, overlapped, H4‴, H13‴), 1.59 (2H, m, H3‴), 1.25 (16H, overlapped, H3′-5′, H14‴-17‴), 0.88 (3H, t, *J* = 6.74 Hz, H20‴); ^13^C-NMR (100 MHz in CDCl_3_): 173.6 (C1′ and C7′), 173.2 (C1‴), 170.2 (C1), 136.3 (C2), 130.4, (C12‴), 130.1 (C6‴, C11‴), 129.8 (C9‴), 128.3 (C8‴), 128.2 (C5‴), 70.1 (C2″), 69.3 (C3″, C3), 62.7 (C1″), 34.4 (C2‴), 34.3 (C2′, C6′), 32.1 (C18‴), 29.8 (C14‴, C15‴), 29.7 (C5′), 29.6 (C16‴), 29.5 (C18‴), 29.4 (C8‴), 29.3 (C17‴), 29.2 (C4′), 27.4 (C4‴), 27.3 (C13‴), 25.8 (C7‴, C10‴), 25.1 (C3′, C3‴), 25.0 (C3′, C5′), 22.8 (C19‴), 14.2 (C20‴, CH_3_). See Figure 2 for COSY and HMBC correlation.

Compound **15**: Yellowish powder; ^1^H-NMR (400 MHz in CD_3_OD): 7.00 (1H, d, *J* = 1.36 Hz, H2′), 6.88 (1H, d, *J* = 1.4 Hz, H5′), 6.87 (1H, s, H6″), 6.83 (1H, s, H5′), 6.75 (1H, s, H3′), 6.70 (1H, d, *J* = 8.23 Hz, H5″), 4.85 (1H, s, H1), 4.00 (1H, m, H2), 3.87 (3H, s, O-CH_3_, H7″), 3.67 (1H, dd, *J* = 2.29 and 12.24 Hz, H3a), 3.55 (2H, t, *J* = 6.48 Hz, H9″), 3.46 (1H, dd, *J* = 4.57 and 12.31 Hz, H3b), 2.58 (2H, t, *J* = 8 Hz, 2H, H7″), 1.79 (2H, dt, *J* = 6 and 14 Hz, H8″); ^13^C-NMR (100 MHz in CD_3_OD): 149.2 (C3′), 148.4 (C4′), 145.0 (C2″), 142.9 (C1″), 136.4 (C4″), 129.6 (C1′), 126.4 (C5′), 122.4 (C5″), 121.6 (C6′), 117.6 (C3″ and C6″), 111.9 (C2′), 80.0 (C2), 77.8 (C1), 62.2 (C3 and C9″), 56.4 (C7′, O-CH_3_) 35.6 (C8″), 32.4 (C7″).

### 4.5. In Vitro α-Glucosidase Inhibitory Activity Assay

Enzyme inhibition studies were carried out spectrophotometrically in a 96-well micro-plate reader using a procedure reported by Li et al. [47]. Acarbose was used as a positive control. 

### 4.6. In Vitro PTP1B Inhibitory Activity Assay

The inhibitory activity of isolated compounds against truncated form of human recombinant PTP1B was evaluated using *p*NPP as a substrate [48]. The amount of *p*-nitrophenyl produced after enzymatic dephosphorylation of *p*NPP was estimated by measuring the absorbance at 405 nm using a micro-plate spectrophotometer (Molecular Devices, Sunnyvale, CA, USA). Ursolic acid was used as a positive control. 

### 4.7. Kinetic Parameters of Active Compounds towards PTP1B Inhibition

The inhibition constant (*K_i_*) and inhibition mode for the inhibition of PTP1B was calculated by the Lineweaver-Burk plot and its secondary plot of the slope and the *y*-intercept of compounds [49,50]. The kinetic parameters were obtained over various concentrations of substrate (0 to 2 mM) and inhibitors (0, 4.7, 23.3, and 116.6 µM for compound **6**; 0, 2.5, 5, and 10 µM for compound **13**; 0, 5, 10, 20, and 40 µM for compounds **22** and **23**). Graphs were generated using SigmaPlot 12.0 (Systat Software Inc., San Jose, CA, USA).

### 4.8. PTP1B Molecular Docking Simulations

For docking studies, the crystal structure of the truncated form of PTP1B protein target (amino acid 1-282) was obtained from the RCSB Protein Data Bank (PDB) with the accession code 1T49 [42]. The co-crystallized ligand, 3-(3,5-dibromo-4-hydroxy-benzoyl)-2-ethyl- benzofuran-6-sulfonic acid (4-sulfamoyl-phenyl)-amide (compound B), was used to generate the grid box for allosteric inhibition mode, whereas the reported catalytic ligand, 3-({5-[(*N*-acetyl-3-{4-[(carboxycarbonyl)(2-carboxyphenyl)amino] -1-naphthyl}-L-alanyl)amino] pentyl}oxy)-2-naphthoic acid (compound A) (PDB ID: 1NNY), was used to generate the grid box for catalytic inhibition mode. The 3D structures of **5**, **22**, and **23** were downloaded from PubChem Compound (NCBI) with compound CIDs of 5281328, 12193680 and 10114, respectively. The 3D structures of 24*R*,28*R* epoxy-24-ethylcholesterol (**6a**), 24*S*,28*R*-epoxy-24-ethylcholesterol (**6b**), and **13** were generated by Chem3D pro (v12.0, Cambridge Soft Corporation, Cambridge, MA, USA). The structures of ligands were adjusted to neutral (pH 7.0) using MarvinSketch (ChemAxon, Budapest, Hungary) and minimized using Chem3D pro. The results were visualized and analyzed using UCSF Chimera (v1.13.1, http://www.cgl.ucsf.edu/chimera/), Discovery Studio (v16.1, Accelrys, San Diego, CA, USA), and Ligplot^+^ (v1.4.5, European Bioinformatics Institute, London, England).

### 4.9. Statistical Analysis

All experiments were carried out in triplicate and repeated on three separate days. All data are expressed as the mean ± standard deviation (SD) (*n* = 3).

## Figures and Tables

**Table 1:** PTP1B and α-glucosidase inhibitory activity of compounds isolated from *Hizikia fusiformis*.

Compounds	IC_50_ (μM) ^a^		Enzyme Kinetics	
	PTP1B	α-Glucosidase	*K*_i_ (μM) ^b^	Inhibition Type ^c^
**1**	6.59 ± 0.09	48.05 ± 3.37	‒	‒
**2**	49.39 ± 1.39	93.63 ± 3.68	‒	‒
**3**	13.65 ± 0.49	113.44 ± 2.47	‒	‒
**4**	> 150	> 150	‒	‒
**5**	50.58 ± 1.86	> 150	‒	‒
**6**	16.70 ± 0.36	> 150	24.43	Non-competitive
**7**	4.86 ± 1.36	> 200	‒	‒
**8**	13.58 ± 0.10	111.51 ± 1.44	‒	‒
**9**	10.68 ± 0.17	34.85 ± 2.39	‒	‒
**10**	16.43 ± 0.07	> 200	‒	‒
**11**	*NT*	*NT*	‒	‒
**12**	11.51 ± 0.52	43.90 ± 0.77	‒	‒
**13**	4.92 ± 0.01	> 150	4.13	Non-competitive
**14**	174.19 ± 5.44	133.84 ± 3.86	‒	‒
**15**	>400	> 250	‒	‒
**16**	>400	> 250	‒	‒
**17**	>400	> 300	‒	‒
**18**	110.33 ± 0.39	> 150	‒	‒
**19**	49.39 ± 1.39	> 150	‒	‒
**20**	323.21 ± 0.84	> 250	‒	‒
**21**	188.06 ± 3.21	273.23 ± 5.65	‒	‒
**22**	10.40 ± 0.75	113.30 ± 0.70	3.17	Competitive
**23**	26.07 ± 0.59	128.72 ± 3.88	16.23	Competitive
Ursolic acid ^d^	7.31 ± 0.16	‒	‒	‒
Acarbose ^d^	‒	158.41 ± 1.05	‒	‒

^a^ The IC_50_ values (μM) were calculated from a log dose inhibition curve and are expressed as mean ± SD of triplicate experiments. ^b^ PTP1B inhibition constants (μM) of tested compounds determined using secondary plot of the *slopes* and *y-intercept* of each linear regression of Lineweaver-Burk plot ^c^ PTP1B inhibition types of tested compounds determined using Lineweaver–Burk plots. ^d^ Positive controls. *NT* Not tested due to low solubility in 10% dimethyl sulfoxide (DMSO). *(‒)* Not tested.

**Table 2:** Molecular interaction residues and binding energy (B-Score) of compounds from *Hizikia fusiformis* as well as reported inhibitors against PTP1B (PDB ID: 1T49).

Compounds	B-Score (kcal/mol)	H-Bonds Interacting Residues	Hydrophobic Interacting Residues
**5**	−8.10	Glu200	Leu192, Asn193, Glu276, Gly277, Lys279, Phe196, Phe280
**6a** (24*R* and 28*R*)	−7.90	Glu200	Pro188, Ala189, Leu192, Asn193, Glu276, Phe196, Phe280
**6b** (24*S* and 28*R*)	−8.66	Glu200	Pro188, Ala189, Leu192, Asn193, Phe196, Glu276, Gly277, Lys279, Phe280
**13**	−5.03	Lys150, Tyr153, Asn193, Lys197	Lys150, Ser151, Tyr152, Tyr153, Ala189, Pro188, Leu272, Ser187, Glu276, Leu192, Asn193, Lys197, Phe280
**22**	−9.09	Lys116, Lys120 (Salt bridge)	Lys120, Phe182, Gly183, Asp265, Thr263, Glu115, Arg221
**23**	−8.81	Asp48, Lys116 (Salt bridge), Gly183	Tyr46, Val49, Ala217, Gln262, Phe182
Standard A ^a^	−11.23	Arg24, Tyr46, Asp48, Ser216, Ala217, Arg221, Arg254, Gln262	Ser28, Val49, Lys116, Lys120, Cys215, Ile219, Gly220, Met258, Gly259
Standard B ^a^	−10.98	Asn193, Glu276	Ala189, Leu192, Phe196, Gly277, Lys279, Phe280, Ile281, Met282

^a^ Standard A (3-({5-[(*N*-acetyl-3-{4-[(carboxycarbonyl)(2-carboxyphenyl)amino]-1-naphthyl}-L-alanyl)amino] pentyl}oxy)-2-naphthoic acid) and B (3-(3,5-dibromo-4-hydroxy-benzoyl)-2-ethyl-benzofuran-6-sulfonic acid (4-sulfamoyl-phenyl)-amide) are positive catalytic and allosteric compounds for docking simulation, respectively.

## Supplementary Materials

The following are available online at https://www.mdpi.com/1660-3397/17/5/302/s1, Figure S1: ^13^C (100MHz in CDCl_3_)- and ^1^H (400MHz in CDCl_3_)-NMR spectrum of compound **13**, Figure S2: HMBC-NMR spectrum of compound **13**, Figure S3: COSY-NMR spectrum of compound **13**, Figure S4: HSQC-NMR spectrum of compound **13**, Figure S5: HR-ESI-MS data of compound **13**, Figure S6: FT-IR spectrum of compound **13**. Figure S7: ^13^C (100MHz in CDCl_3_)- and ^1^H (400MHz in CDCl_3_)-NMR spectrum of compound **1**. Figure S8: EI-MS data of compound **1**. Figure S9: ^13^C (100MHz in CDCl_3_)- and ^1^H (400MHz in CDCl_3_)-NMR spectrum of compound **2**. Figure S10: EI-MS data of compound **2**. Figure S11: ^13^C (100MHz in CDCl_3_)- and ^1^H (400MHz in CDCl_3_)-NMR spectrum of compound **3**. Figure S12: EI-MS data of compound **3**. Figure S13: ^13^C (100MHz in CDCl_3_)- and ^1^H (400MHz in CDCl_3_)-NMR spectrum of compound **4**. Figure S14: ^13^C (100MHz in CDCl_3_)- and ^1^H (400MHz in CDCl_3_)-NMR spectrum of compound **5**. Figure S15: ^13^C (100MHz in CDCl_3_)- and ^1^H (400MHz in CDCl_3_)-NMR spectrum of compound **6**. Figure S16: ^13^C (100MHz in CDCl_3_)- and ^1^H (400MHz in CDCl_3_)-NMR spectrum of compound **7**. Figure S17: EI-MS data of compound **7**. Figure S18: ^13^C (100MHz in CDCl_3_)- and ^1^H (400MHz in CDCl_3_)-NMR spectrum of compound **8**. Figure S19: EI-MS data of compound **8**. Figure S20: ^13^C (100MHz in CDCl_3_)- and ^1^H (400MHz in CDCl_3_)-NMR spectrum of compound 9. Figure S21: EI-MS data of compound **9**. Figure S22: ^13^C (100MHz in CDCl_3_)- and ^1^H (400MHz in CDCl_3_)-NMR spectrum of compound **10**. Figure S23: EI-MS data of compound **10**. Figure S24: ^13^C (100MHz in CDCl_3_)- and ^1^H (400MHz in CDCl_3_)-NMR spectrum of compound **11**. Figure S25: EI-MS data of compound **11**. Figure S26: ^13^C (100MHz in CDCl_3_)- and ^1^H (400MHz in CDCl_3_)-NMR spectrum of compound **12**. Figure S27: EI-MS data of compound **12**. Figure S28: ^13^C (100MHz in CD_3_OD)- and ^1^H (400MHz in CD_3_OD)-NMR spectrum of compound **14**. Figure S19: ^13^C (100MHz in CD_3_OD)- and ^1^H (400MHz in CD_3_OD)-NMR spectrum of compound **15**. Figure S30: ^13^C (100MHz in CD_3_OD)- and ^1^H (400MHz in CD_3_OD)-NMR spectrum of compound **16**. Figure S31: ^13^C (100MHz in CD_3_OD)- and ^1^H (400MHz in CD_3_OD)-NMR spectrum of compound **17**. Figure S32: ^13^C (100MHz in CD_3_OD)- and ^1^H (400MHz in CD_3_OD)-NMR spectrum of compound **18**. Figure S33: ^13^C (100MHz in CD_3_OD)- and ^1^H (400MHz in CD_3_OD)-NMR spectrum of compound **19**. Figure S34: ^13^C (100MHz in CD_3_OD)- and ^1^H (400MHz in CD_3_OD)-NMR spectrum of compound **20**. Figure S35: ^13^C (100MHz in CD_3_OD)- and ^1^H (400MHz in CD_3_OD)-NMR spectrum of compound **21**. Table S1. Molecular weight and molecular formula of isolated compounds.
