# Supplementary material for: Experimental and Computational Study to Reveal the Potential of Non-Polar Constituents from Hizikia fusiformis as Dual Protein Tyrosine Phosphatase 1B and α-Glucosidase Inhibitors
Source: Mar Drugs. 2019 May 22;17(5):302. doi: 10.3390/md17050302 (PMC6562952; doi:10.3390/md17050302)
Supplement: Supplementary file 1 [file marinedrugs-17-00302-s001.pdf]

Supplementary Files:

Experimental and Computational Study to Reveal the Potential of Non-Polar Constituents from *Hizikia fusiformis* as Dual Protein Tyrosine Phosphatase 1B and α-Glucosidase Inhibitors

Su Hui Seong [1,†], Duc Hung Nguyen [2,†], Aditi Wagle [1], Mi Hee Woo [2,*], Hyun Ah Jung [3,*] and Jae Sue Choi [1,*]

[1] Department of Food and Life Science, Pukyong National University, Busan 48513, Republic of Korea; seongsuhui@naver.com (S.H.S.), aditiwagle05@gmail.com (A.W.)

[2] College of Pharmacy, Drug Research and Development Center, Catholic University of Daegu, Gyeongsan 38430, Republic of Korea; duchung1982fushico@gmail.com (D.H.N.)

[3] Department of Food Science and Human Nutrition, Chonbuk National University, Jeonju 54896, Republic of Korea

* Correspondence: woomh@cu.ac.kr (M.H.W.); jungha@jbnu.ac.kr (H.A.J.); choijs@pknu.ac.kr (J.S.C.); Tel.: +82-53-850-3620 (M.H.W); +82-63-270-4882 (H.A.J.); +82-51-629-5845 (J.S.C.)

† These authors contributed equally to this work.

Contents

Figure S1. ^{13}C (100 MHz in $CDCl_3$)- and ^{1}H (400 MHz in $CDCl_3$)-NMR spectrum of compound **13**.

Figure S2. HMBC-NMR spectrum of compound **13**.

Figure S3. COSY-NMR spectrum of compound **13**.

Figure S4. HSQC-NMR spectrum of compound **13**.

Figure S5. HR-ESI-MS data of compound **13**.

Figure S6. FT-IR spectrum of compound **13**

Figure S7. ^{13}C (100 MHz in $CDCl_3$)- and ^{1}H (400 MHz in $CDCl_3$)-NMR spectrum of compound **1**.

Figure S8. EI-MS data of compound **1**.

Figure S9. ^{13}C (100 MHz in $CDCl_3$)- and ^{1}H (400 MHz in $CDCl_3$)-NMR spectrum of compound **2**.

Figure S10. EI-MS data of compound **2**.

Figure S11. ^{13}C (100 MHz in $CDCl_3$)- and ^{1}H (400 MHz in $CDCl_3$)-NMR spectrum of compound **3**.

Figure S12. EI-MS data of compound **3**.

Figure S13. ^{13}C (100 MHz in $CDCl_3$)- and ^{1}H (400 MHz in $CDCl_3$)-NMR spectrum of compound **4**.

Figure S14. ^{13}C (100 MHz in $CDCl_3$)- and ^{1}H (400 MHz in $CDCl_3$)-NMR spectrum of compound **5**.

Figure S15. ^{13}C (100 MHz in $CDCl_3$)- and ^{1}H (400 MHz in $CDCl_3$)-NMR spectrum of compound **6**.

Figure S16. ^{13}C (100 MHz in $CDCl_3$)- and ^{1}H (400 MHz in $CDCl_3$)-NMR spectrum of compound **7**.

Figure S17. EI-MS data of compound **7**.

Figure S18. ^{13}C (100 MHz in $CDCl_3$)- and ^{1}H (400 MHz in $CDCl_3$)-NMR spectrum of compound **8**.

Figure S19. EI-MS data of compound **8**.

Figure S20. ^{13}C (100 MHz in $CDCl_3$)- and ^{1}H (400 MHz in $CDCl_3$)-NMR spectrum of

compound 9.

Figure S21. EI-MS data of compound **9**.

Figure S22. ^{13}C (100 MHz in $CDCl_3$)- and ^{1}H (400 MHz in $CDCl_3$)-NMR spectrum of compound **10**.

Figure S23. EI-MS data of compound **10**.

Figure S24. ^{13}C (100 MHz in $CDCl_3$)- and ^{1}H (400 MHz in $CDCl_3$)-NMR spectrum of compound **11**.

Figure S25. EI-MS data of compound **11**.

Figure S26. ^{13}C (100 MHz in $CDCl_3$)- and ^{1}H (400 MHz in $CDCl_3$)-NMR spectrum of compound **12**.

Figure S27. EI-MS data of compound **12**.

Figure S28. ^{13}C (100 MHz in CD_3OD)- and ^{1}H (400 MHz in CD_3OD)-NMR spectrum of compound **14**.

Figure S29. ^{13}C (100 MHz in CD_3OD)- and ^{1}H (400 MHz in CD_3OD)-NMR spectrum of compound **15**.

Figure S30. ^{13}C (100 MHz in CD_3OD)- and ^{1}H (400 MHz in CD_3OD)-NMR spectrum of compound **16**.

Figure S31. ^{13}C (100 MHz in CD_3OD)- and ^{1}H (400 MHz in CD_3OD)-NMR spectrum of compound **17**.

Figure S32. ^{13}C (100 MHz in CD_3OD)- and ^{1}H (400 MHz in CD_3OD)-NMR spectrum of compound **18**.

Figure S33. ^{13}C (100 MHz in CD_3OD)- and ^{1}H (400 MHz in CD_3OD)-NMR spectrum of compound **19**.

Figure S34. ^{13}C (100 MHz in CD_3OD)- and ^{1}H (400 MHz in CD_3OD)-NMR spectrum of compound **20**.

Figure S35. ^{13}C (100 MHz in CD_3OD)- and ^{1}H (400 MHz in CD_3OD)-NMR spectrum of compound **21**.

Table S1. Molecular weight and molecular formula of isolated compounds.

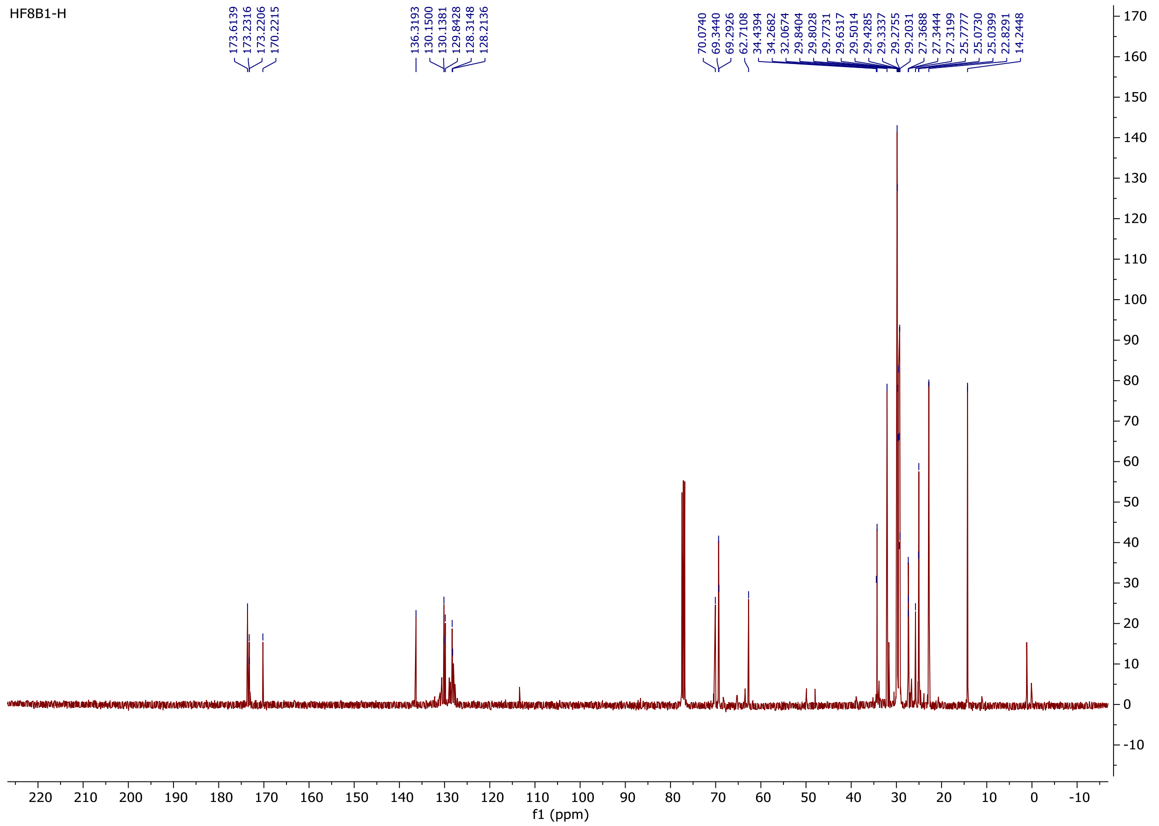

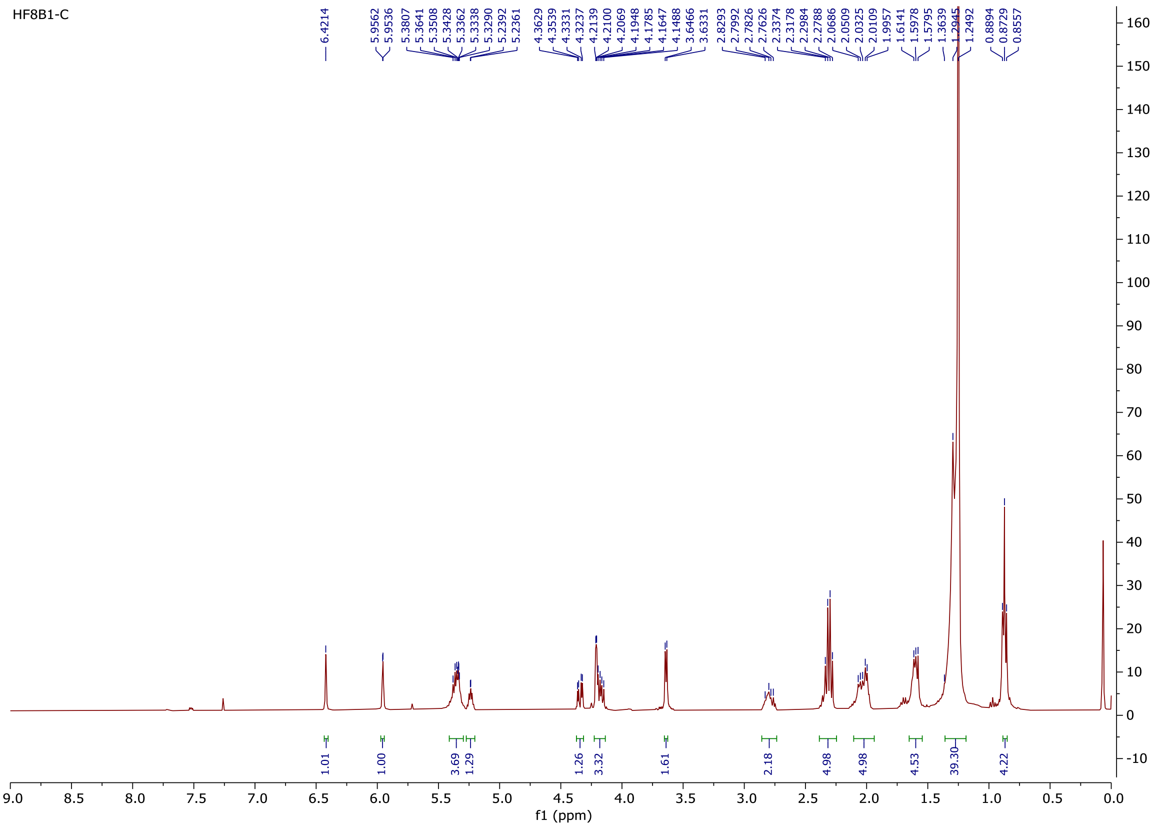

Figure S1. ^{13}C (100 MHz in $CDCl_3$)- and ^{1}H (400 MHz in $CDCl_3$)-NMR spectrum of compound **13**.

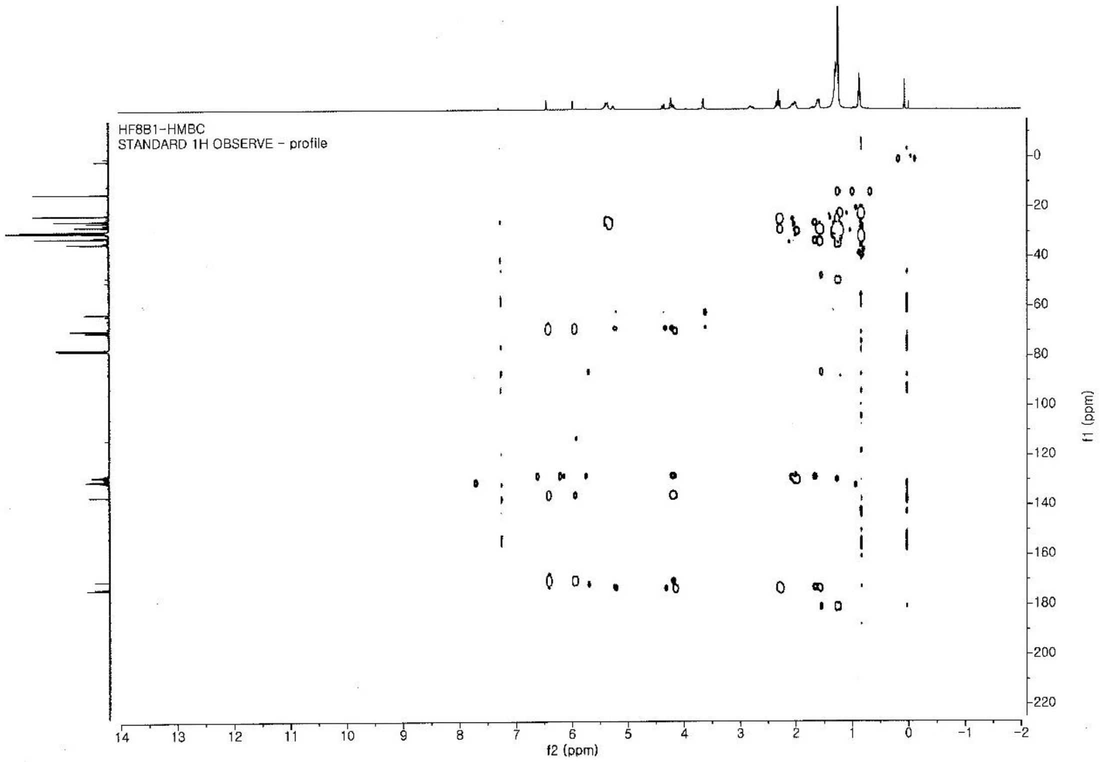

Figure S2. HMBC-NMR spectrum of compound **13**.

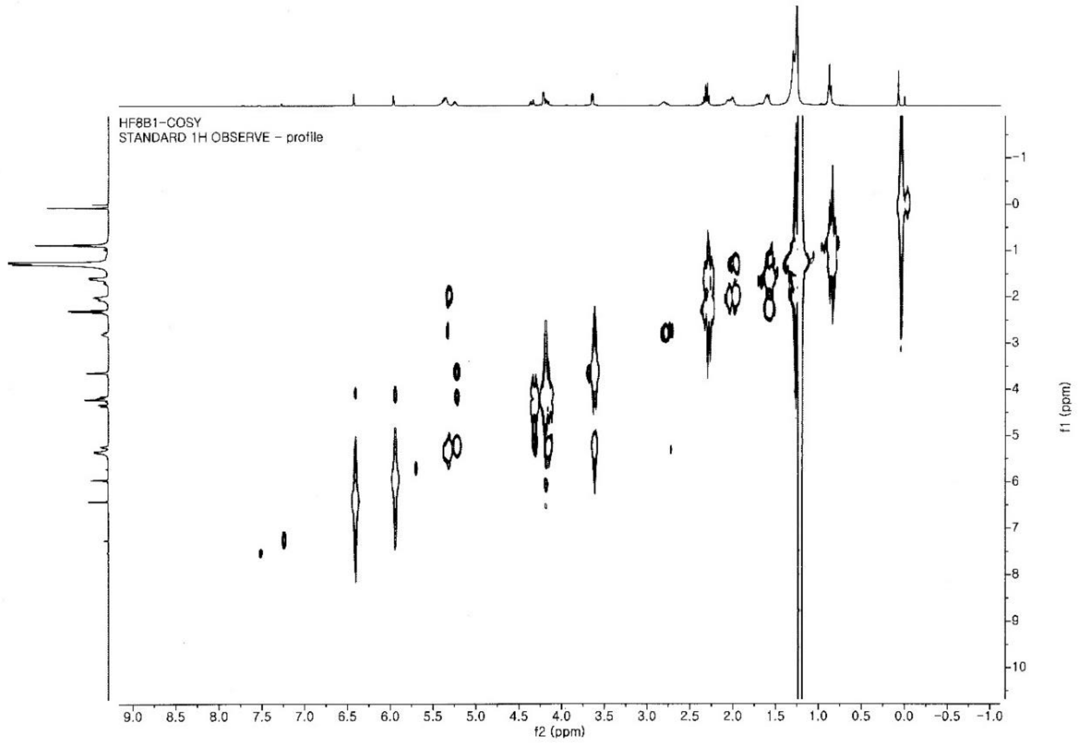

Figure S3. COSY-NMR spectrum of compound **13**.

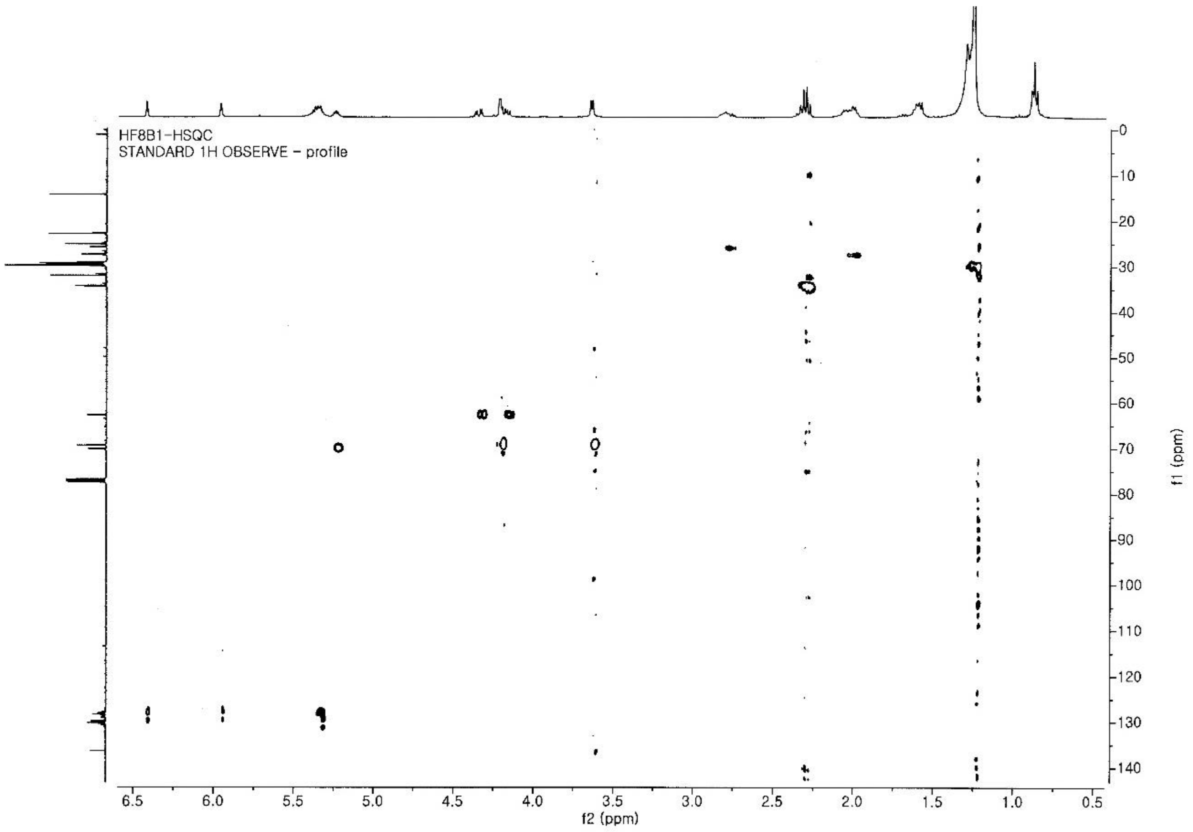

Figure S4. HSQC-NMR spectrum of compound **13**.

Display Report

Analysis Info

Analysis Name	D:\Data\ESI\2016\0531\HF8B1.d	Acquisition Date	5/31/2016 9:33:03 AM
Method	Na formate_50_1000mz_Pos.m	Operator	BDAL@DE
Sample Name	HF8B1	Instrument	maxis HD 1820881.21289
Comment			

Acquisition Parameter

Source Type	ESI	Ion Polarity	Positive	Set Nebulizer	0.4 Bar
Focus	Active	Set Capillary	4500 V	Set Dry Heater	200 °C
Scan Begin	50 m/z	Set End Plate Offset	-500 V	Set Dry Gas	4.0 l/min
Scan End	1000 m/z	Set Charging Voltage	2000 V	Set Divert Valve	Waste
		Set Corona	4000 nA	Set APCI Heater	200 °C

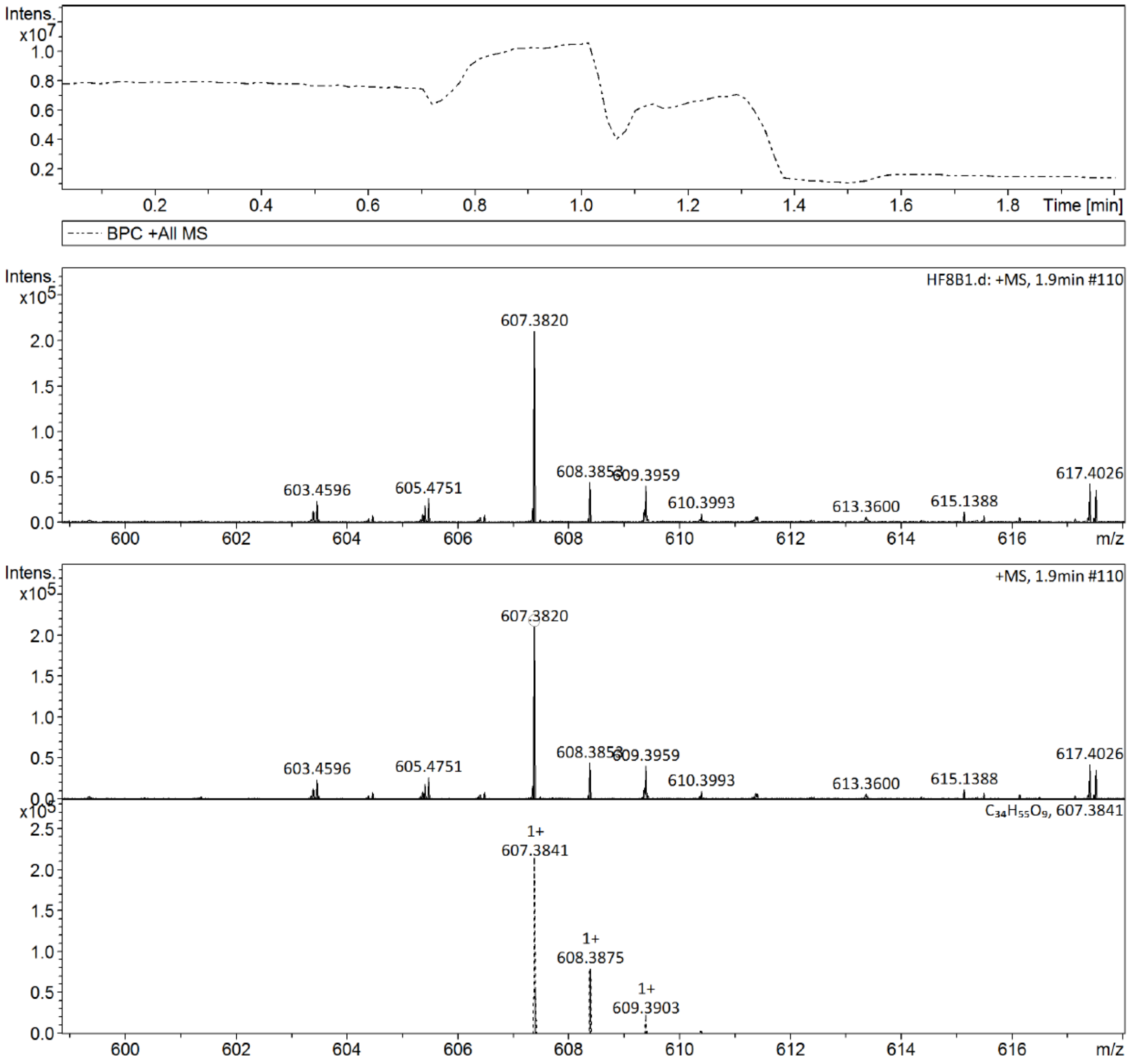

Figure S5. HR-ESI-MS data of compound **13**.

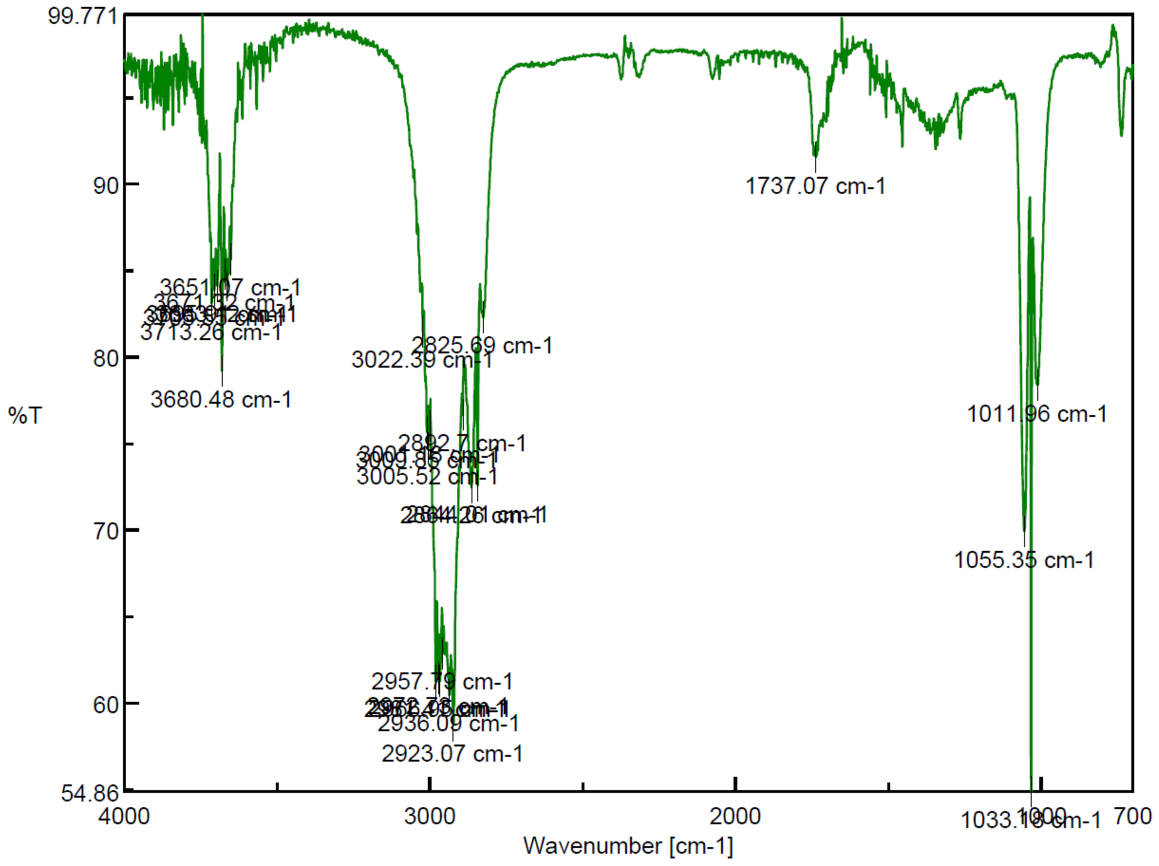

Figure S6. FT-IR spectrum of compound **13**.

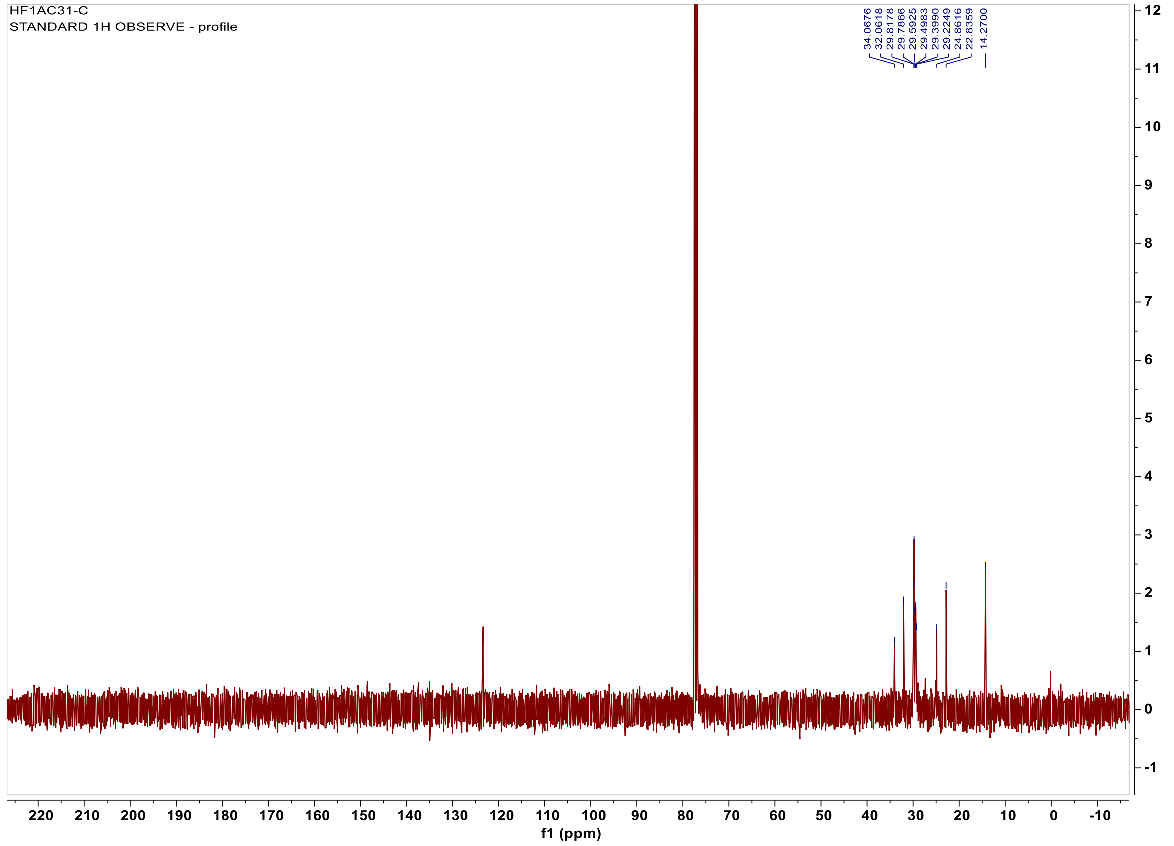

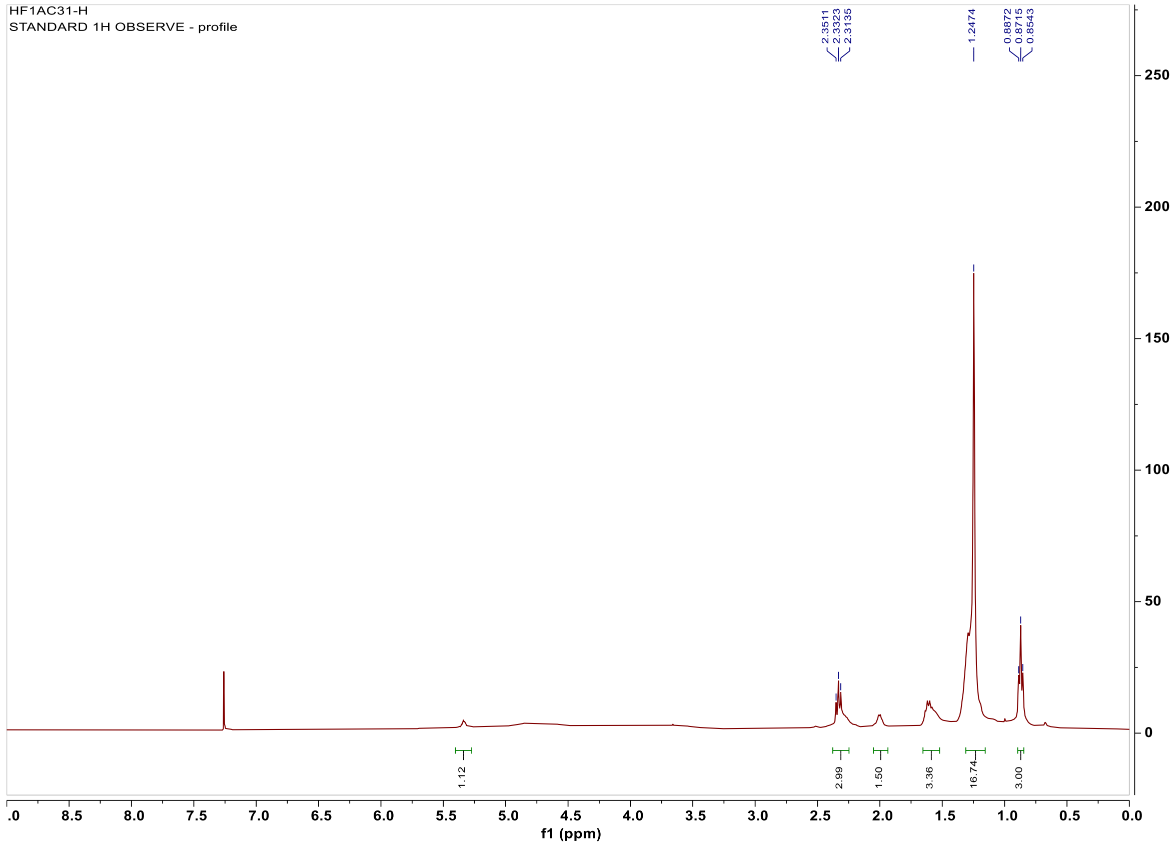

Figure S7. ^{13}C (100 MHz in $CDCl_3$)- and ^{1}H (400 MHz in $CDCl_3$)-NMR spectrum of compound **1**.

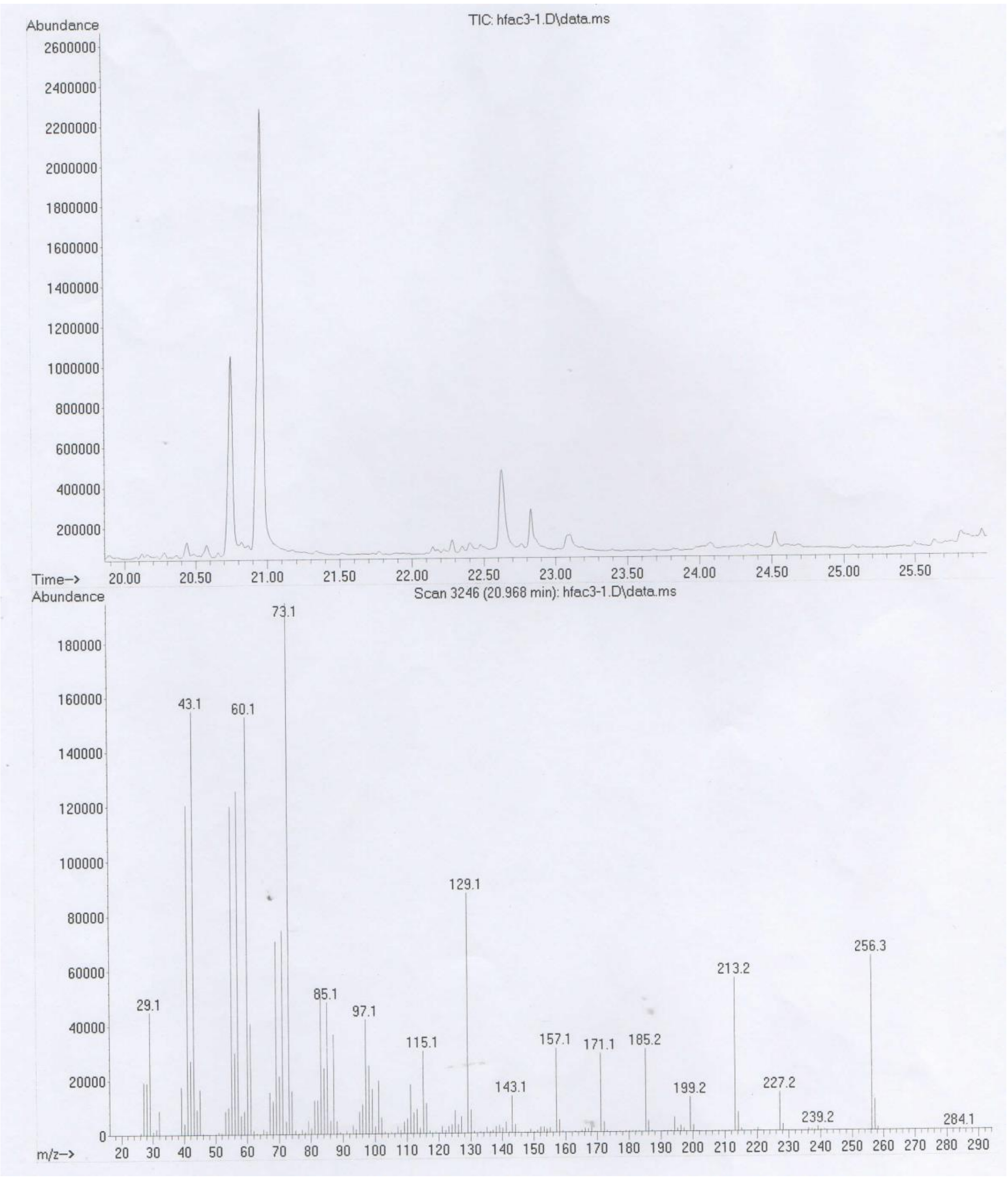

Figure S8. EI-MS spectrum of compound **1**.

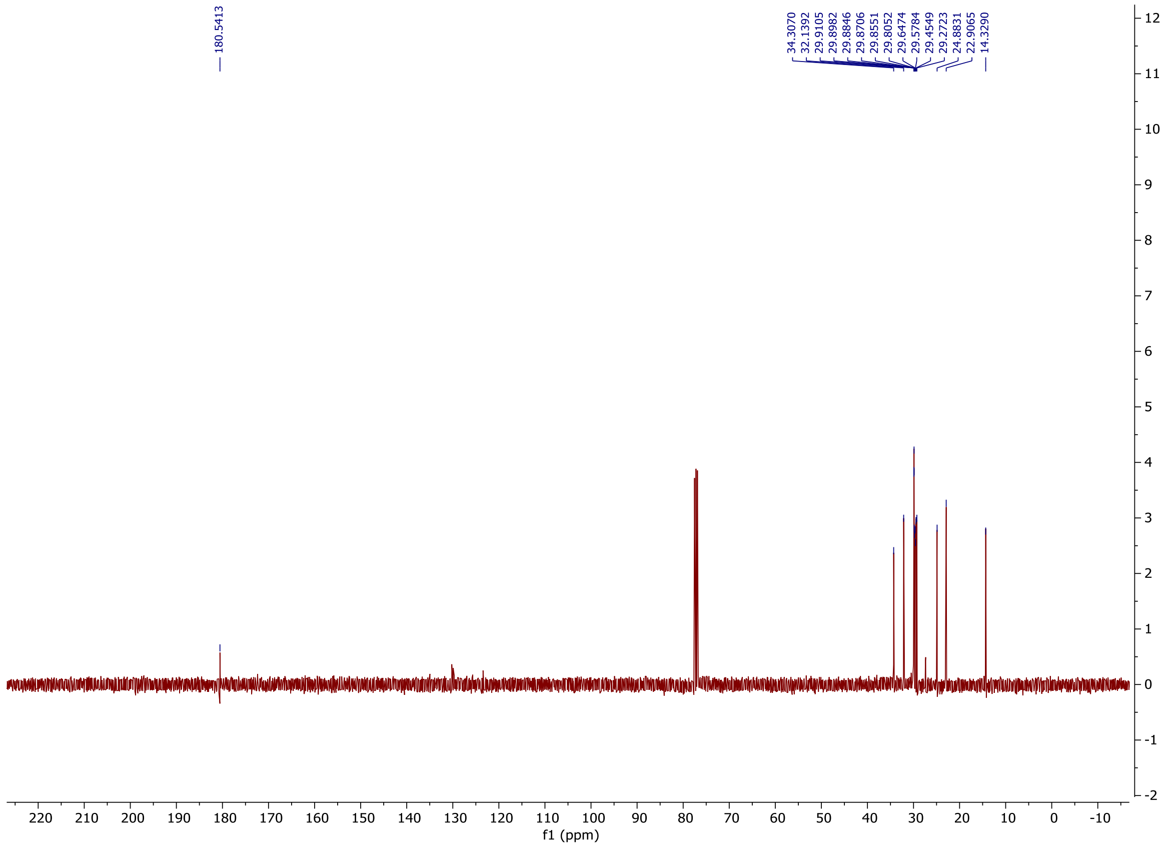

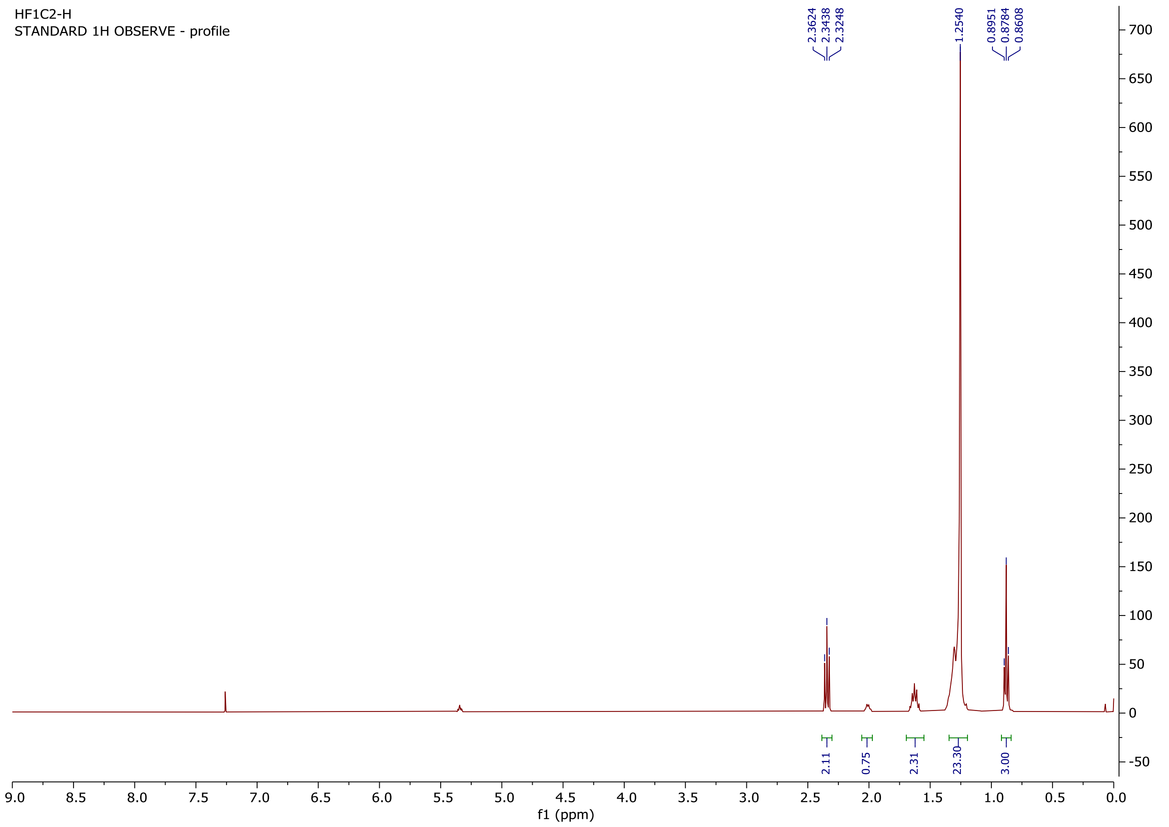

Figure S9. ^{13}C (100MHz in $CDCl_3$)- and ^{1}H (400MHz in $CDCl_3$)-NMR spectrum of compound **2**.

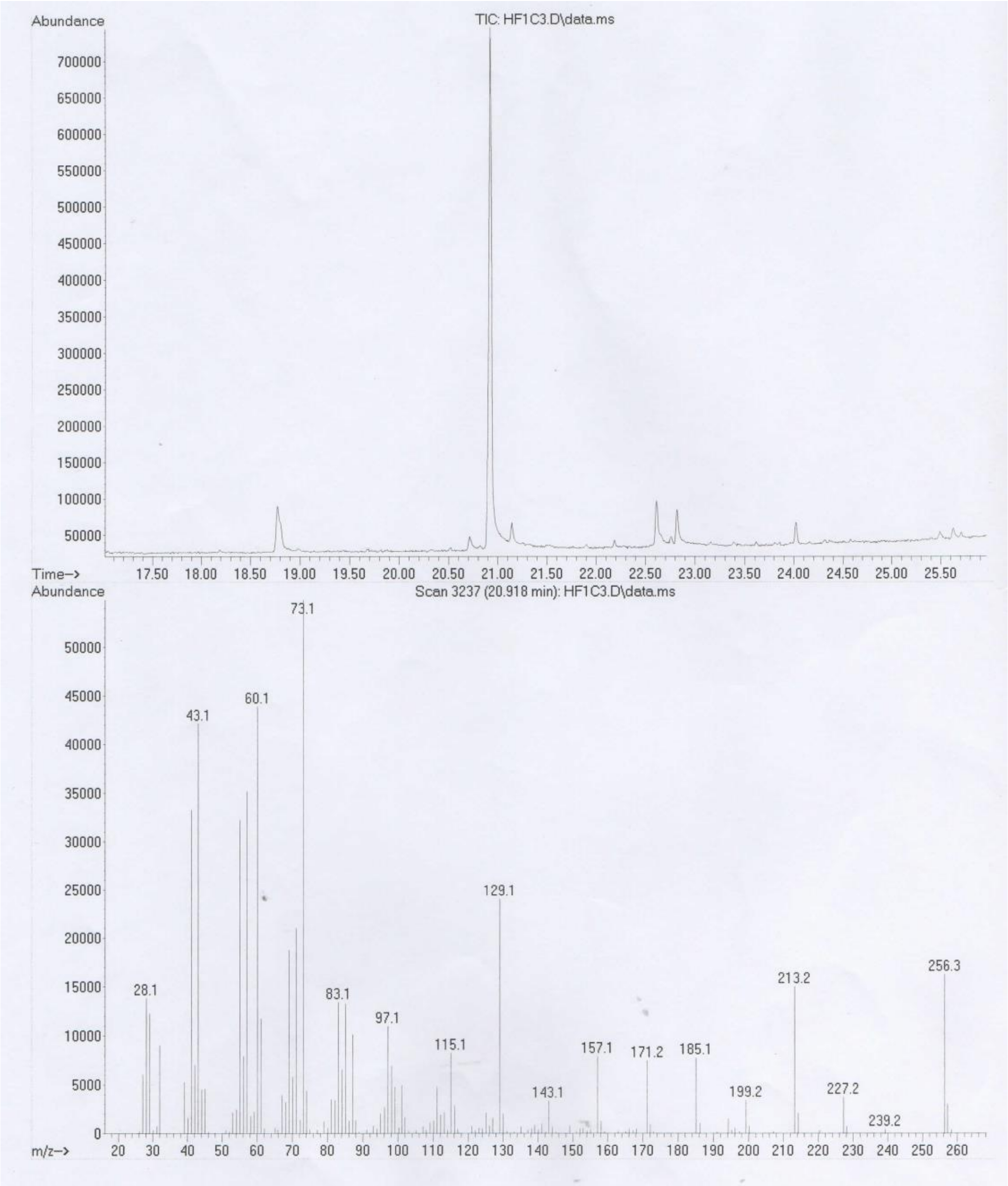

Figure S10. EI-MS spectrum of compound **2**.

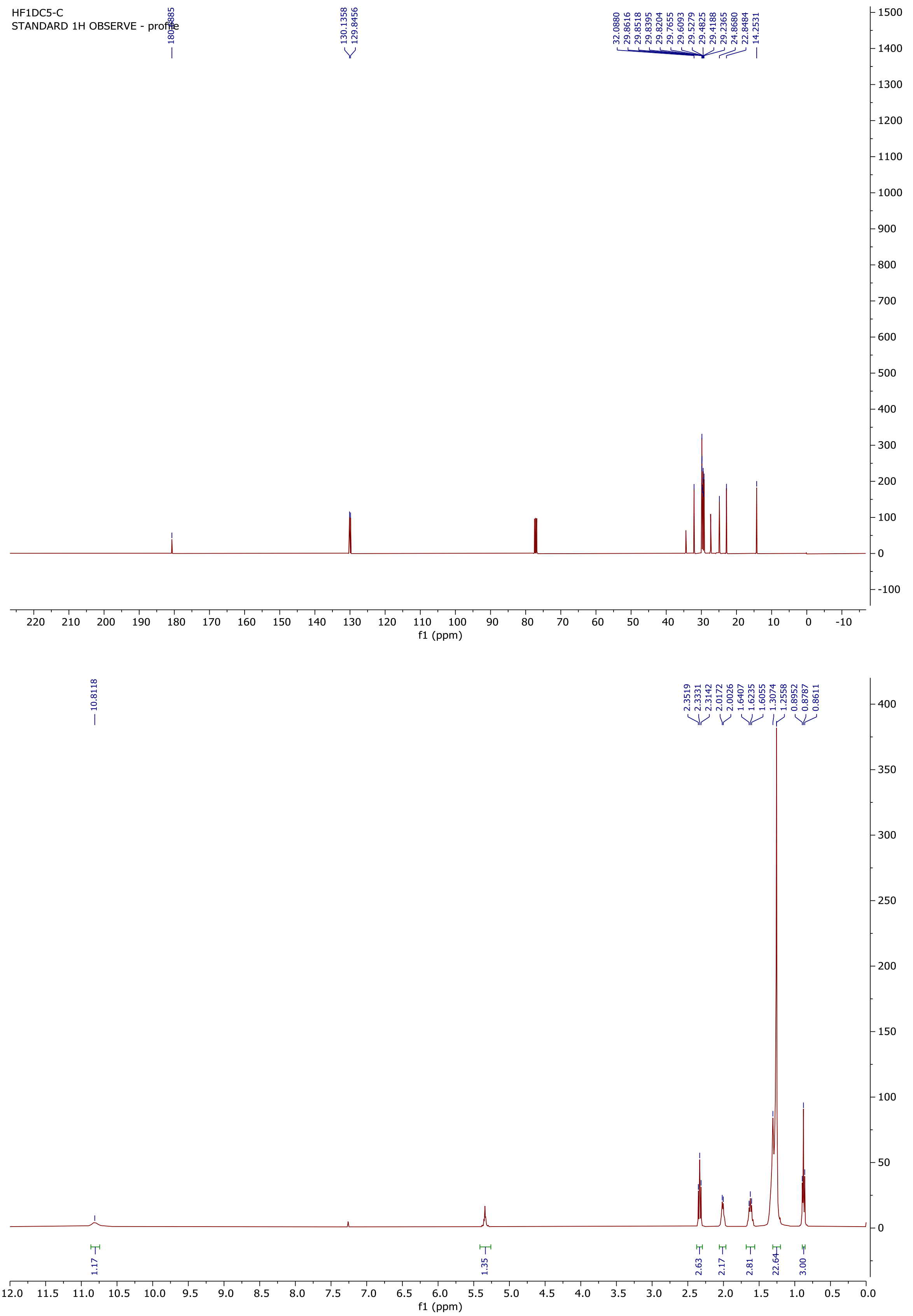

Figure S11. ^{13}C (100MHz in $CDCl_3$)- and ^{1}H (400MHz in $CDCl_3$)-NMR spectrum of compound **3**.

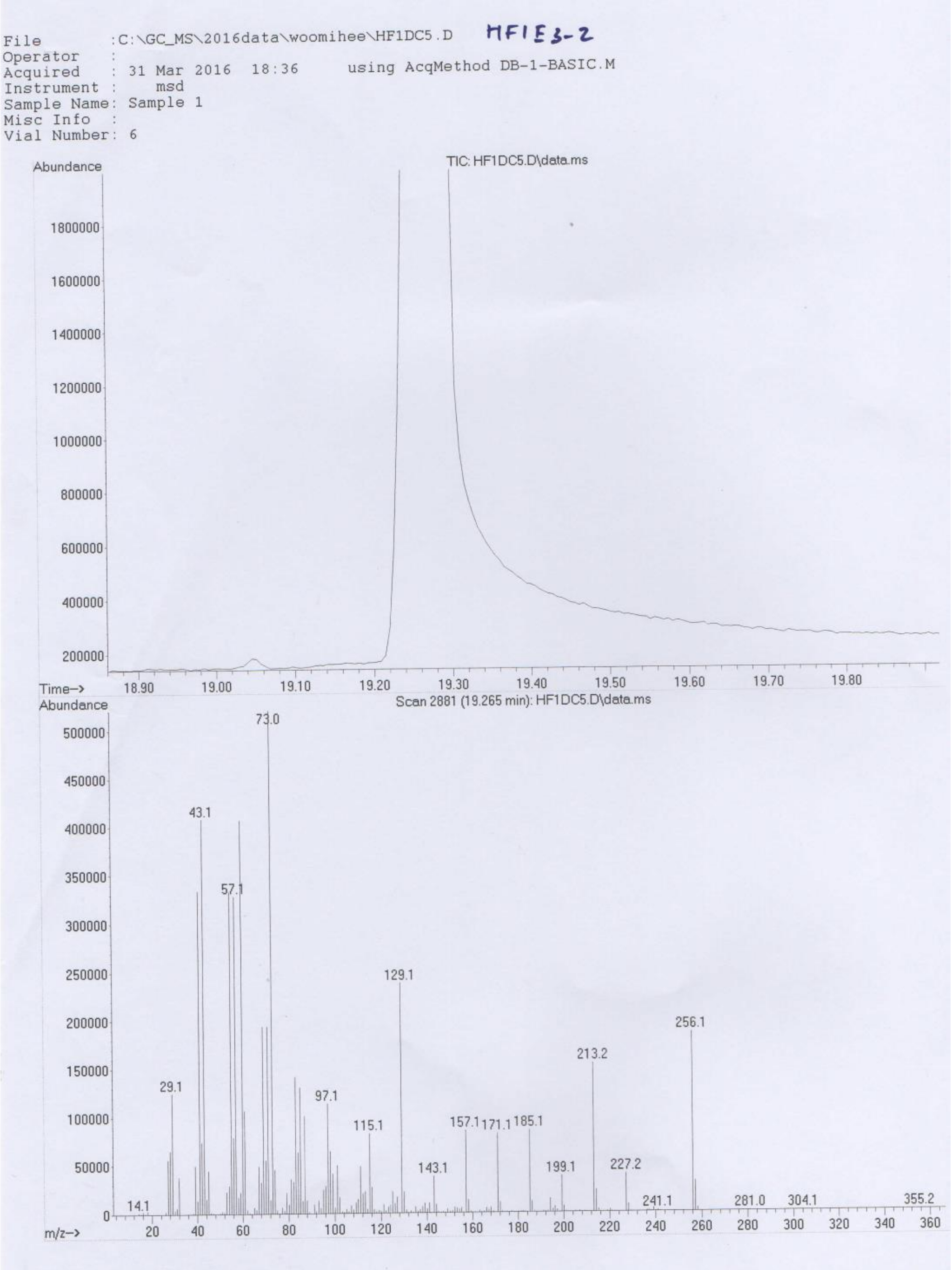

Figure S12. EI-MS spectrum of compound **3**.

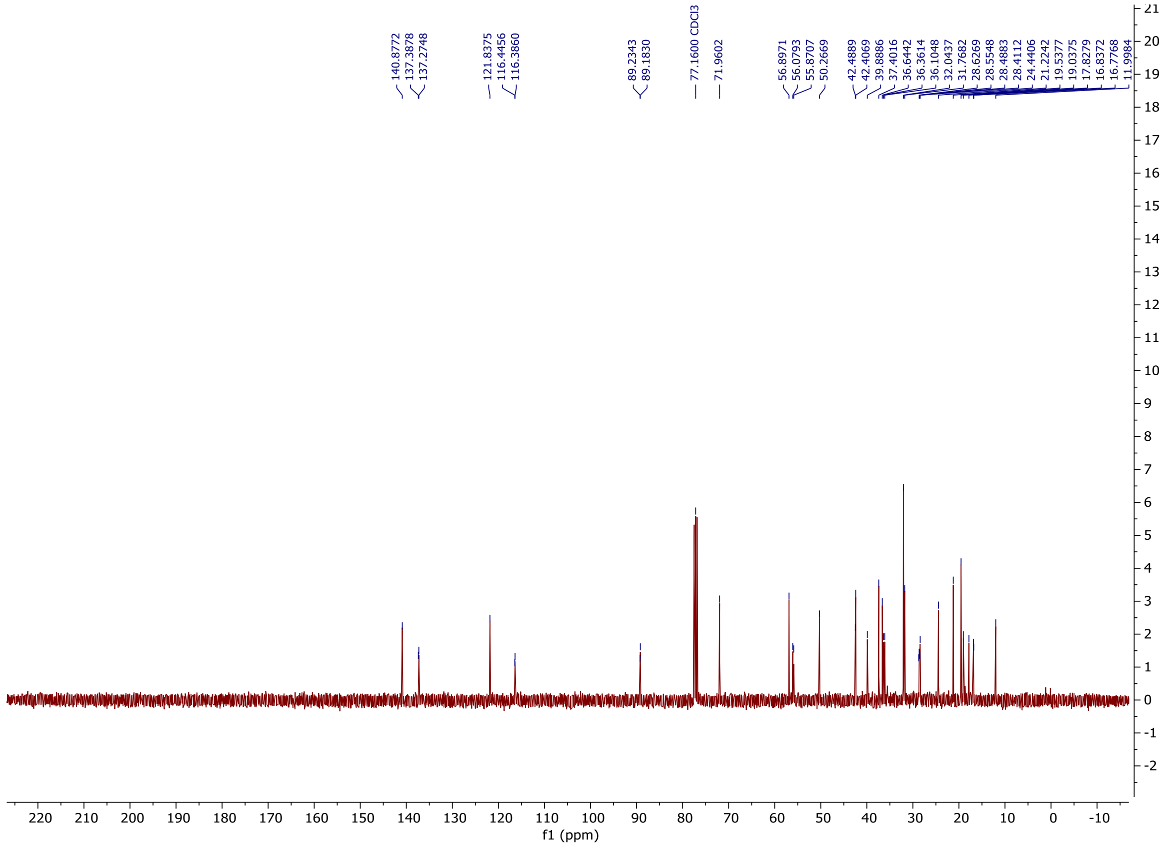

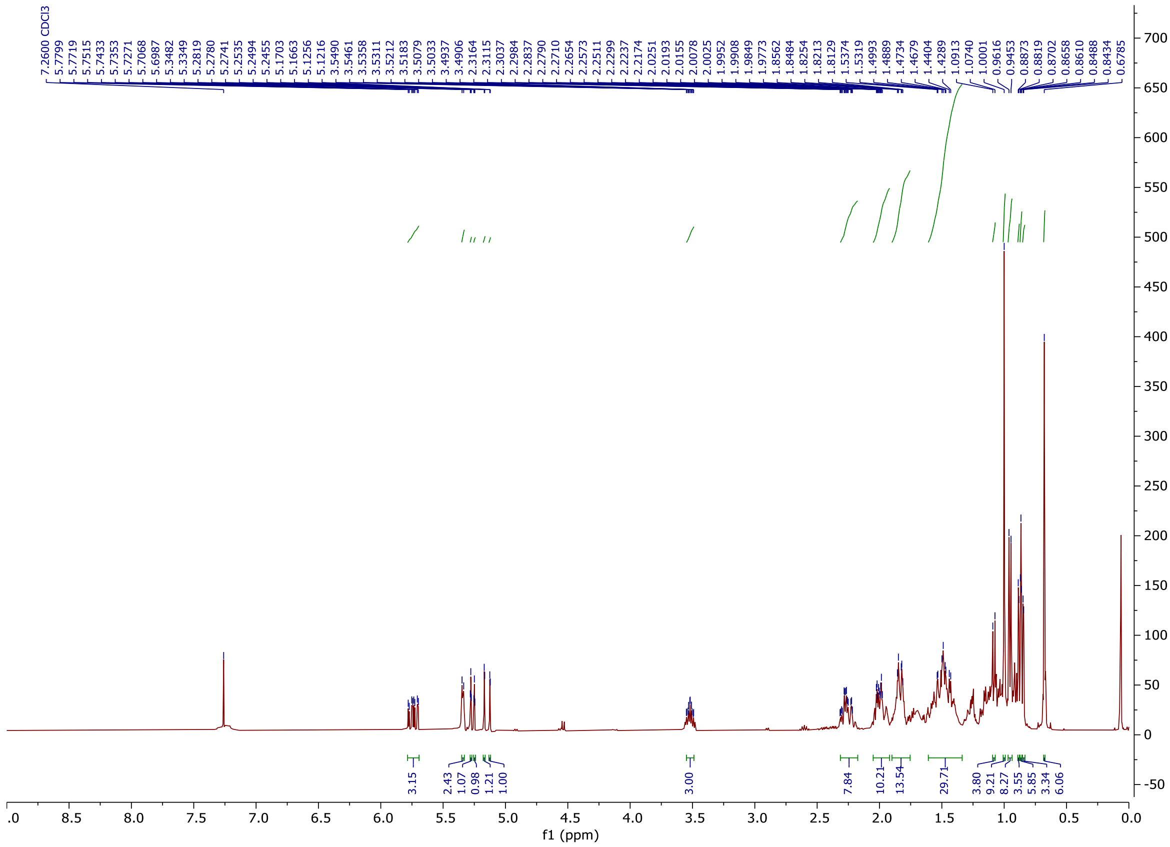

Figure S13. ^{13}C (100MHz in $CDCl_3$)- and ^{1}H (400MHz in $CDCl_3$)-NMR spectrum of compound **4**.

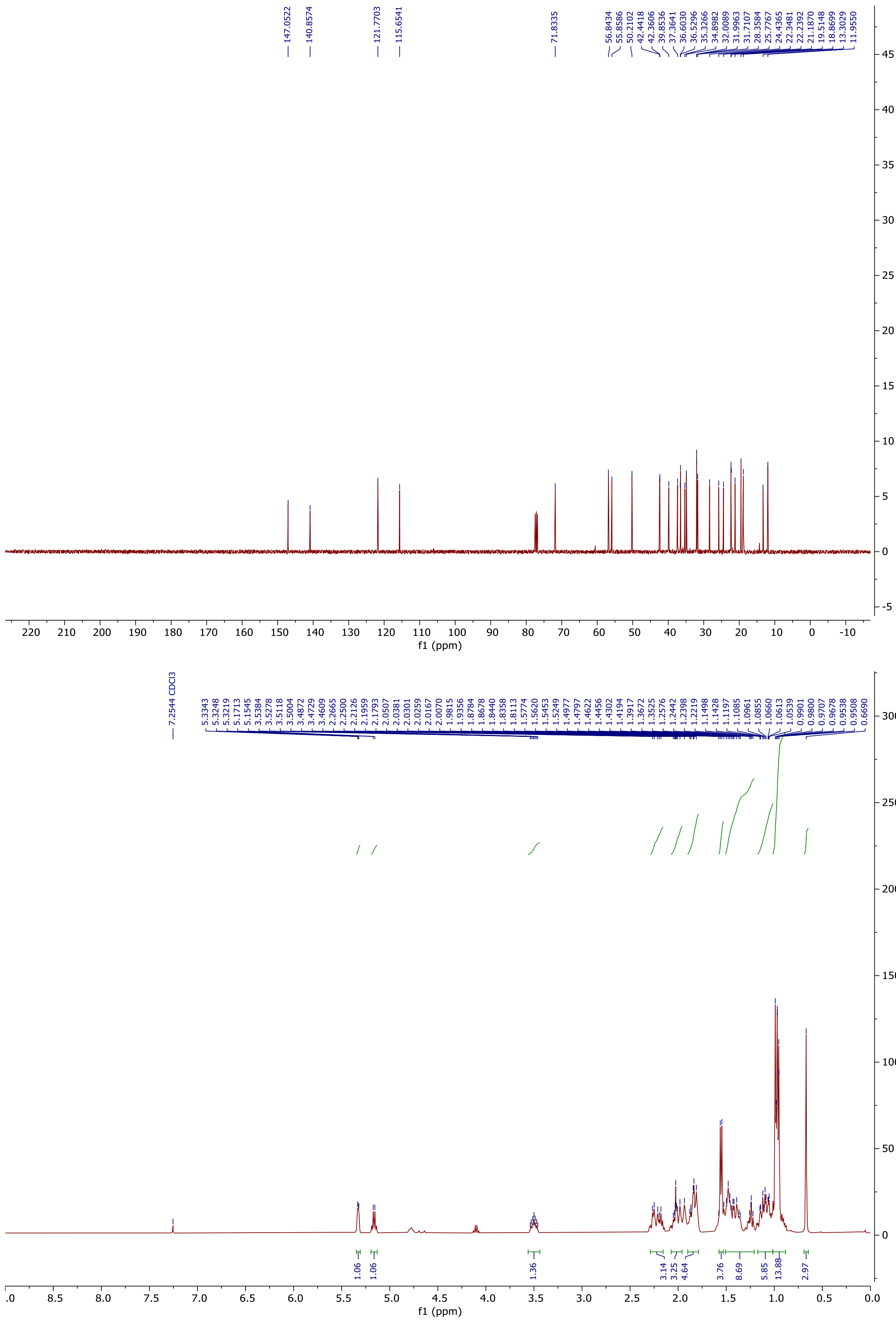

Figure S14. ^{13}C (100MHz in $CDCl_3$)- and ^{1}H (400MHz in $CDCl_3$)-NMR spectrum of compound **5**.

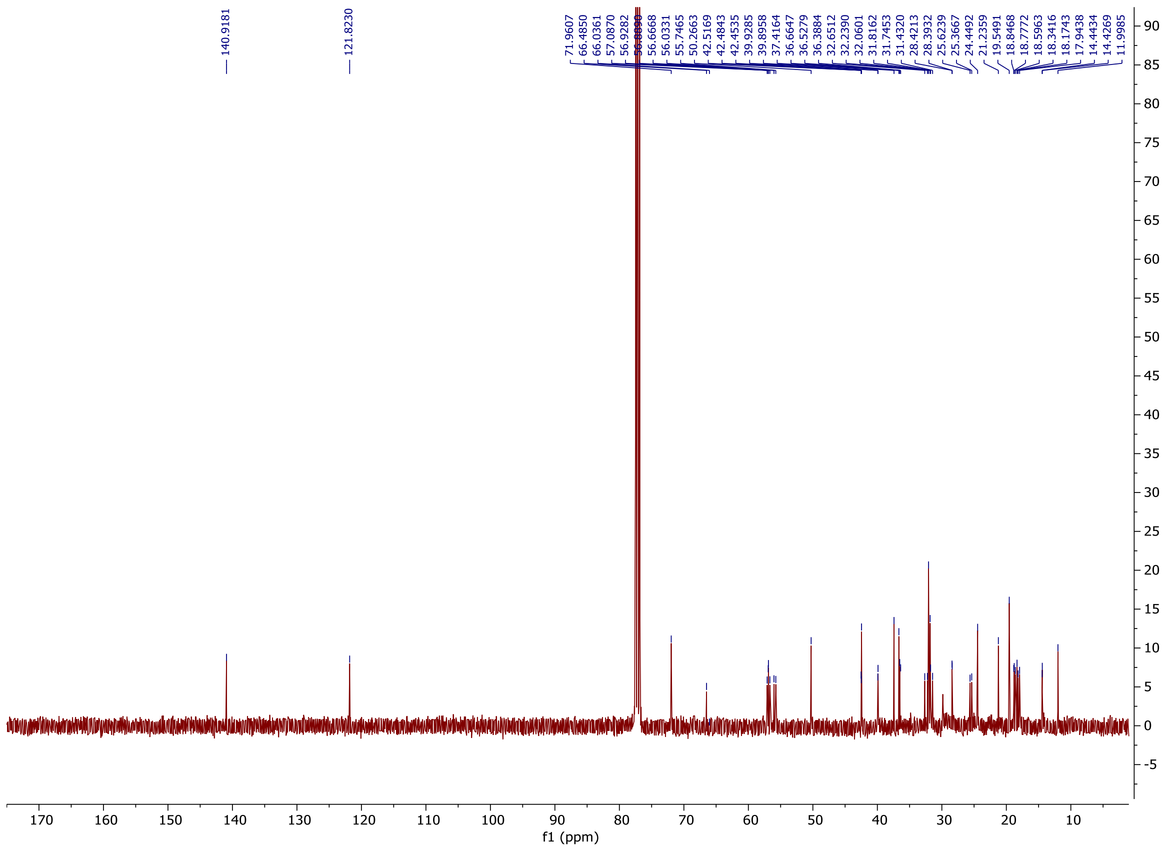

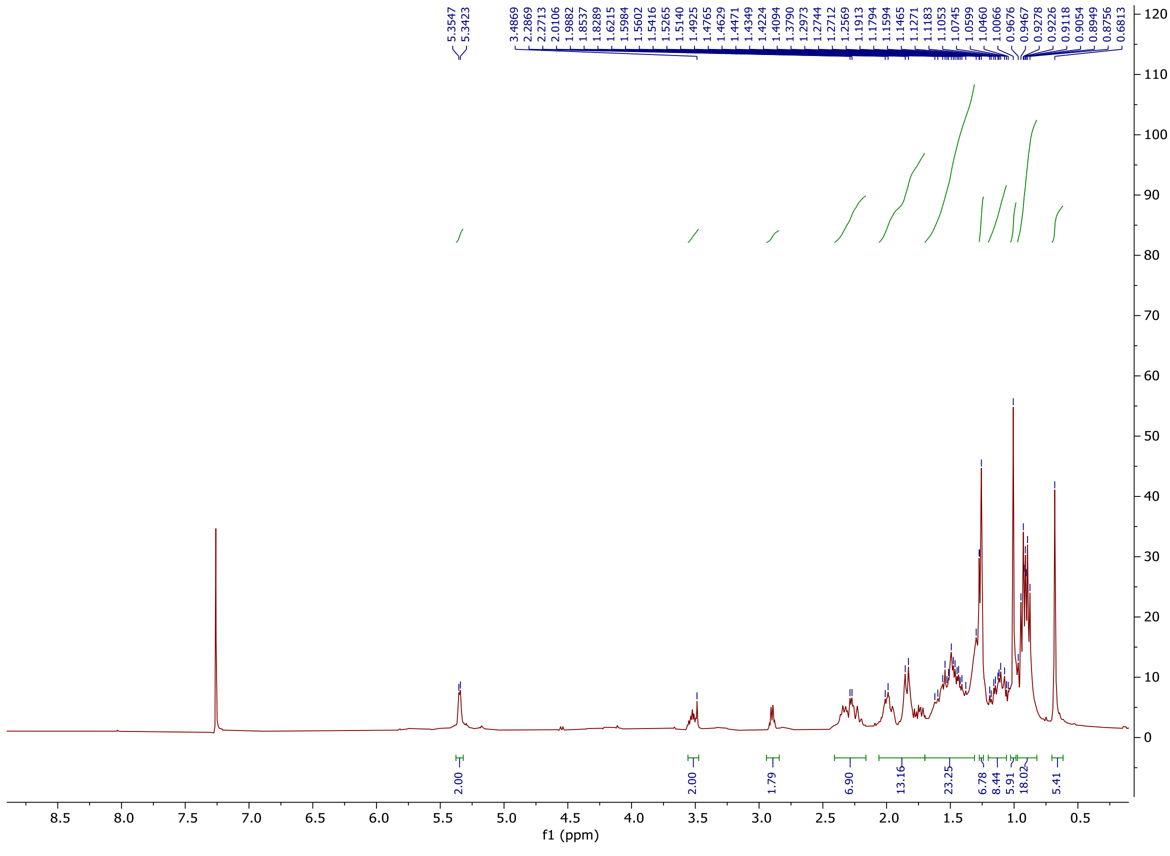

Figure S15. ^{13}C (100MHz in $CDCl_3$)- and ^{1}H (400MHz in $CDCl_3$)-NMR spectrum of compound **6**.

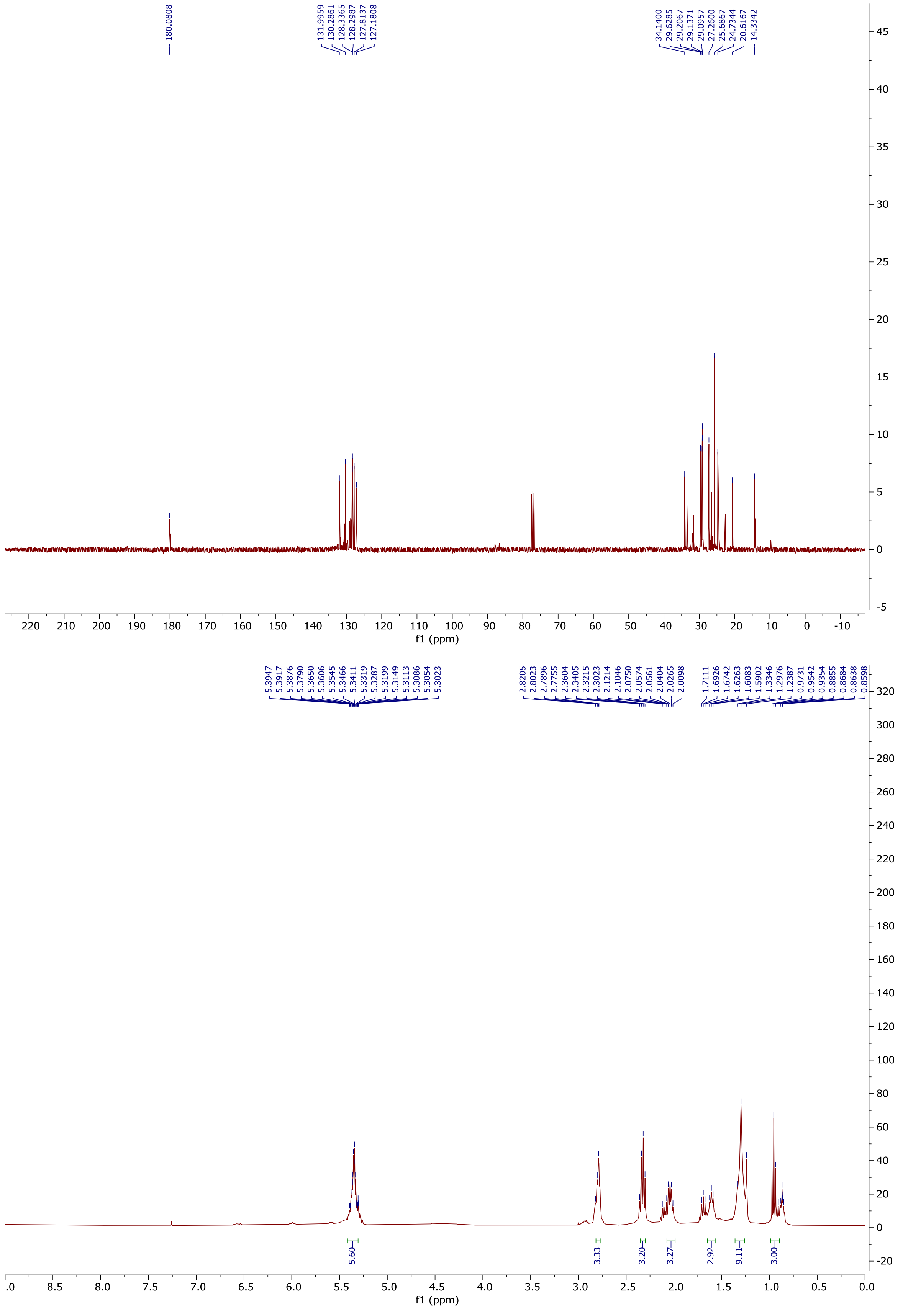

Figure S16. ^{13}C (100MHz in $CDCl_3$)- and ^{1}H (400MHz in $CDCl_3$)-NMR spectrum of compound **7.**

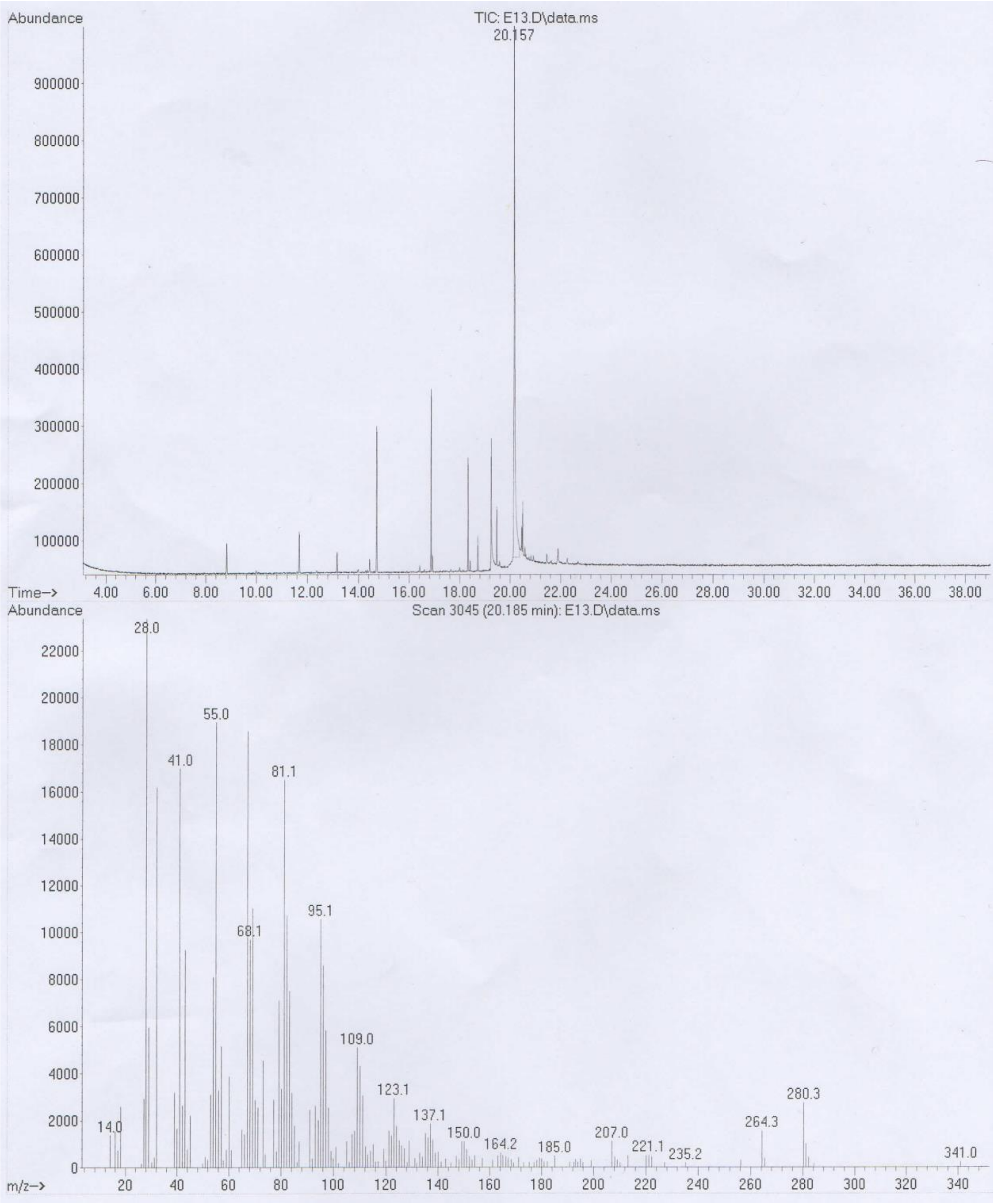

Figure S17. EI-MS spectrum of compound **7**.

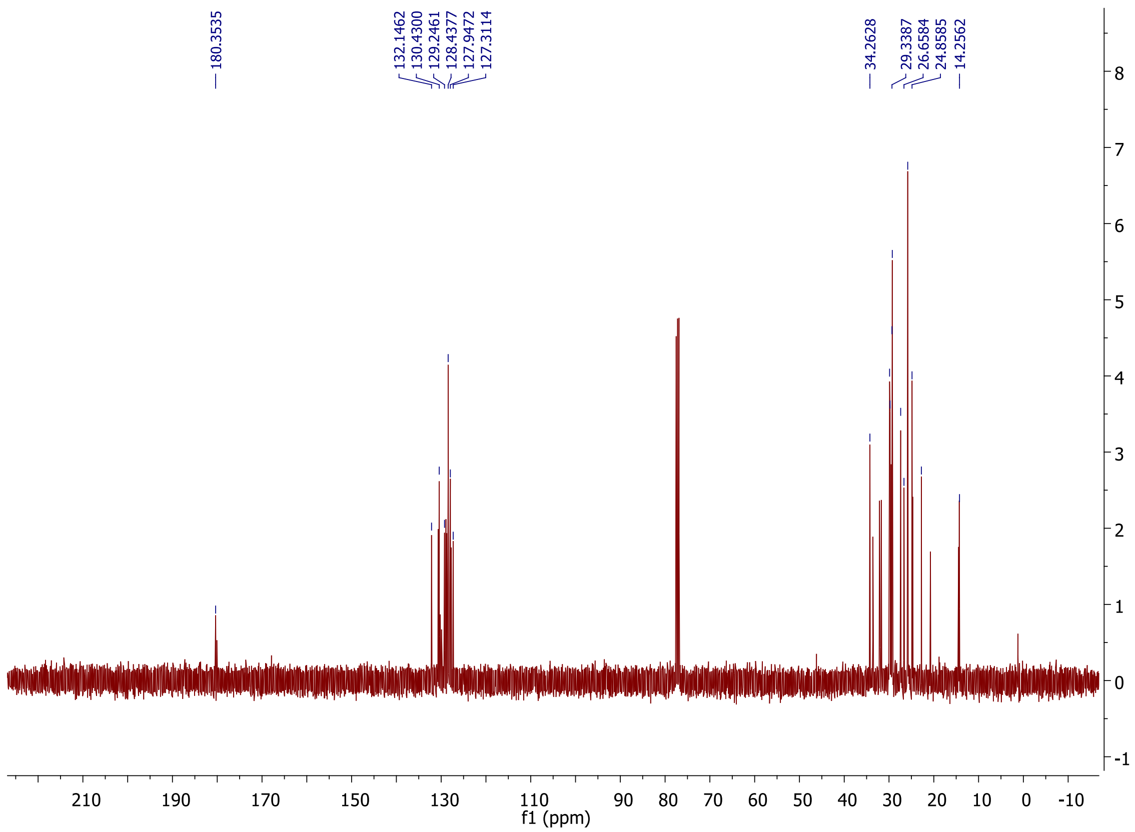

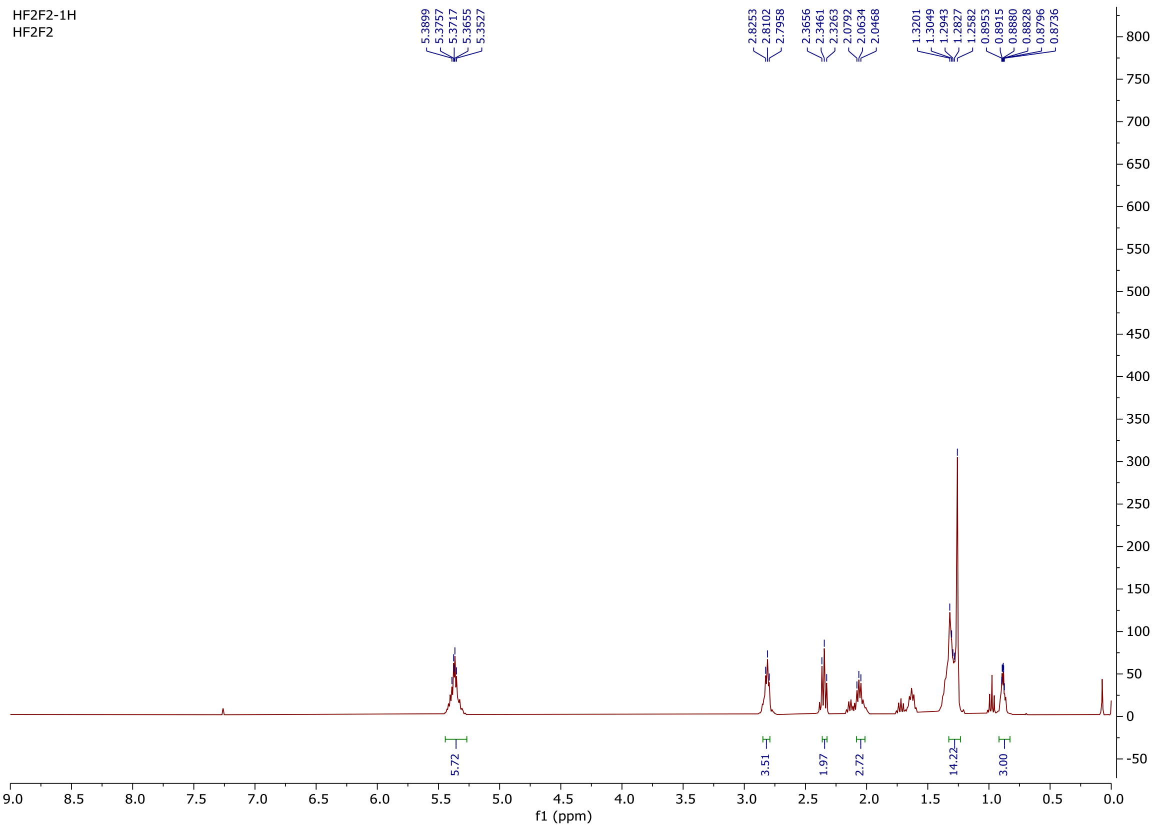

Figure S18. ^{13}C (100MHz in $CDCl_3$)- and ^{1}H (400MHz in $CDCl_3$)-NMR spectrum of compound **8**.

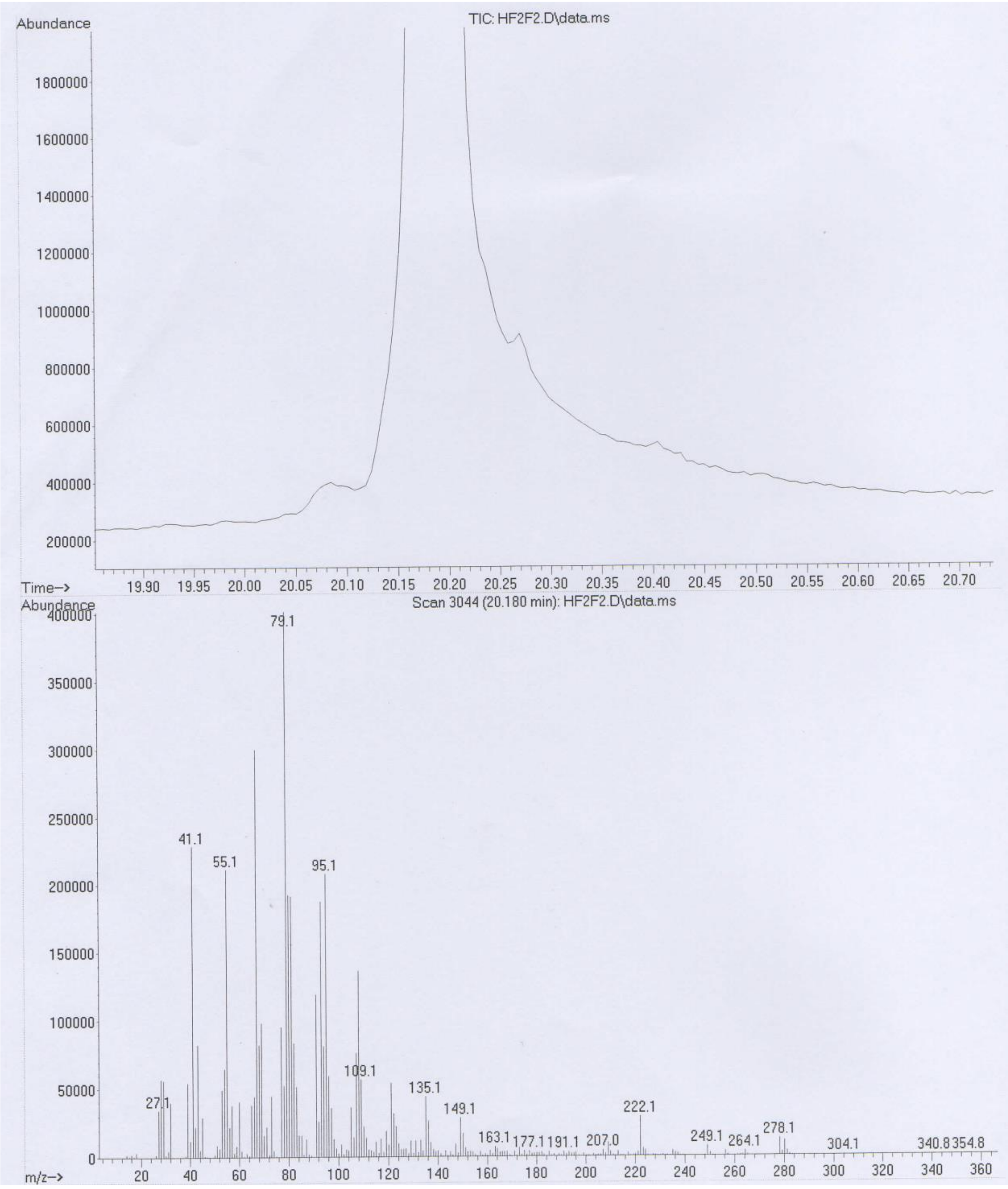

Figure S19. EI-MS spectrum of compound **8**.

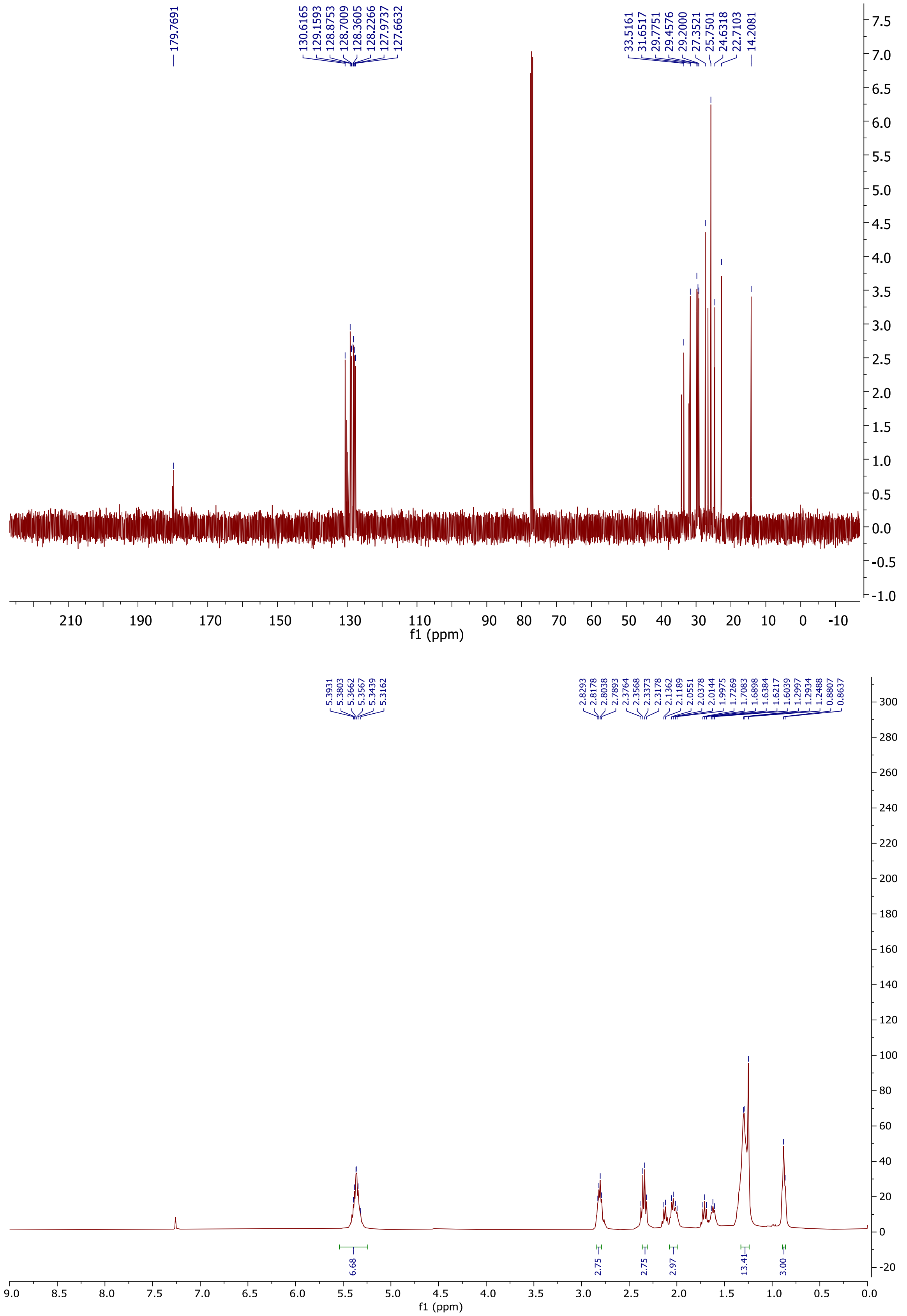

Figure S20. ^{13}C (100MHz in $CDCl_3$)- and ^{1}H (400MHz in $CDCl_3$)-NMR spectrum of compound **9**.

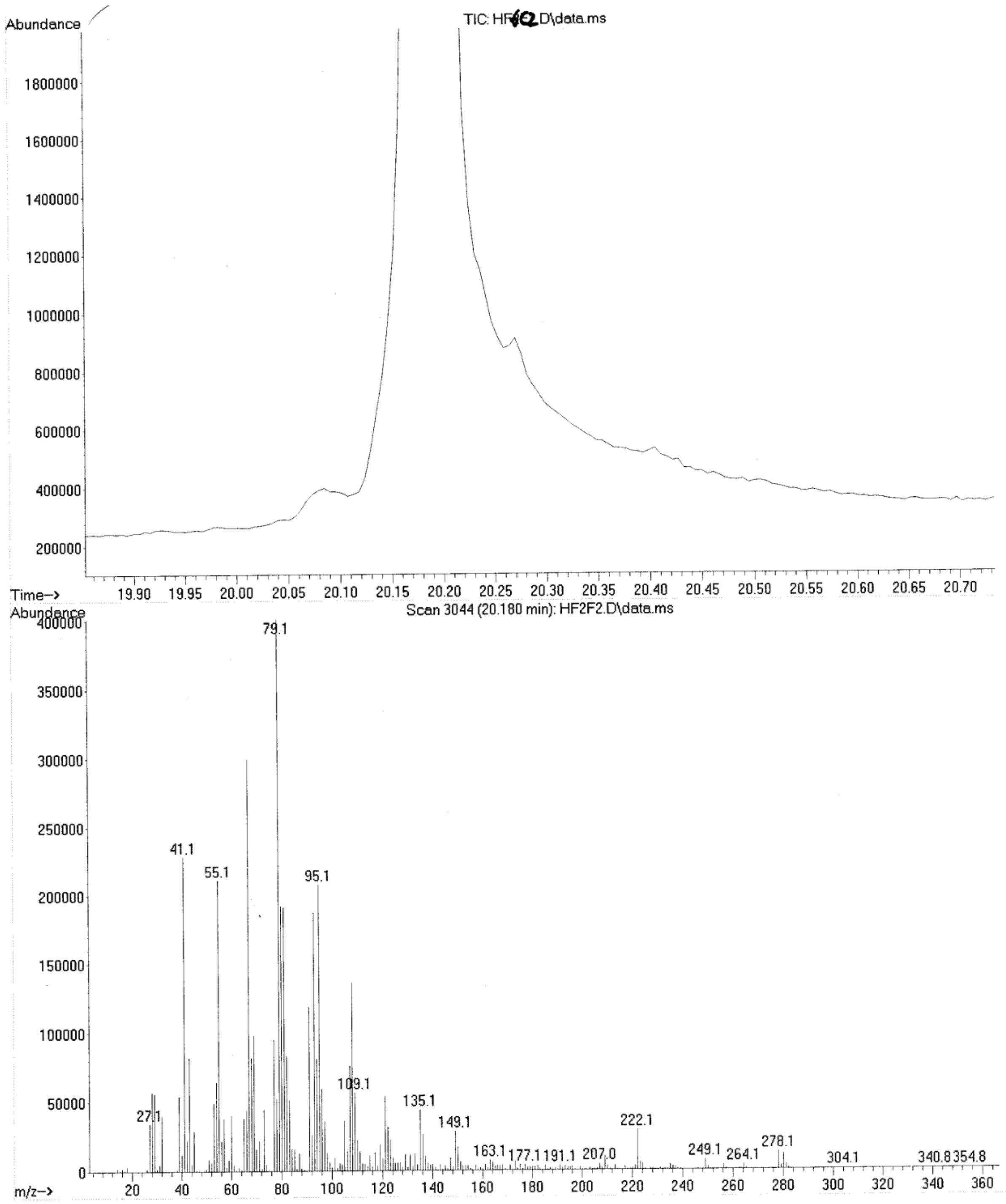

Figure S21. EI-MS spectrum of compound **9.**

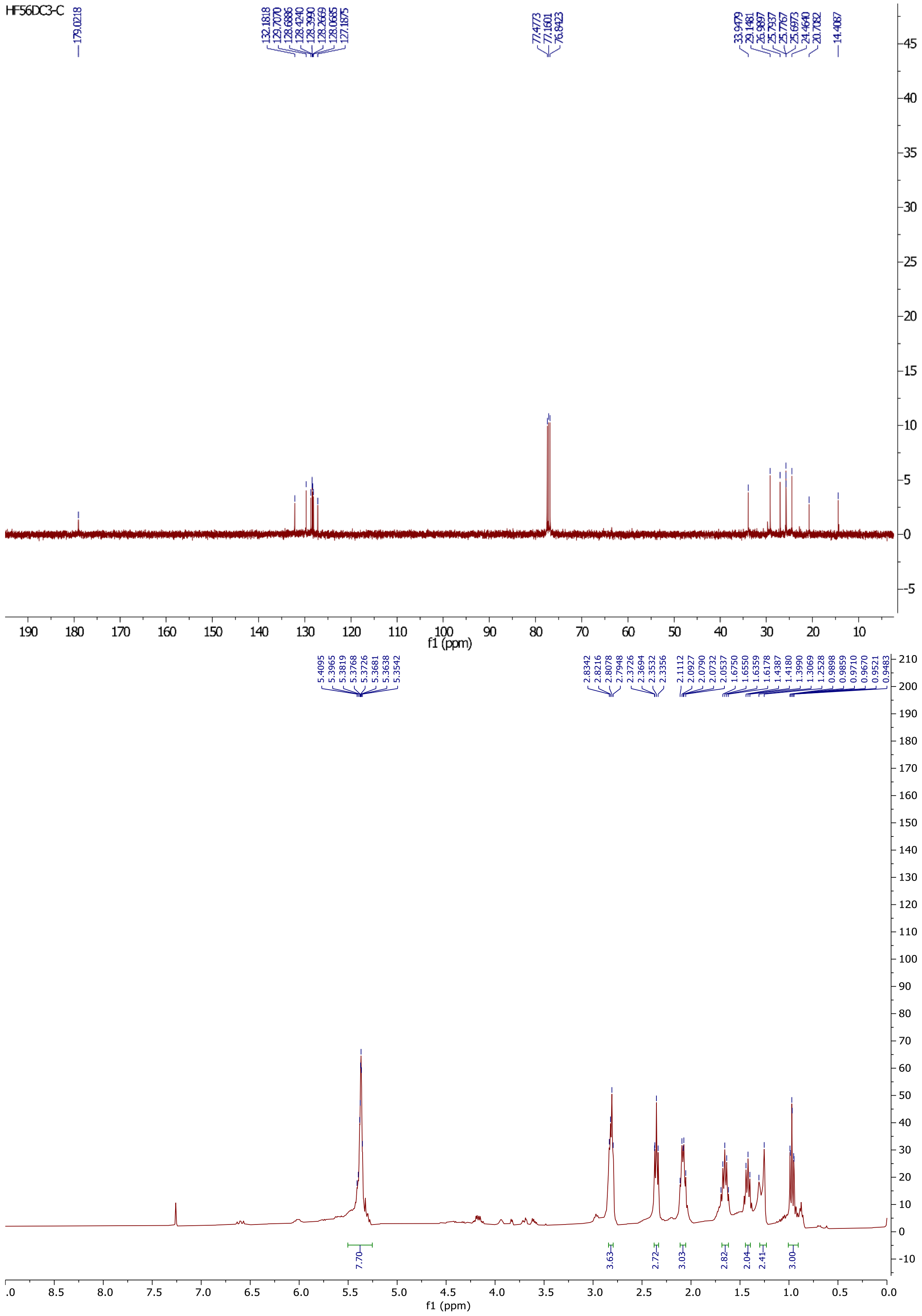

Figure S22. ^{13}C (100MHz in $CDCl_3$)- and ^{1}H (400MHz in $CDCl_3$)-NMR spectrum of compound **10**.

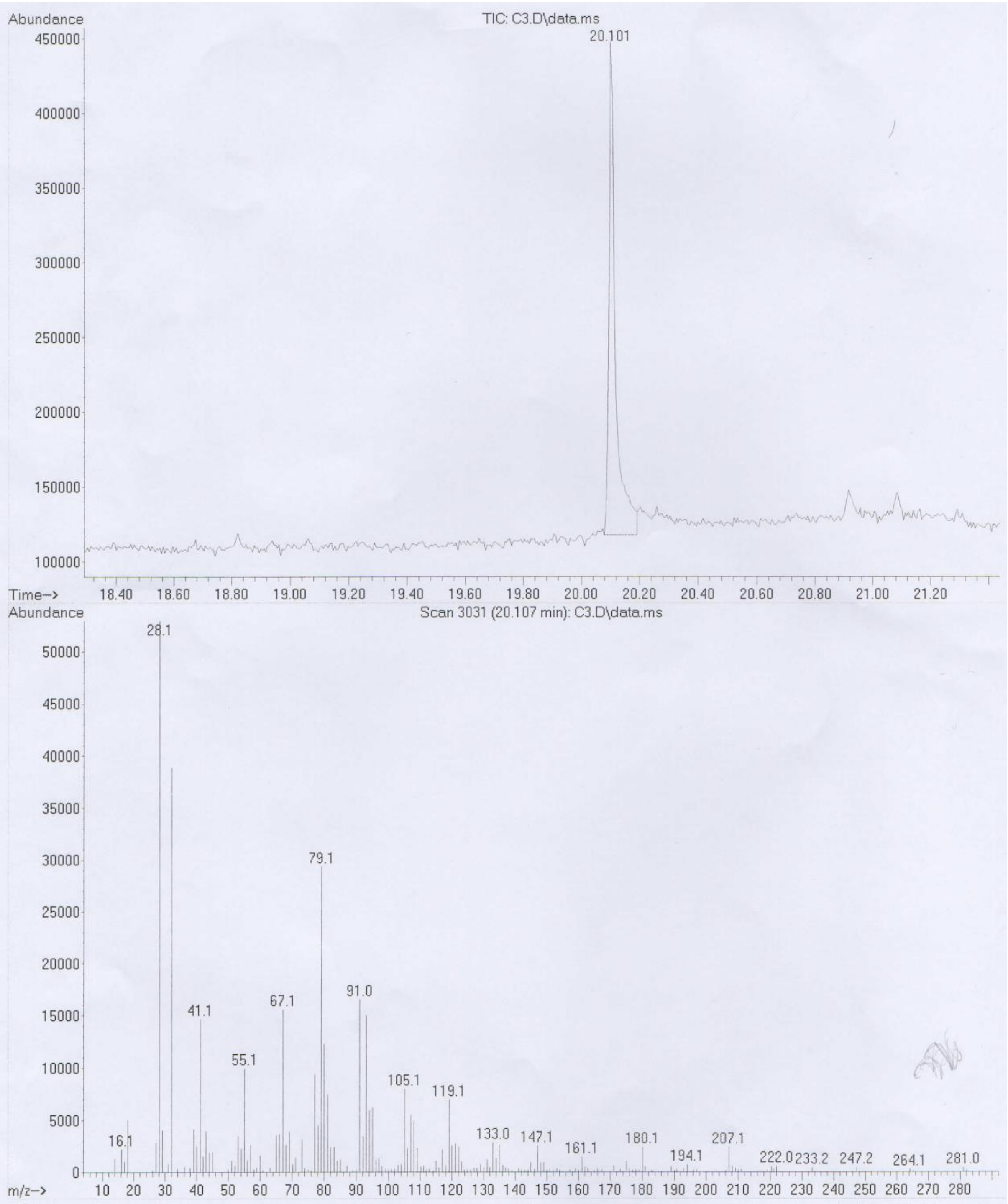

Figure S23. EI-MS spectrum of compound **10**.

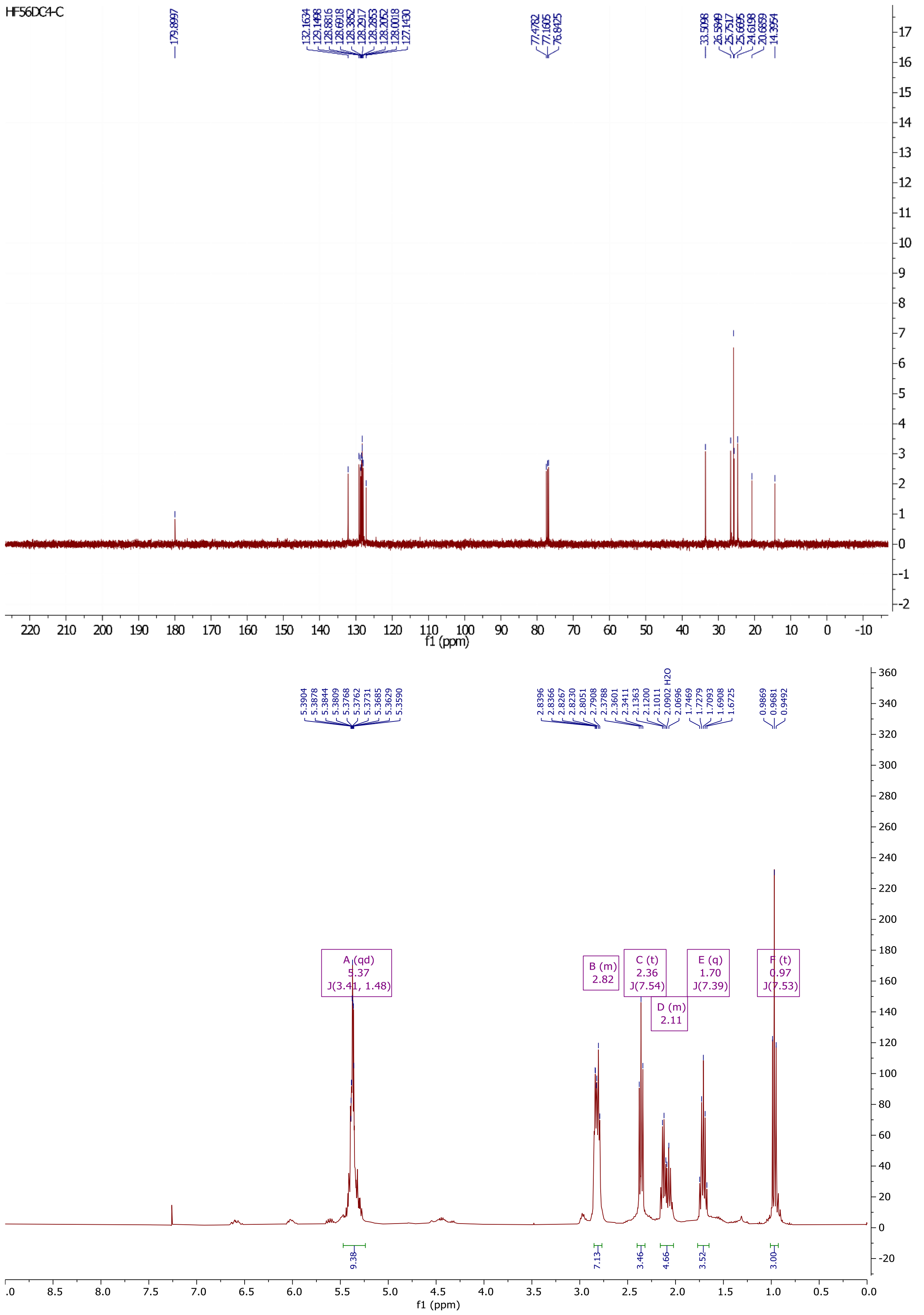

Figure S24. ^{13}C (100MHz in $CDCl_3$)- and ^{1}H (400MHz in $CDCl_3$)-NMR spectrum of compound **11**.

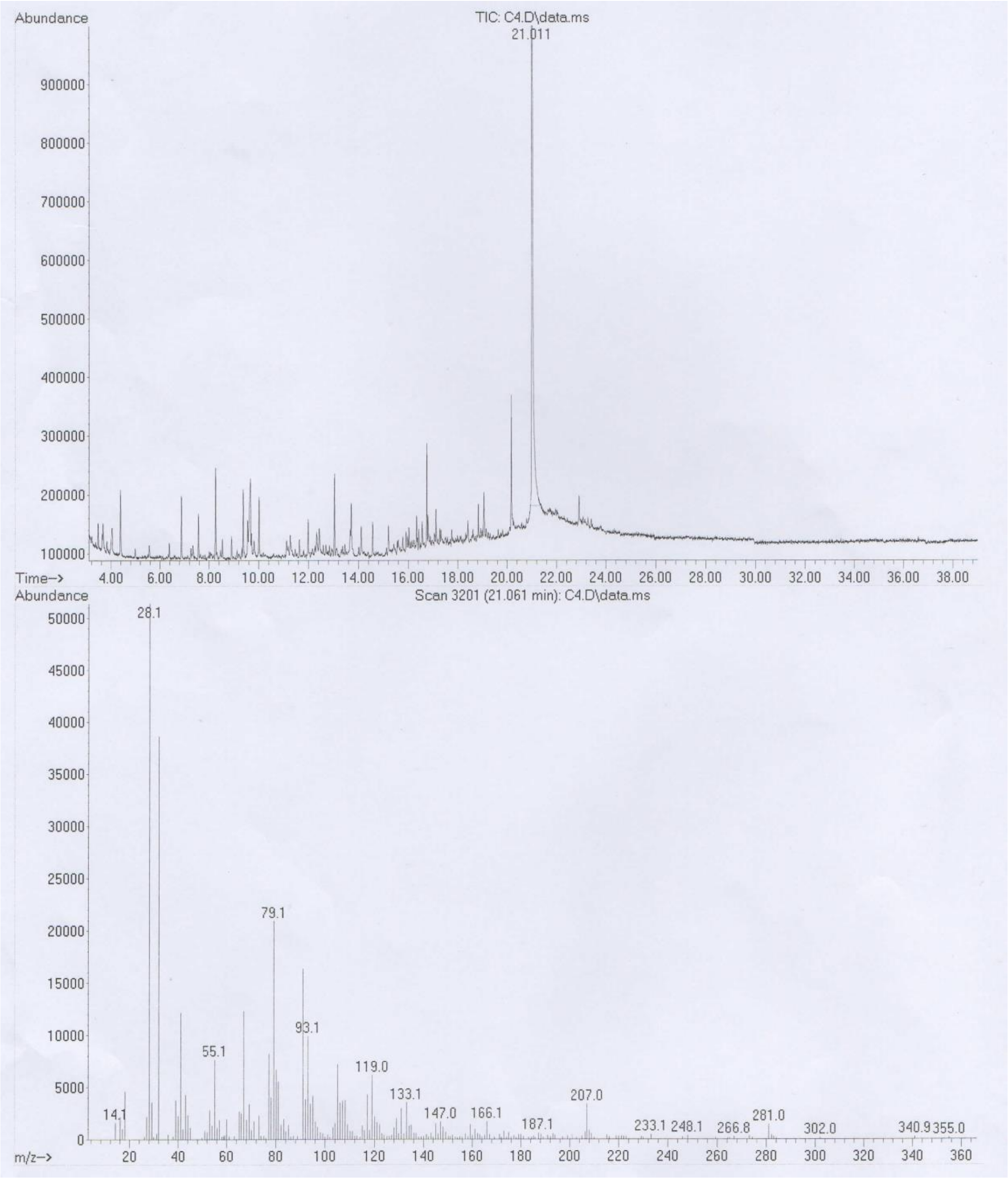

Figure S25. EI-MS spectrum of compound **11**.

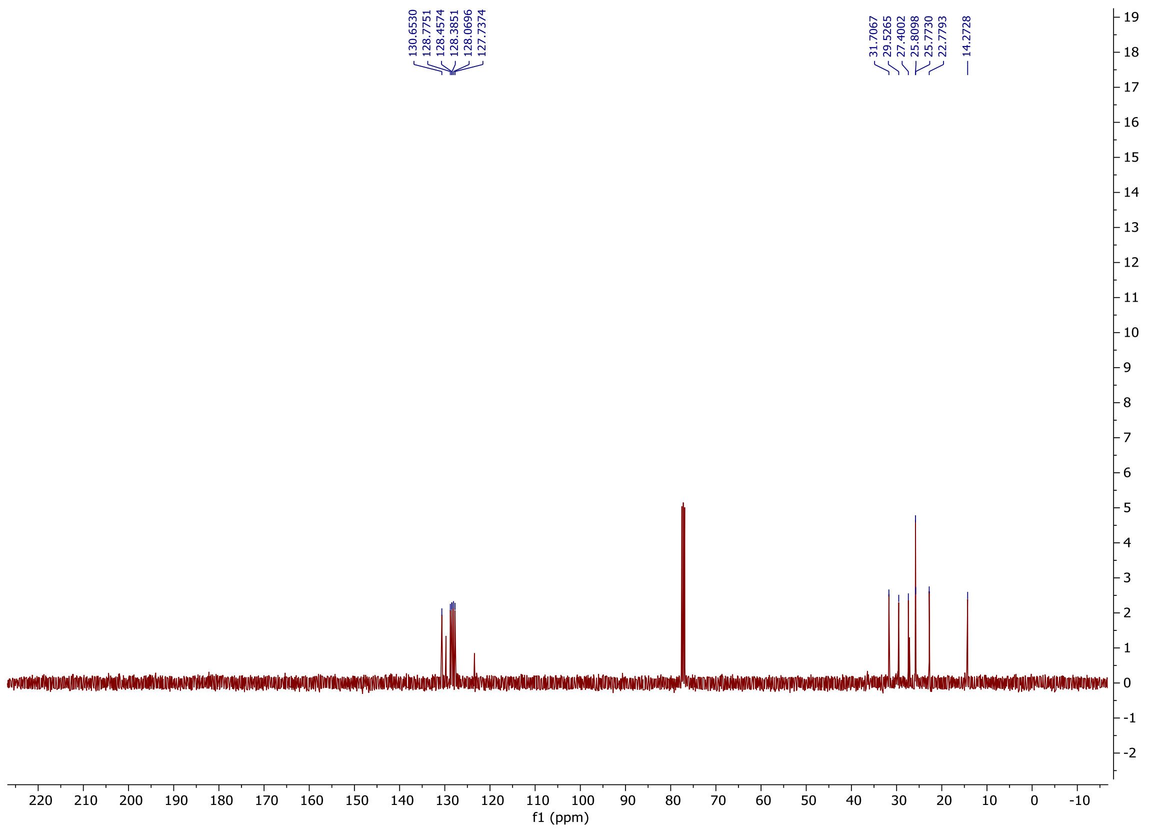

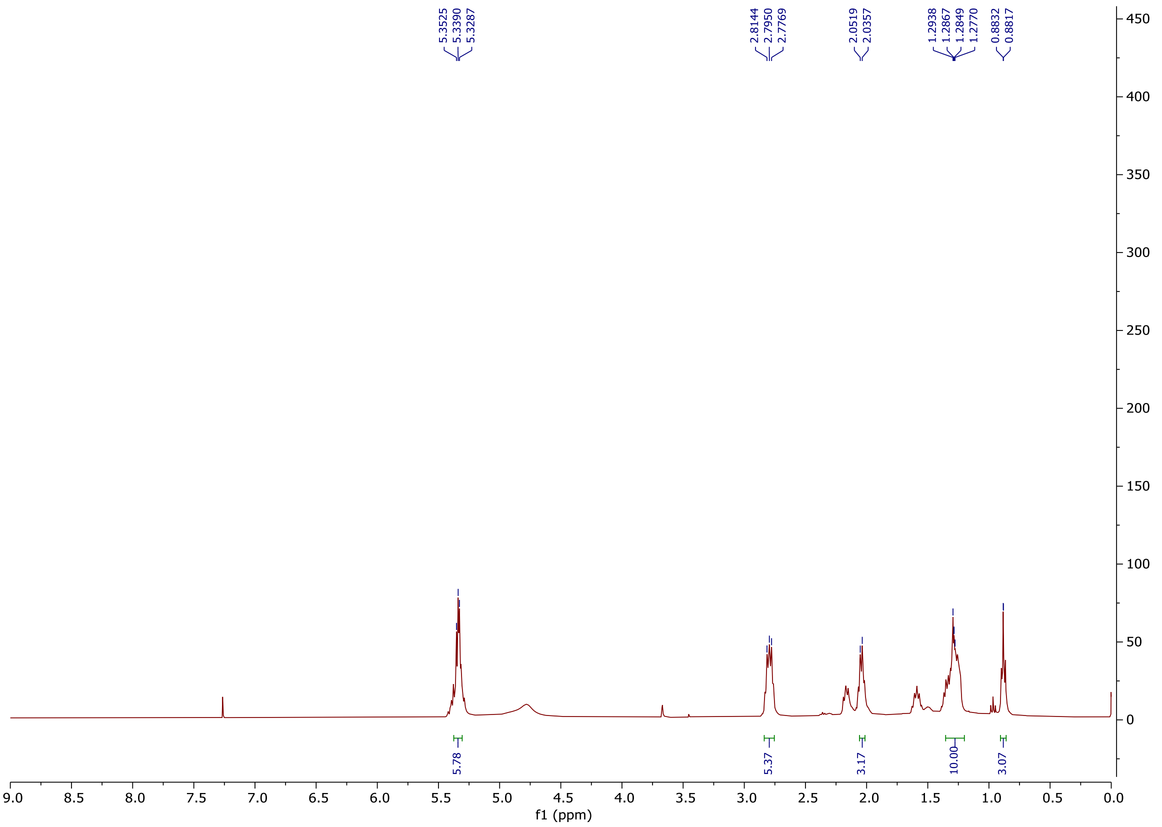

Figure S26. ^{13}C (100MHz in $CDCl_3$)- and ^{1}H (400MHz in $CDCl_3$)-NMR spectrum of compound **12**.

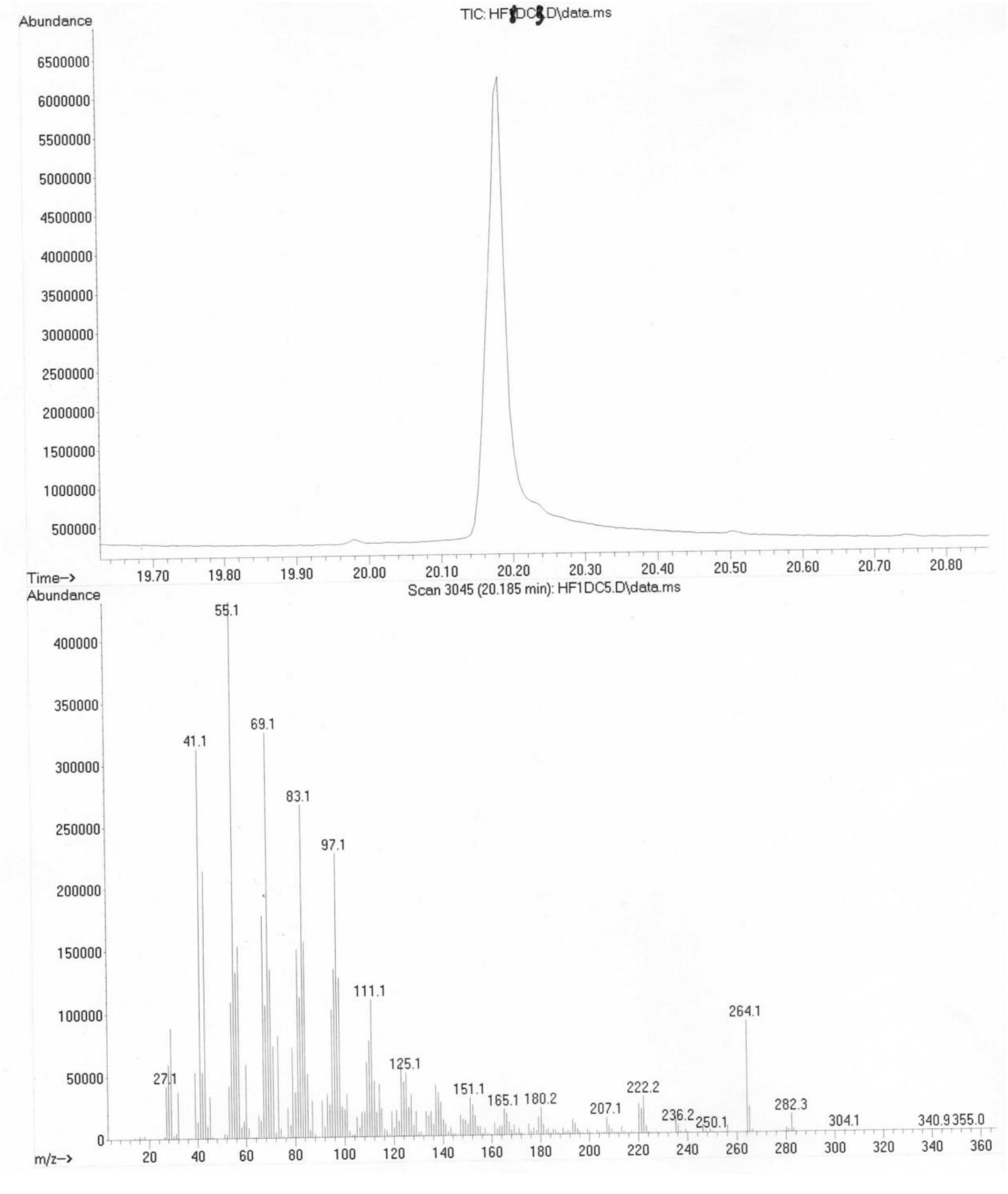

Figure S27. EI-MS spectrum of compound **12**.

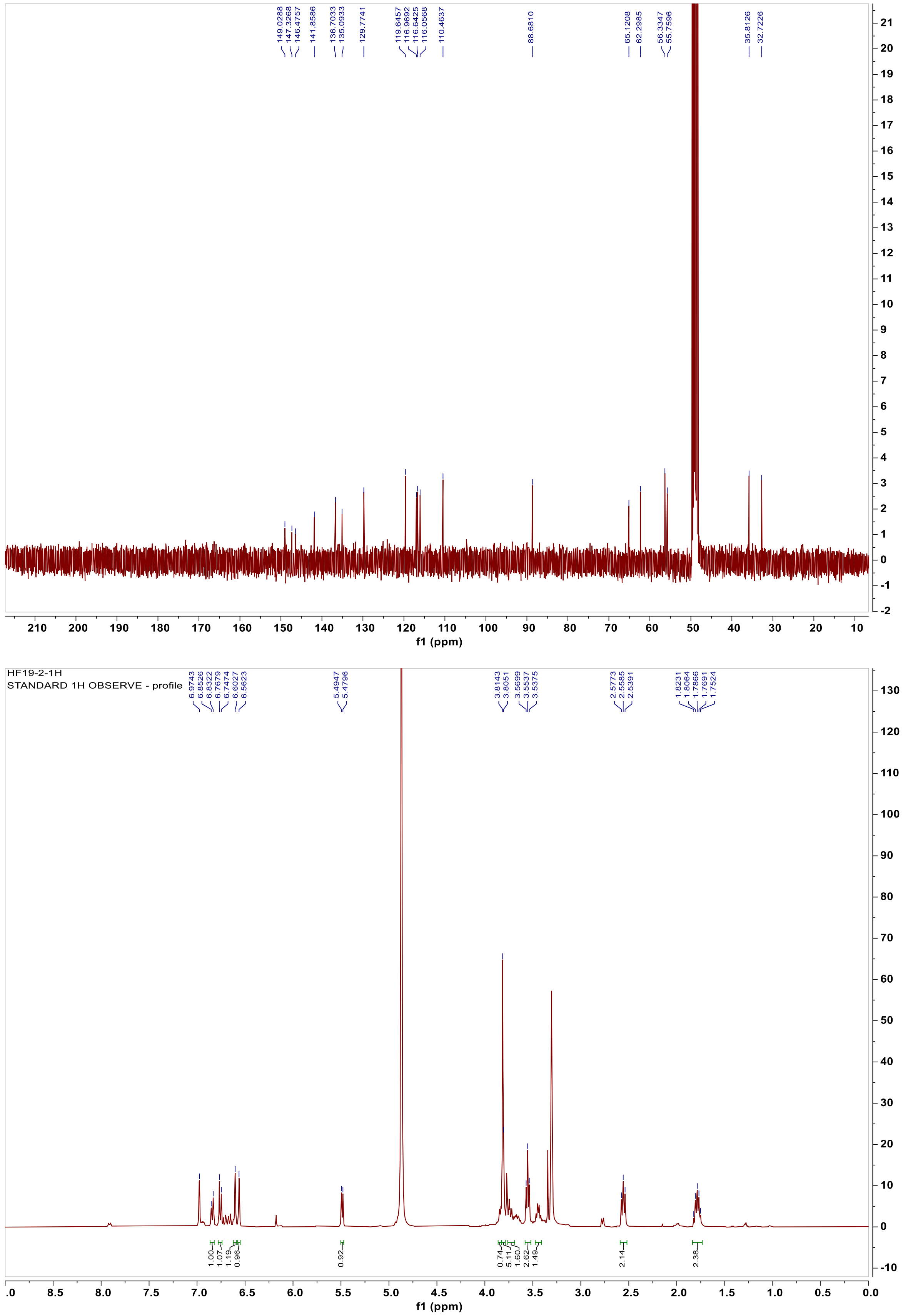

Figure S28. ^{13}C (100MHz in CD_3OD)- and ^{1}H (400MHz in CD_3OD)-NMR spectrum of compound **14**.

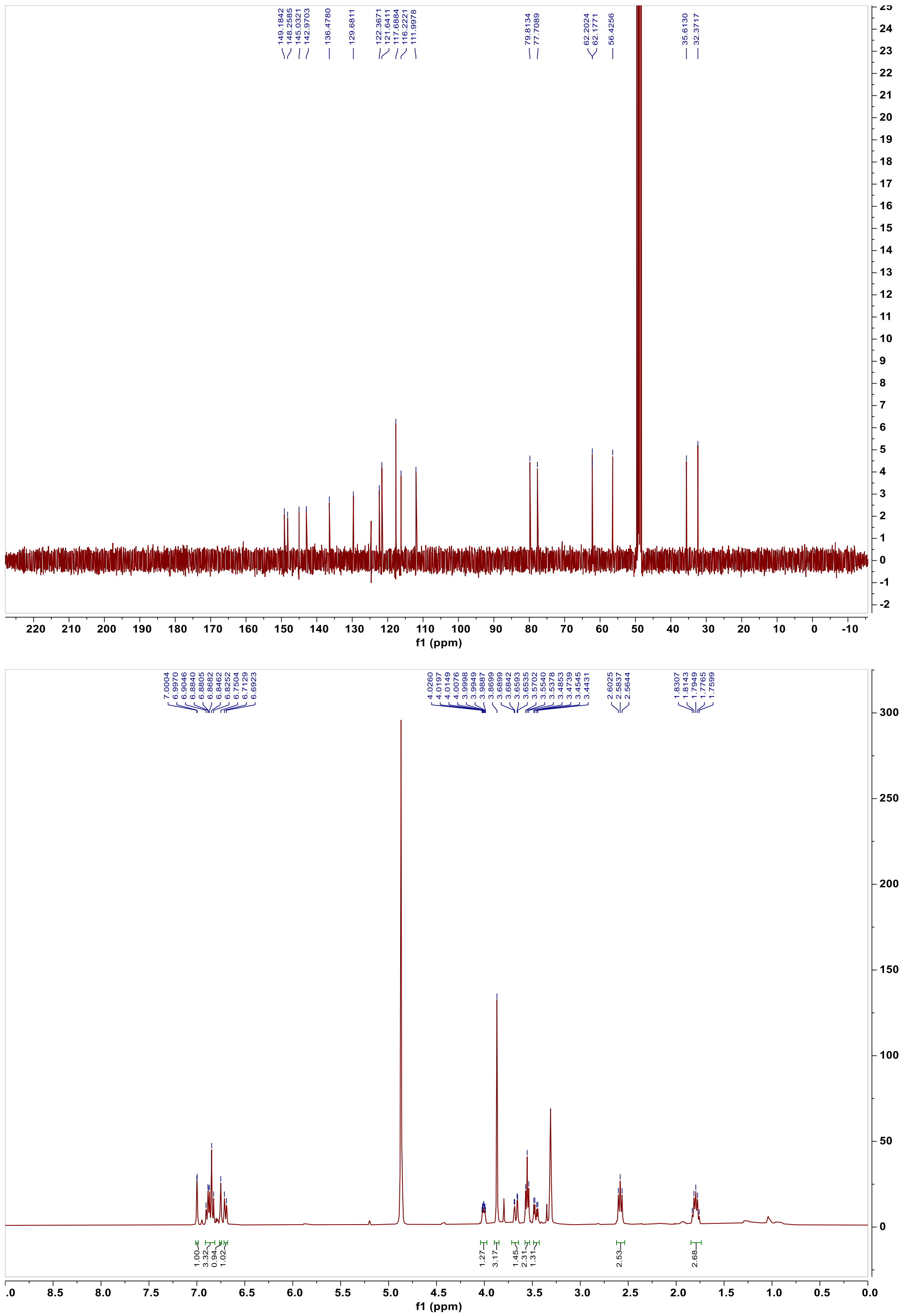

Figure S29. ^{13}C (100MHz in CD_3OD)- and ^{1}H (400MHz in CD_3OD)-NMR spectrum of compound **15**.

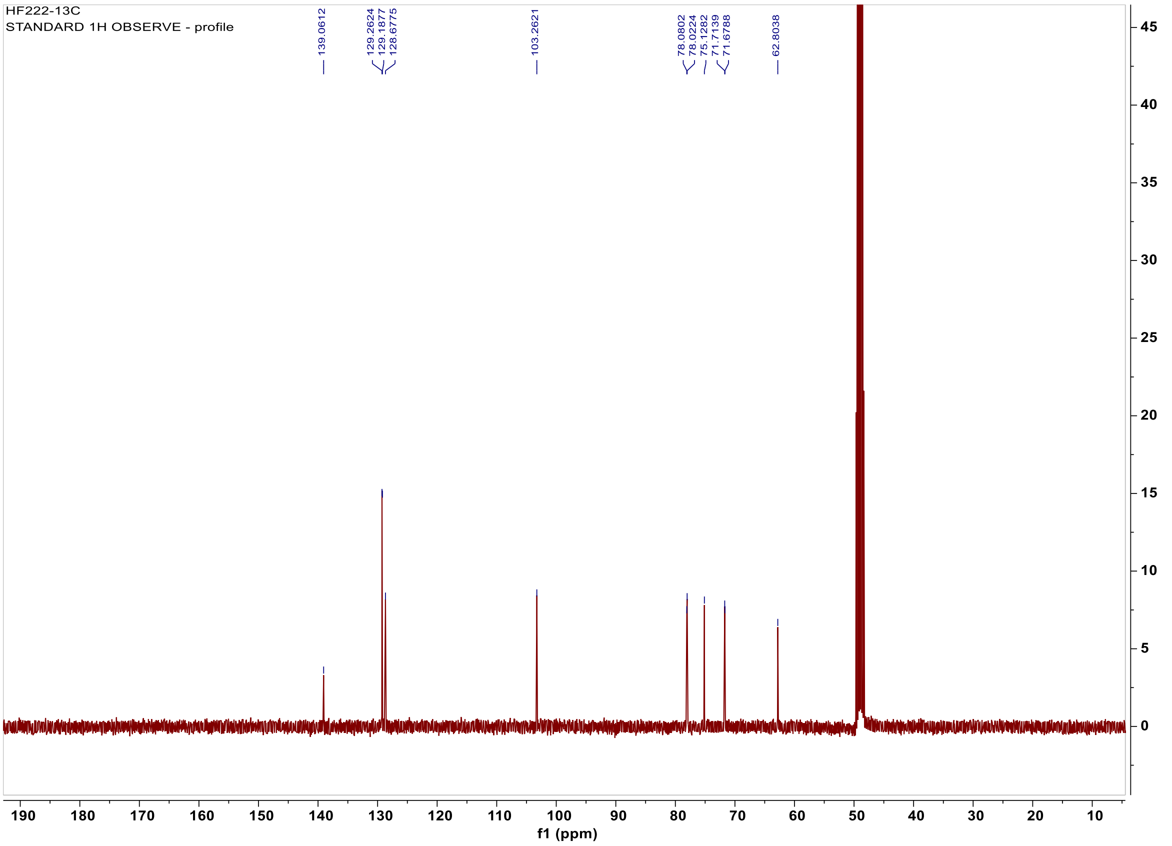

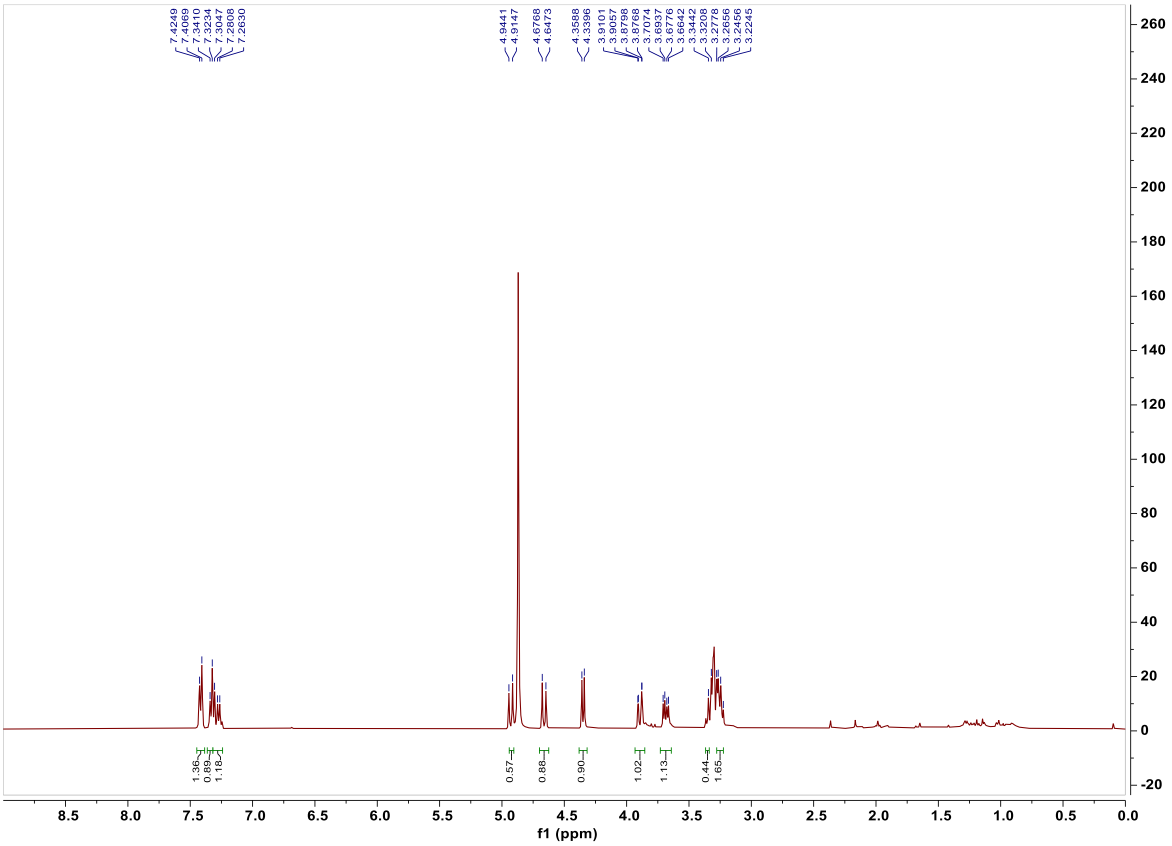

Figure S30. ${}^{13}C$ (100MHz in CD_3OD)- and ${}^{1}H$ (400MHz in CD_3OD)-NMR spectrum of compound **16**.

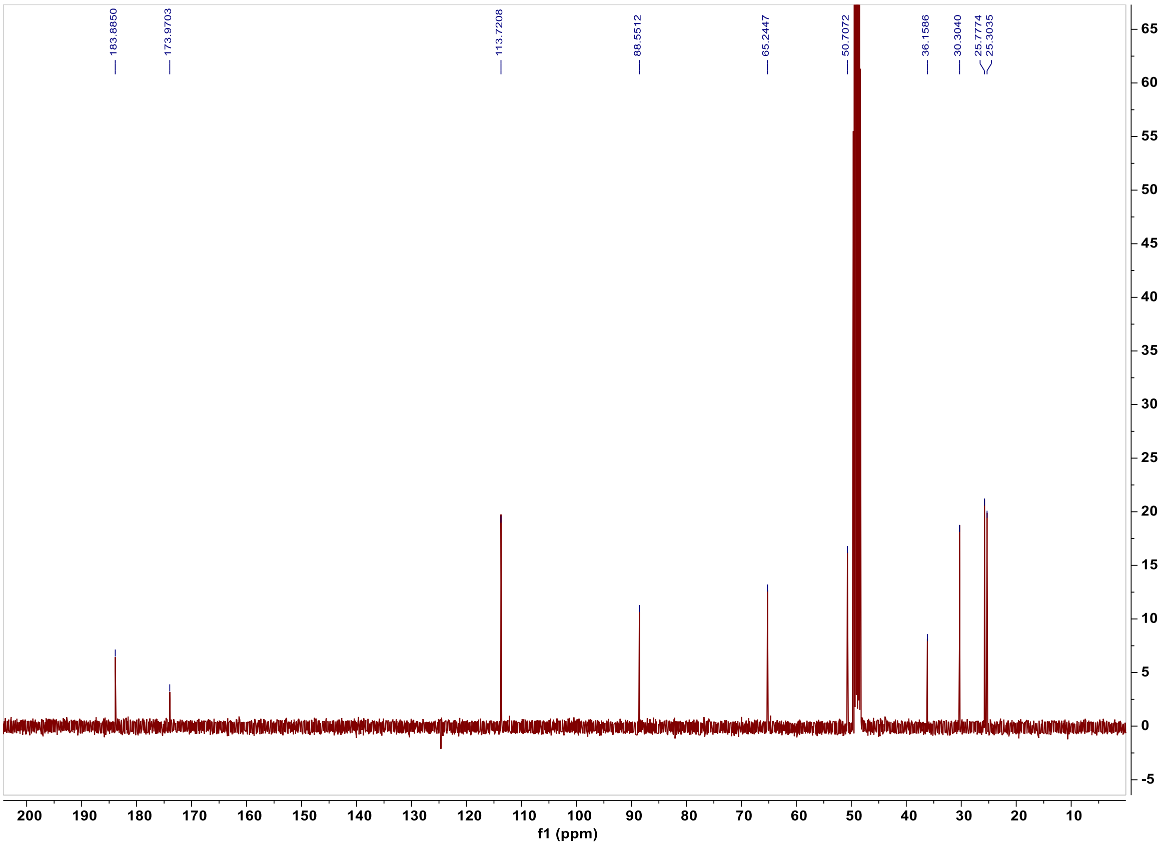

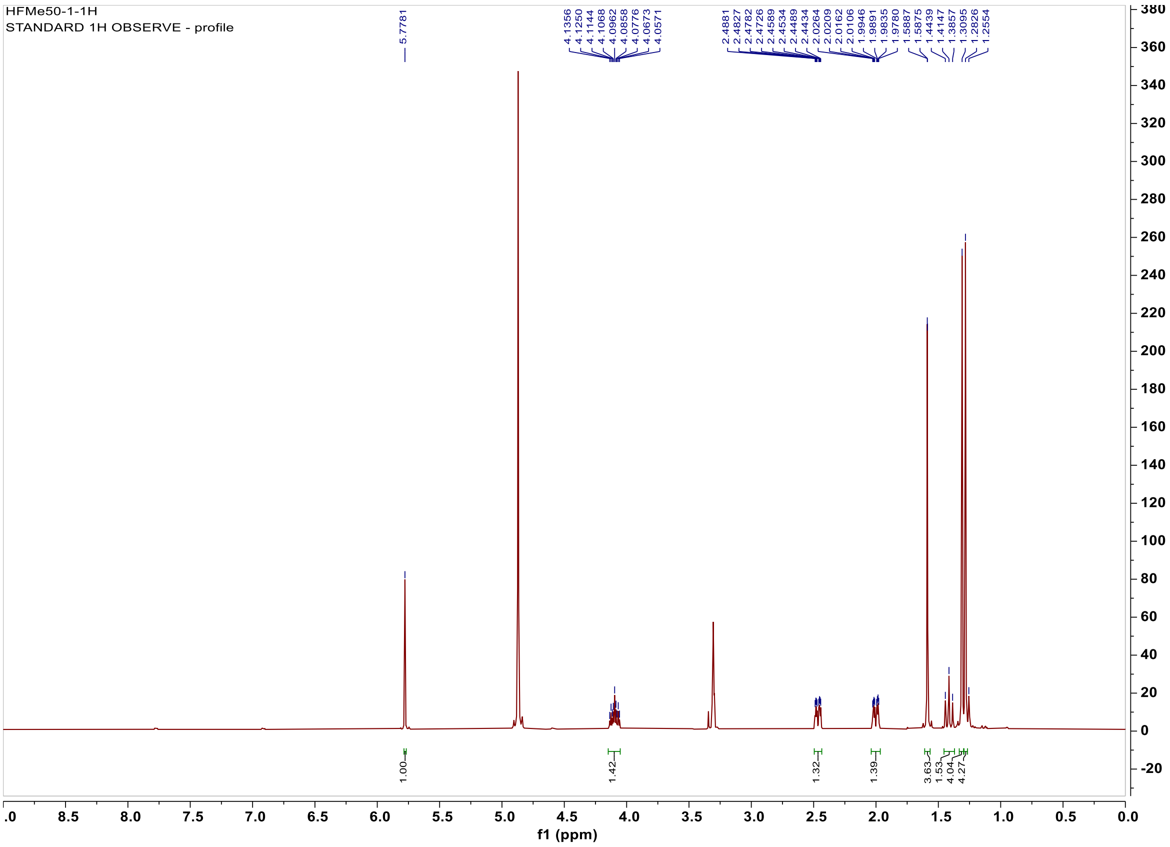

Figure S31. ^{13}C (100MHz in CD_3OD)- and ^{1}H (400MHz in CD_3OD)-NMR spectrum of compound **17**.

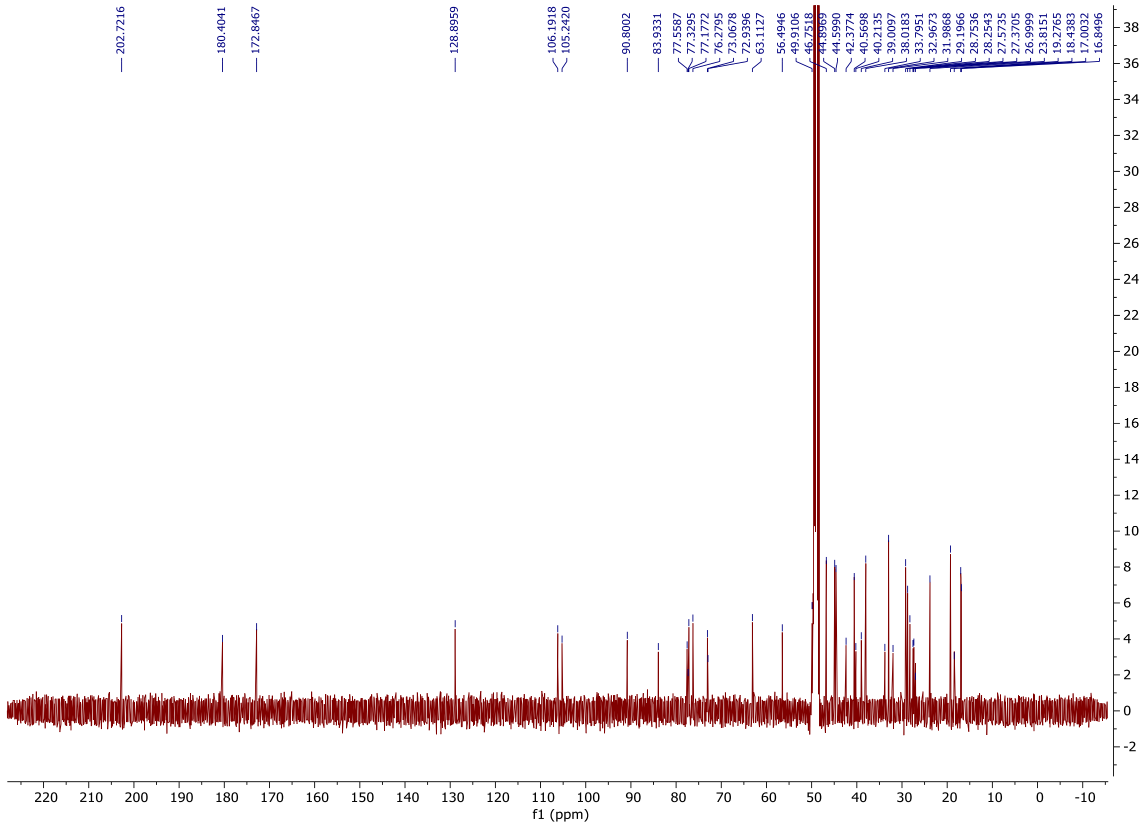

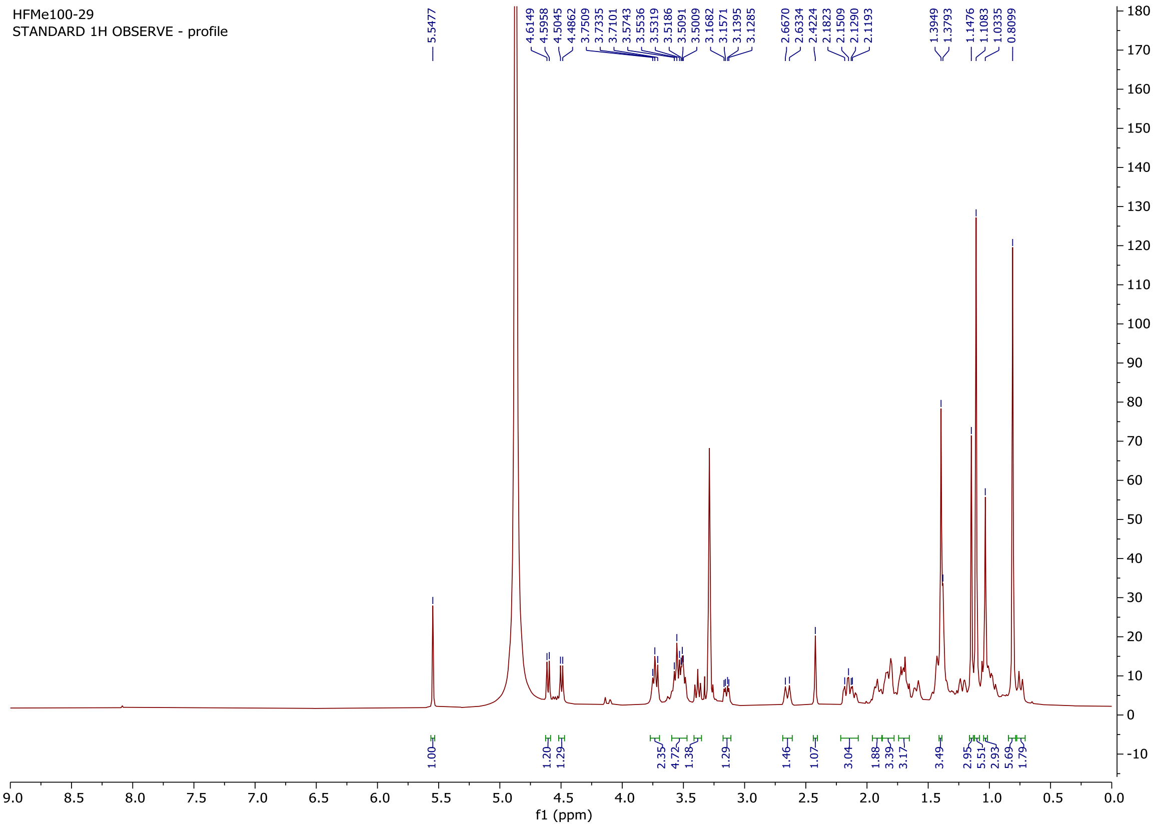

Figure S32. ^{13}C (100MHz in CD_3OD)- and ^{1}H (400MHz in CD_3OD)-NMR spectrum of compound **18**.

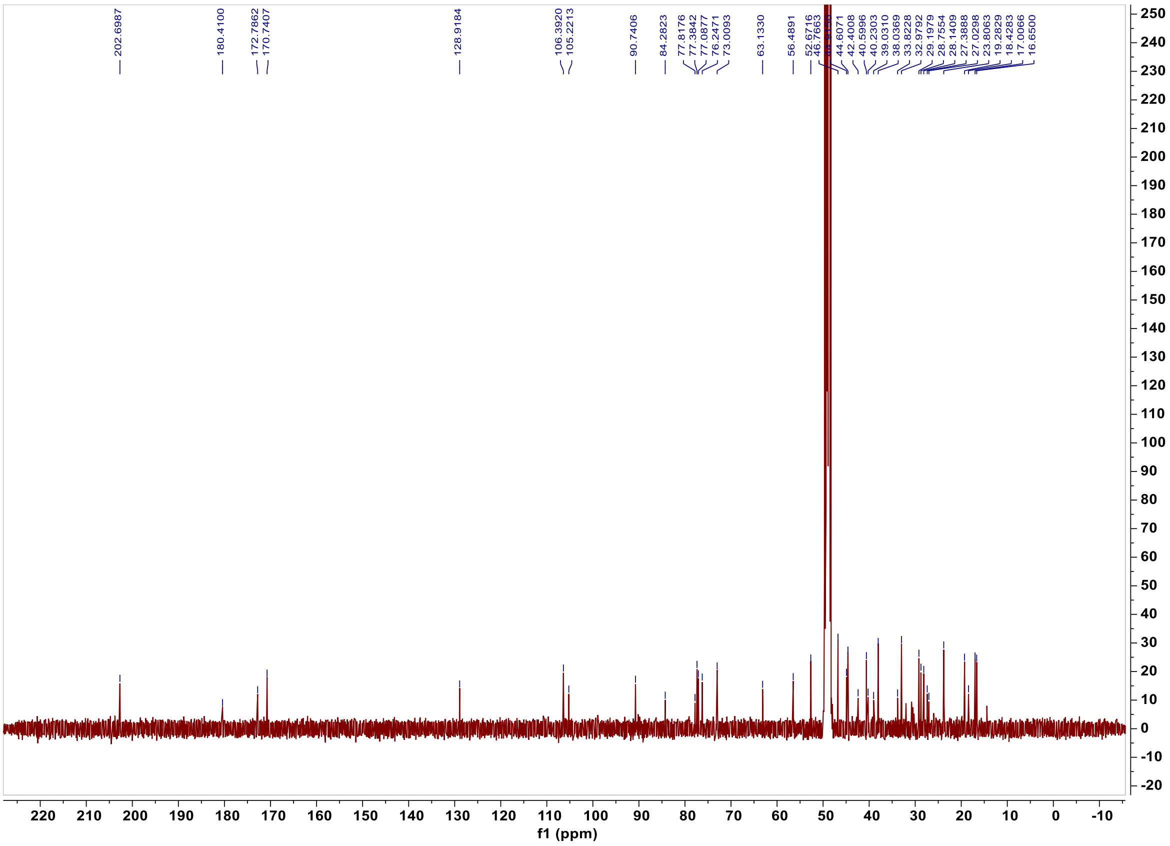

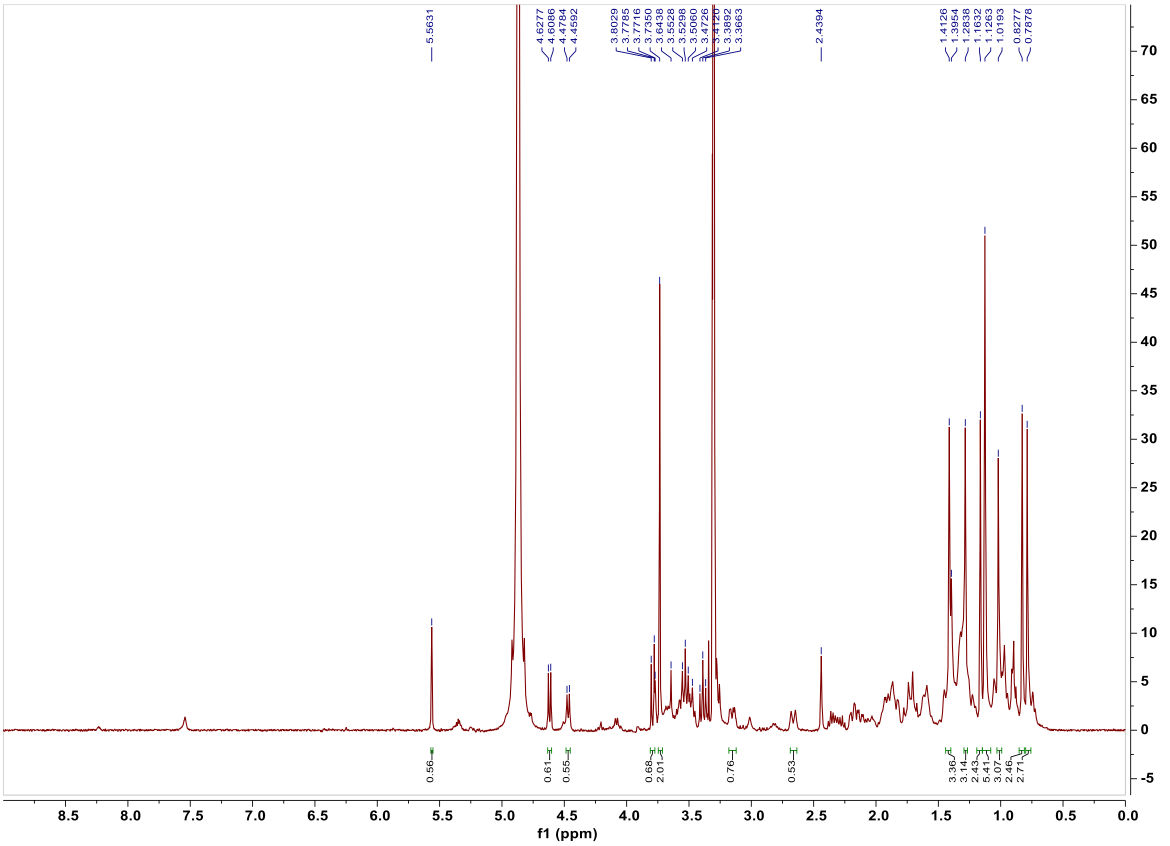

Figure S33. ^{13}C (100MHz in CD_3OD)- and ^{1}H (400MHz in CD_3OD)-NMR spectrum of compound **19**.

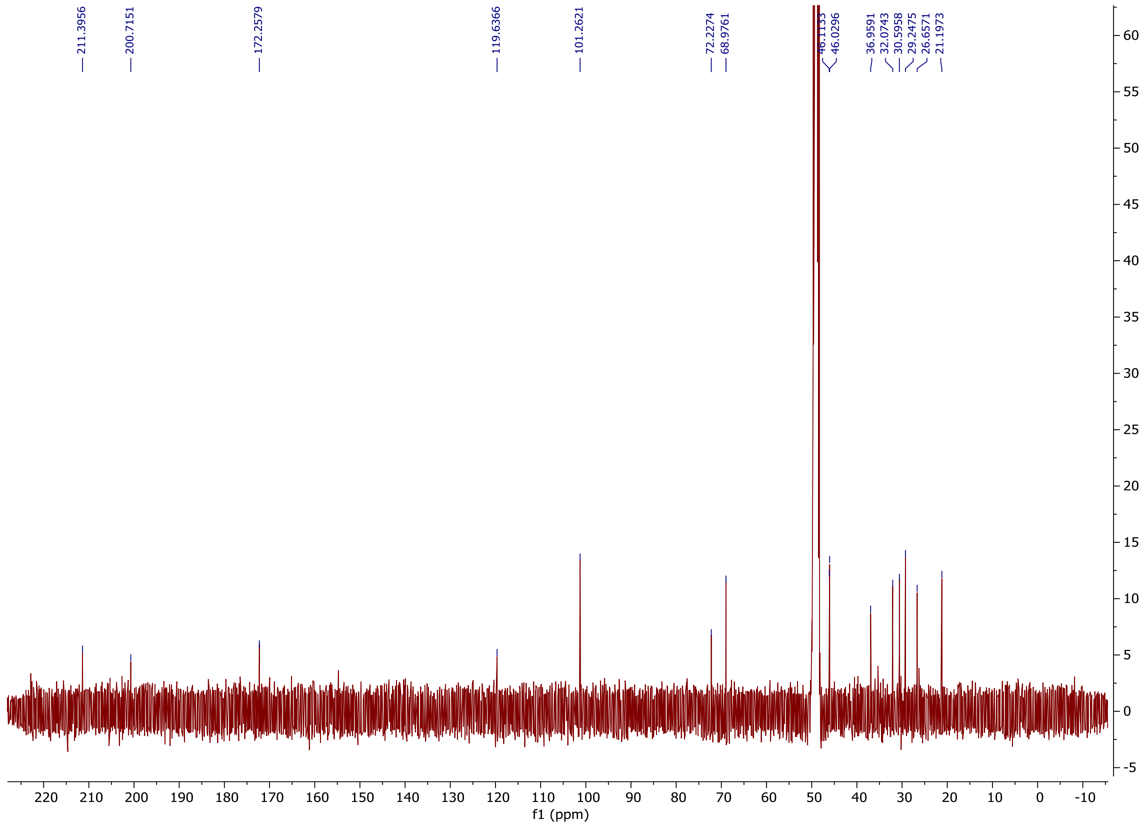

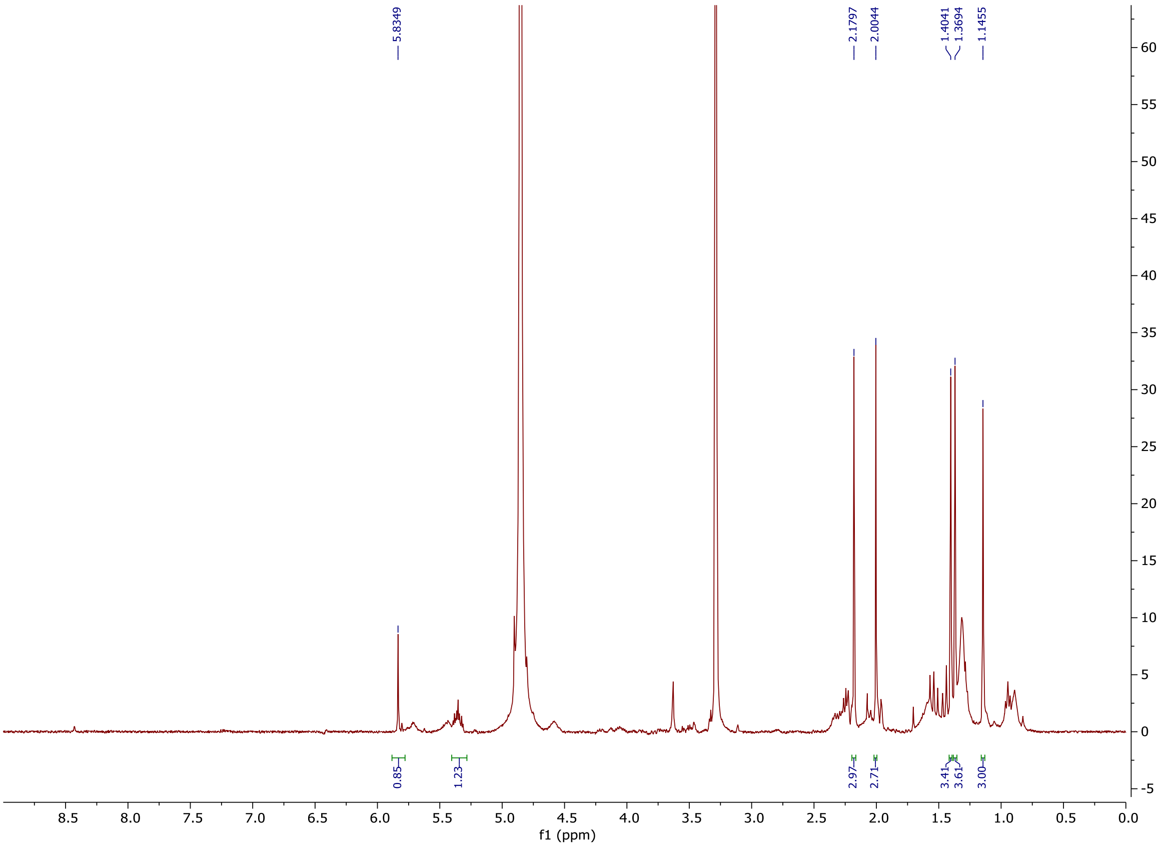

Figure S34. ^{13}C (100MHz in CD_3OD)- and ^{1}H (400MHz in CD_3OD)-NMR spectrum of compound **20**.

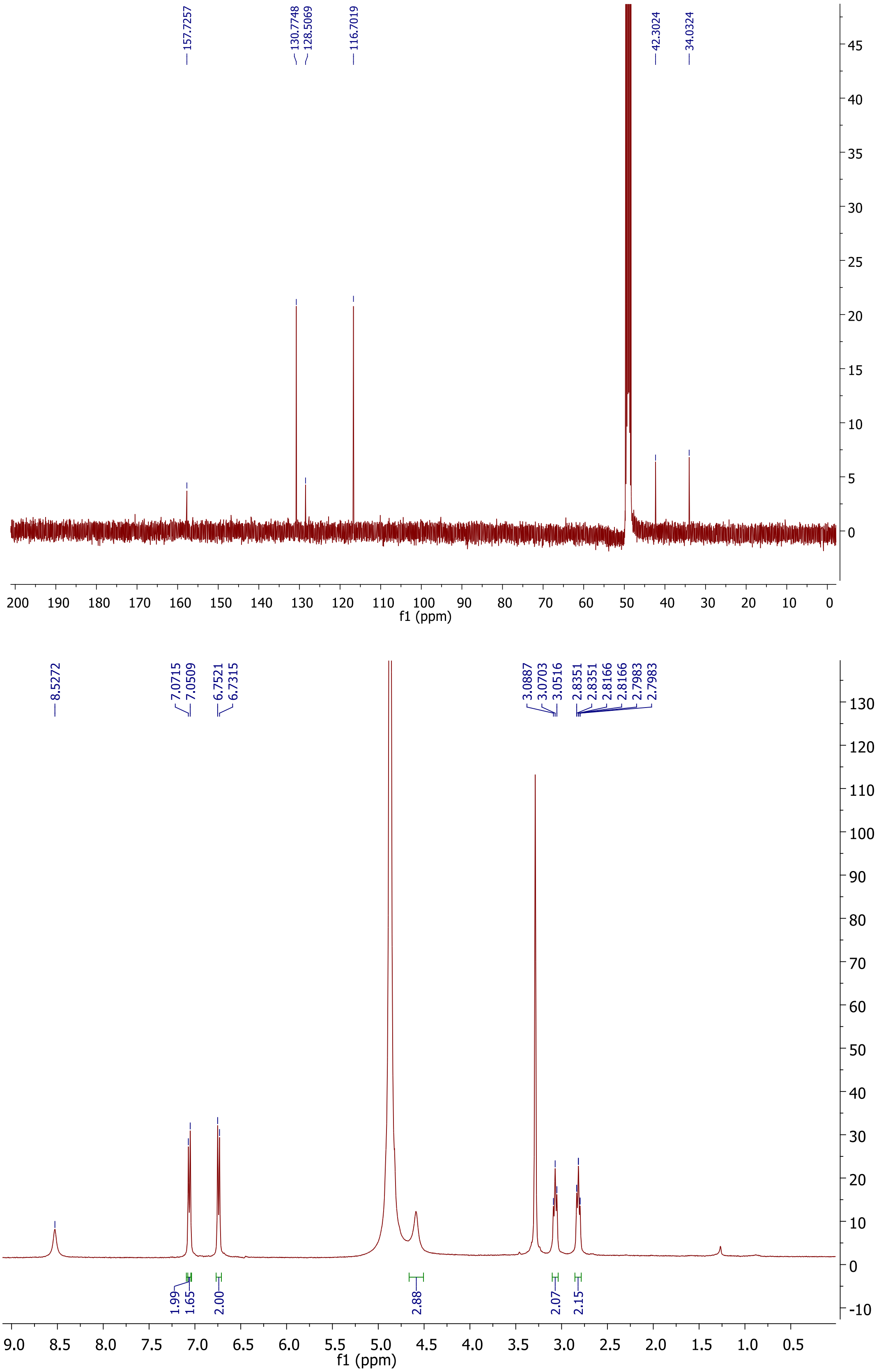

Figure S35. ^{13}C (100MHz in CD_3OD)- and ^{1}H (400MHz in CD_3OD)-NMR spectrum of compound **21**.

Table S1. Molecular weight and molecular formula of 21 isolated compounds.

**Compounds**	**Molecular formula**	**Observed molecular weight**	**Exact molecular weight**
**1**	$C_{16}H_{30}O_2$	254 [a]	254.2245
**2**	$C_{16}H_{32}O_2$	256 [a]	256.2402
**3**	$C_{18}H_{34}O_2$	282 [a]	282.2558
**4**	$C_{29}H_{48}O_2$	428 [a]	428.3654
**5**	$C_{29}H_{48}O$	412 [a]	412.3705
**6**	$C_{29}H_{48}O_2$	428 [a]	428.3654
**7**	$C_{17}H_{28}O_2$	264 [a]	264.2089
**8**	$C_{18}H_{30}O_2$	278 [a]	278.2245
**9**	$C_{20}H_{32}O_2$	304 [a]	304.2402
**10**	$C_{18}H_{28}O_2$	276 [a]	276.2089
**11**	$C_{20}H_{30}O_2$	302 [a]	302.2245
**12**	$C_{17}H_{28}O_2$	264 [a]	264.2089
**13**	$C_{34}H_{54}O_9$	607.3820 $[M + H]^+$ [b]	607.3846 ($C_{34}H_{55}O_9$)
**14**	$C_{19}H_{22}O_6$	– [c]	346.1416
**15**	$C_{19}H_{24}O_7$	– [c]	364.1522
**16**	$C_{13}H_{18}O_6$	– [c]	270.1103
**17**	$C_{11}H_{16}O_3$	219.0992 $[M + Na]^+$ [b]	219.0997 ($C_{11}H_{16}NaO_3$)
**18**	$C_{42}H_{62}O_{16}$	– [c]	822.4037
**19**	$C_{43}H_{64}O_{16}$	– [c]	836.4194
**20**	$C_{15}H_{22}O_4$	– [c]	266.1518
**21**	$C_8H_{11}NO$	– [c]	137.0840

[a] Observed from EI-MS.

[b] Observed from ESI-MS.

[a] Structures were elucidated based on the NMR data.
